# Supplementary material for: Endoscopic healing in pediatric IBD perpetuates a persistent signature defined by Th17 cells with molecular and microbial drivers of disease
Source: Cell Rep Med. 2025 Jul 15;6(7):102236. doi: 10.1016/j.xcrm.2025.102236 (PMC12281438; doi:10.1016/j.xcrm.2025.102236)
Supplement: Document S1. Figures S1–S14 [file mmc1.pdf]

Cell Reports Medicine, Volume *6*

Supplemental information

Endoscopic healing in pediatric IBD perpetuates a persistent signature defined by Th17 cells with molecular and microbial drivers of disease

Kolja Siebert, Tim Faro, Nikolai Köhler, Hannes Hölz, Sebastian Jarosch, Monica Matchado, Deborah Häcker, Federica De Zen, Mohammad Samer Hajji, Eberhard Lurz, Sibylle Koletzko, Josch K. Pauling, Katja Steiger, Klaus Neuhaus, Caspar Ohnmacht, Markus List, Dirk H. Busch, Dirk Haller, and Tobias Schwerd

Supplemental information

Table of Contents

Figure S1-2: Bulk RNA sequencing, related to Figure 1

Figure S3: Immunohistochemistry, related to Figure 1

Figure S4: External single cell data, related to Figure 1

Figure S5-6: Bulk RNA sequencing, related to Figure 1

Figure S7-8: Single T-cell RNA sequencing, related to Figure 2

Figure S9-10: 16S rRNA genes of mucosa associated microbiota, related to Figure 4

Figure S11-14: IBrD signature, related to Figure 5

Figure S1

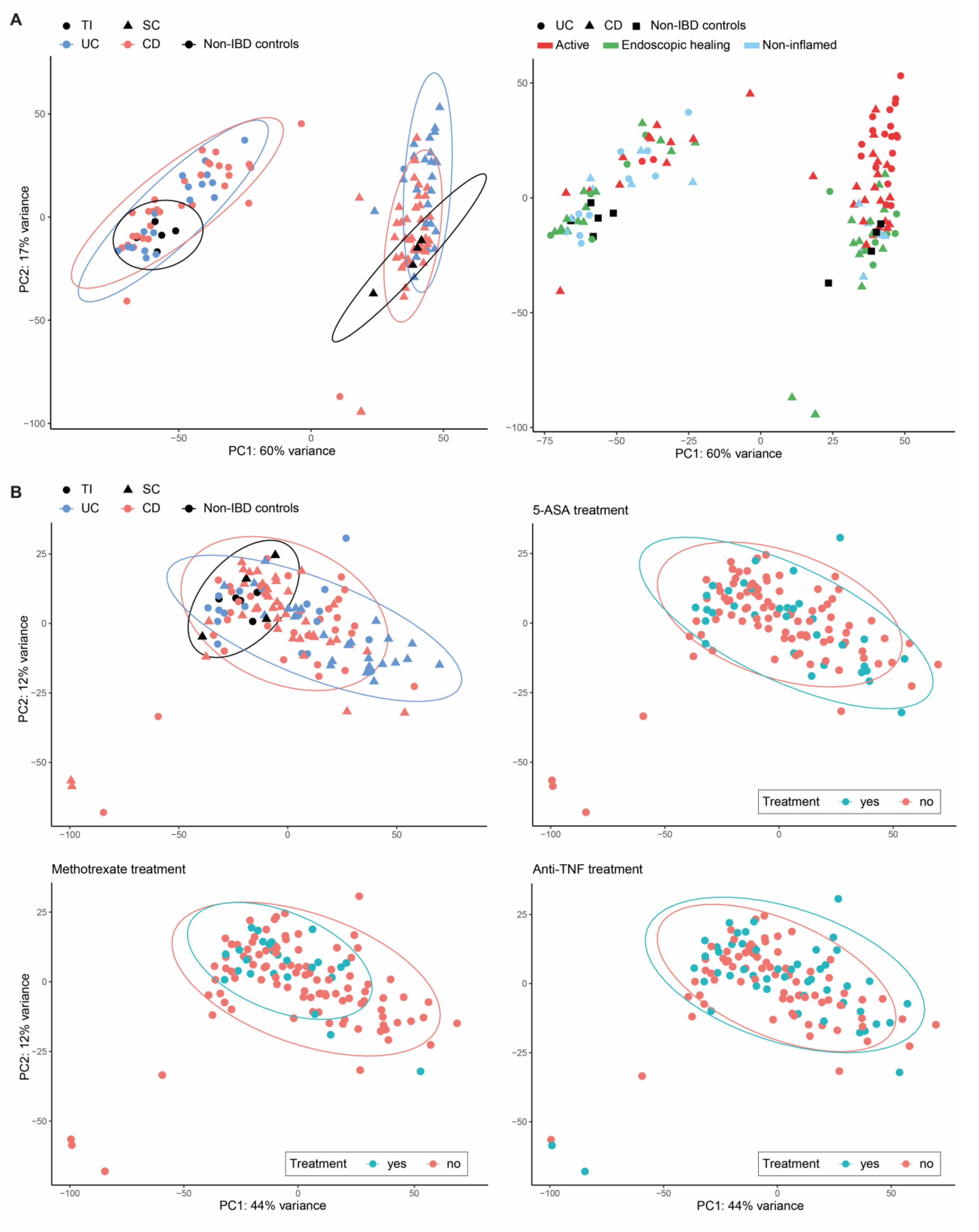

Figure S1. ***(A)*** *Principal component analysis (PCA, batch corrected) showing distribution of bulk RNA sequencing samples (samples n = 127; IBD patients, N = 30; non-IBD controls, N = 5) with (left) location (terminal ileum (TI) and sigmoid colon (SC)) and (right) endoscopic activity highlighted. Disease phenotype is shown in both.* ***(B)*** *PCA (after correction for location and batch), labeled with CD, UC and non-IBD controls, location and most common treatments. Related to Figure 1.*

Figure S2

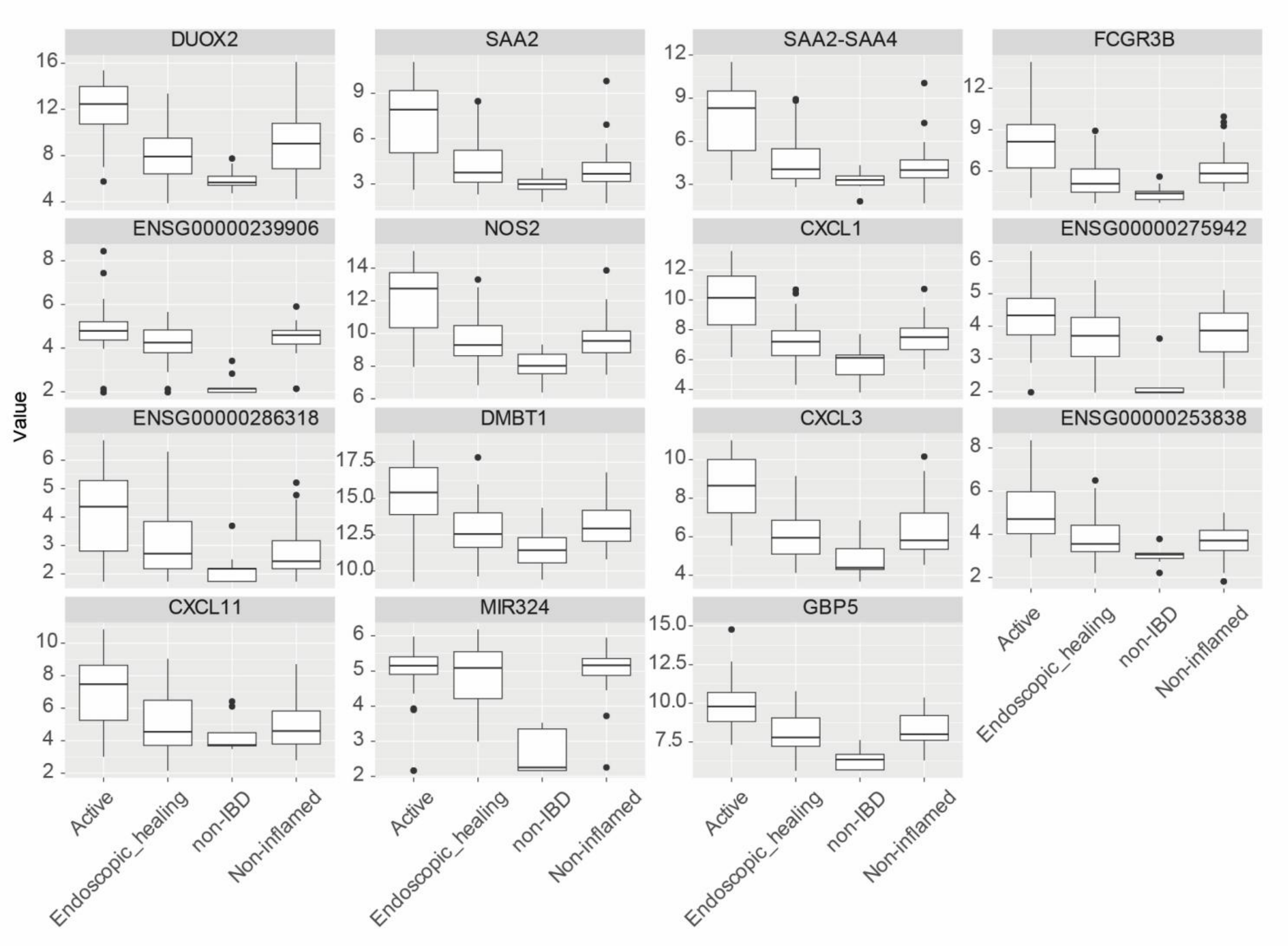

Figure S2. *Boxplots of top 15 up-regulated persistent genes according to endoscopic IBD activity (active, endoscopic healing (EH), non-inflamed) and compared to non-IBD controls (samples n = 127; IBD patients, N = 30; non-IBD controls, N = 5). Related to Figure 1.*

Figure S3

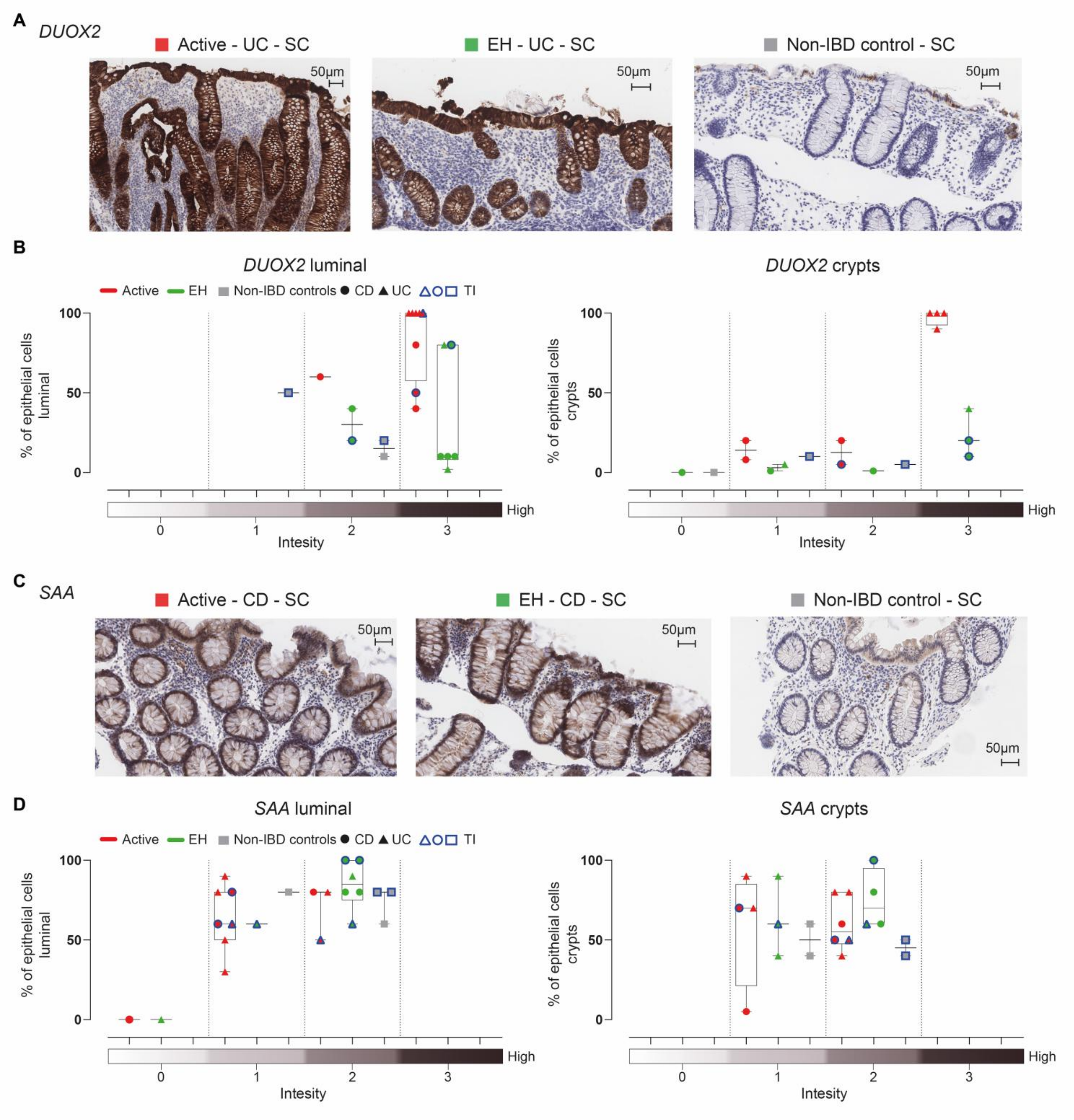

Figure S3. ***(A-B)*** *Immunohistochemistry (IHC) of DUOX2 in IBD patients (N=9, active (n=9) and endoscopic healing (EH, n=8)) and non-IBD controls (n=3).* ***(A)*** *Representative visualization of the IHC in an UC patient (sigmoid colon (SC), active and EH) and a non-IBD control.* ***(B)*** *Semi-quantification of staining intensity (from 0 [no staining] to 3 [high]) and frequency of epithelial cells (active: red; EH: green, non-IBD controls: gray, CD: circle, UC: triangle, terminal ileum (TI): blue frame, SC: no blue frame) differentiated by localization of cells in luminal (left panel) or crypts (right panel). Semi-quantification was performed by a blinded pathologist.* ***(C-D)*** *IHC of SAA.* ***(C)*** *Representative visualization of the IHC in a CD patient (SC, active and EH) and a non-IBD control.* ***(D)*** *Semi-quantification as described in (B) for IBD patients (N=9, active (n=11) and EH (n=8)) and non-IBD controls (n=4). Related to Figure 1.*

Figure S4

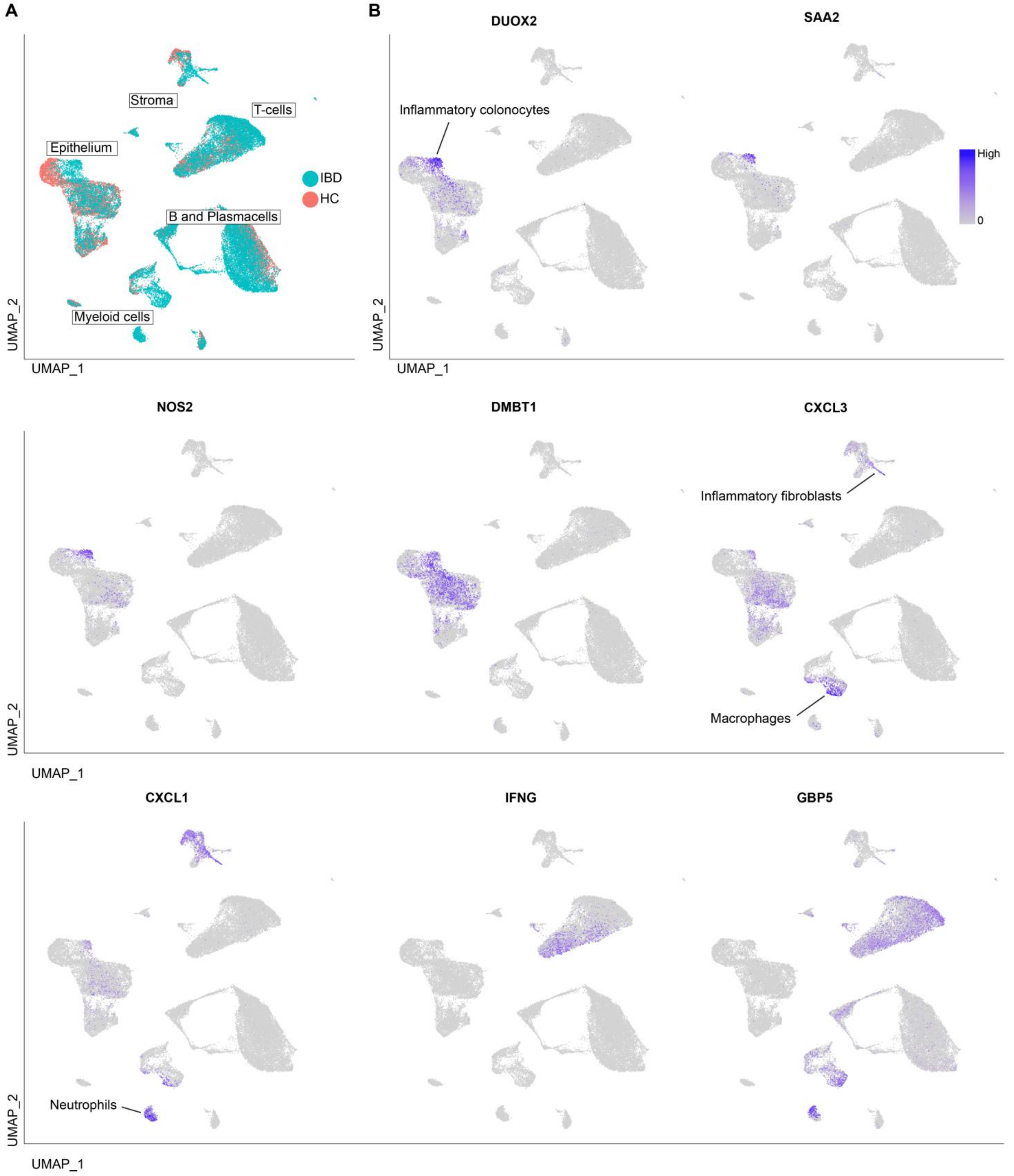

Figure S4. *External single cell data from Garrido-Trigo et al. (2023); graphs were generated at https://servidor2-ciberehd.upc.es/external/garrido/app/, accessed 08.09.2024.* ***(A)*** *The Uniform Manifold Approximation and Projection (UMAP) demonstrates the clustering of epithelial cells, T-cells, myeloid cells, stromal cells, B and plasma cells. Cells are from IBD patients (turquoise) and healthy controls (HC, red). (**B**) Feature plots show selected gene expressions of cells across the UMAP (from A). Related to Figure 1.*

Figure S5

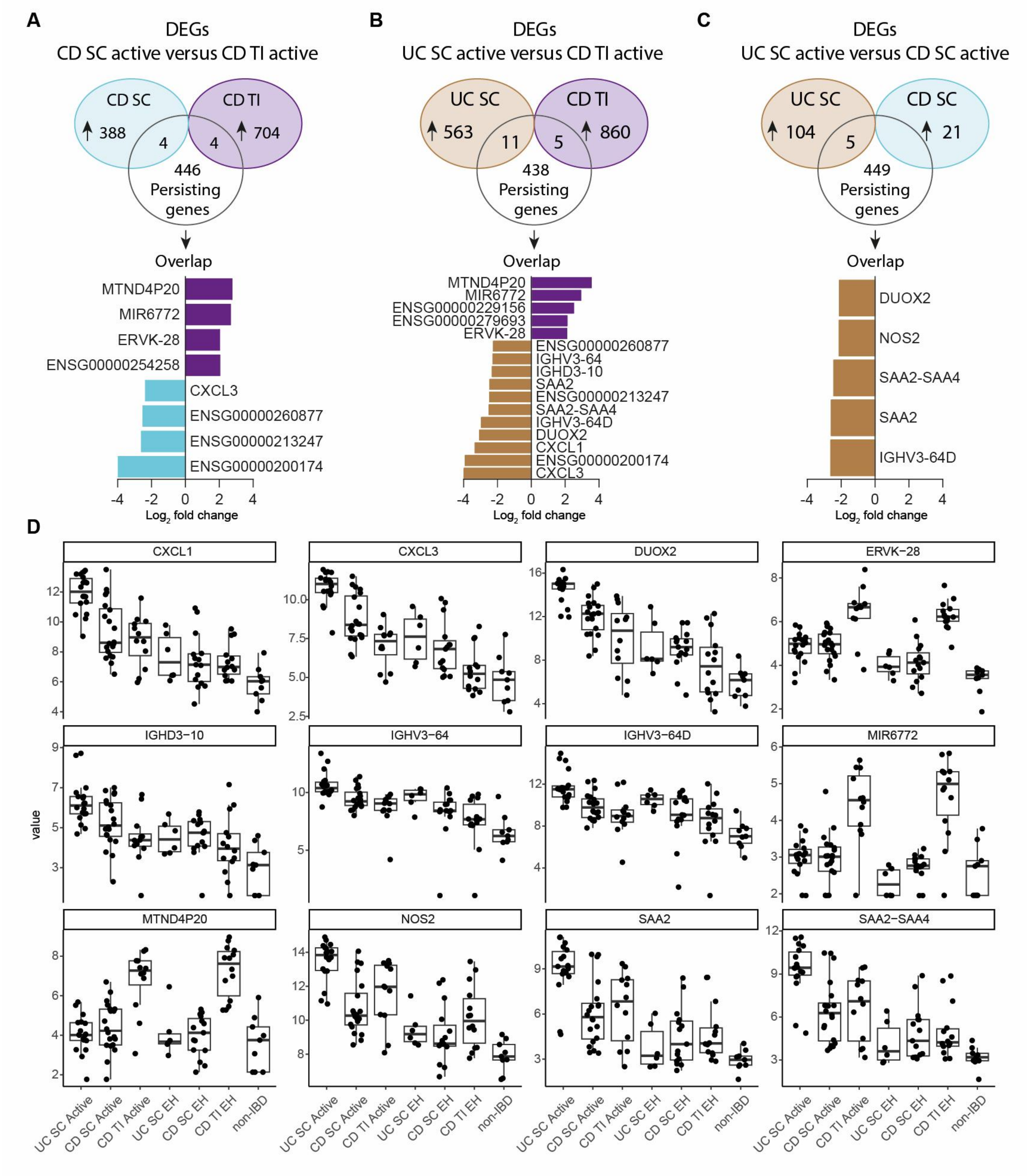

Figure S5. (A-C*)* *Testing for differentially expressed genes (DEGs; log2FC ≥ 2, ≤ -2 and p.adj < 0.05, Benjamini-Hochberg corrected) in our bulk RNA sequencing data between* ***(A)*** *CD samples with active disease at the terminal ileum (TI) versus CD with active disease at the sigmoid colon (SC),* ***(B)*** *CD TI active versus UC SC active and* ***(C)*** *CD SC active versus UC SC active. The DEGs that overlap with the persistent up-regulated genes (Figure 1G) are highlighted below.* ***(D)*** *The box plots show the expression levels of the annotated genes highlighted in A, B and C across disease subtypes (samples n = 94; IBD patients, N = 30; non-IBD controls, N = 5) and endoscopic activity: active, endoscopic healing (EH) and non-IBD controls (non-IBD). Related to Figure 1. See also Table S4-6.*

Figure S6

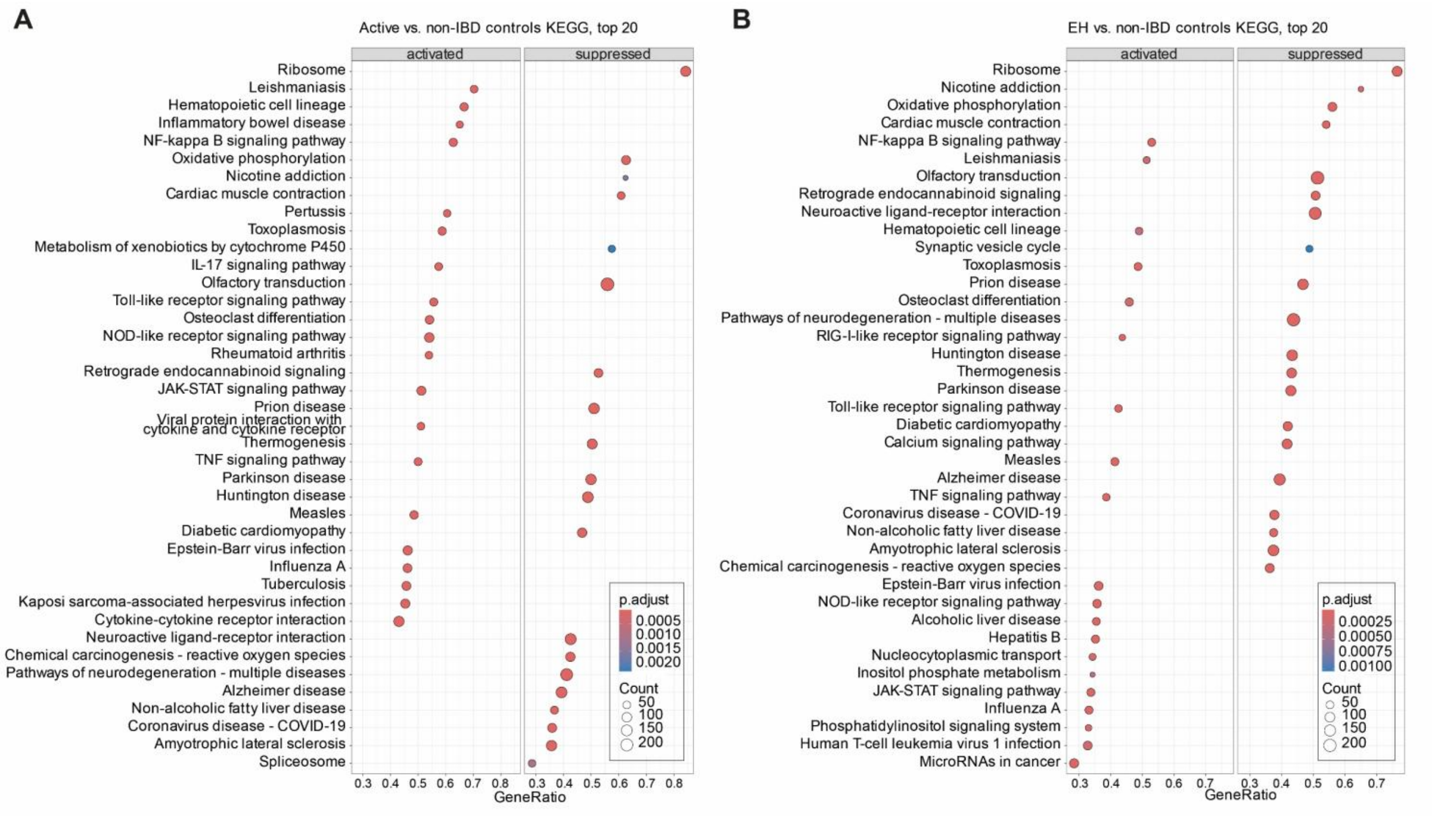

Figure S6. *Gene set enrichment analysis of **(A)** active vs. non-IBD controls and **(B)** endoscopic healing (EH) vs. non-IBD controls. Related to Figure 1.*

Figure S7

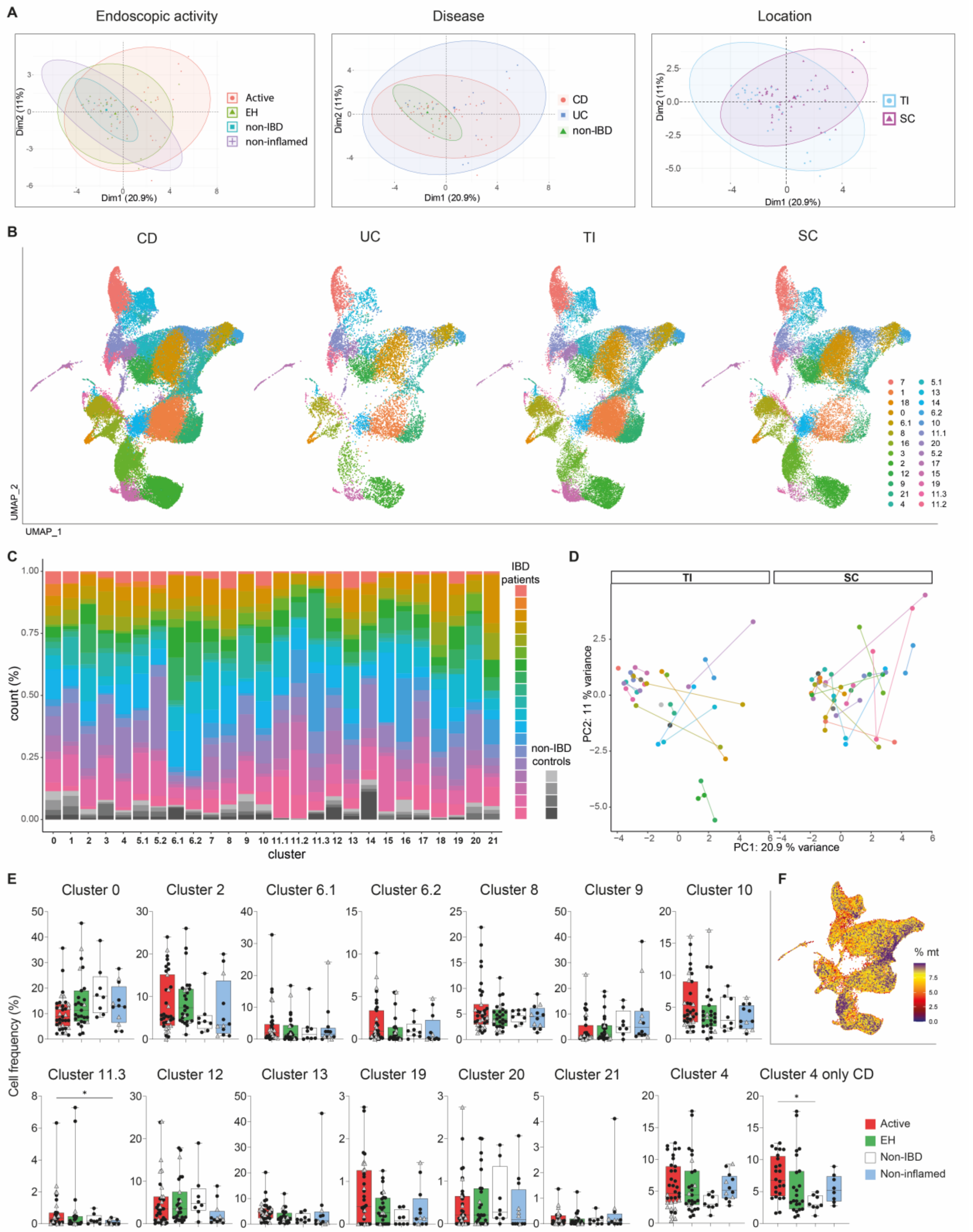

Figure S7. ***(A)*** *Principal component analysis (PCA, n = 82; IBD patients, N = 19; non-IBD controls, N = 4) showing distribution of samples according to single cell frequencies and (from left to right) endoscopic disease activity, IBD phenotype and location.* ***(B)*** *Distribution of cells highlighted for Crohn's disease (CD), ulcerative colitis (UC), terminal ileum (TI) and sigmoid colon (SC) across the UMAP (from Figure 2A).* ***(C)*** *Bar plot showing distribution of patients across all T-cell clusters. There was no over-representation of certain clusters in individual patients.* ***(D)*** *PCA of single T-cell frequencies, showing individual changes over time for each location (TI, SC).* ***(E)*** *Boxplots showing relative cell frequencies per cluster and biopsy (per individual biopsy: number of cells per cluster/total cell number per biopsy, CD: circles, UC: gray triangles) according to endoscopic activity groups; cluster 4 is shown additionally for CD only. Statistical tests: Kruskal-Wallis test followed by comparing mean ranks between individual columns and multiple testing correction using Benjamini-Hochberg, p.adj < 0.05, *=significant; non-significant is not labeled.* ***(F)*** *Feature plot showing percent of mitochondrial (mt) gene expression (QC cut-off: 10%) across T-cell clusters. Related to Figure 2.*

Figure S8

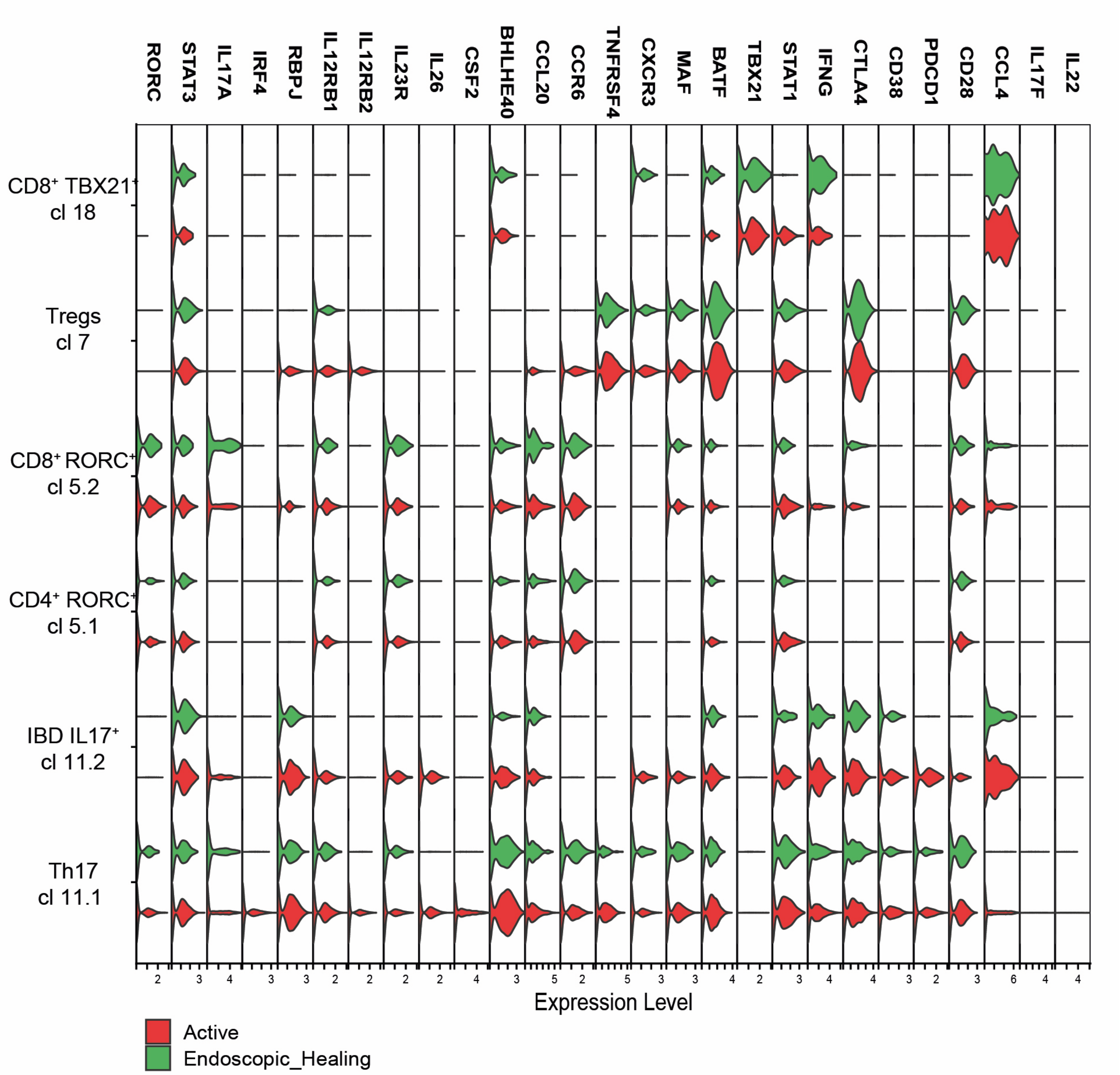

Figure S8. *Violin plot showing gene expression of selected clusters and selected genes according to disease activity groups (active in red; endoscopic healing in green). Related to Figure 2.*

Figure S9

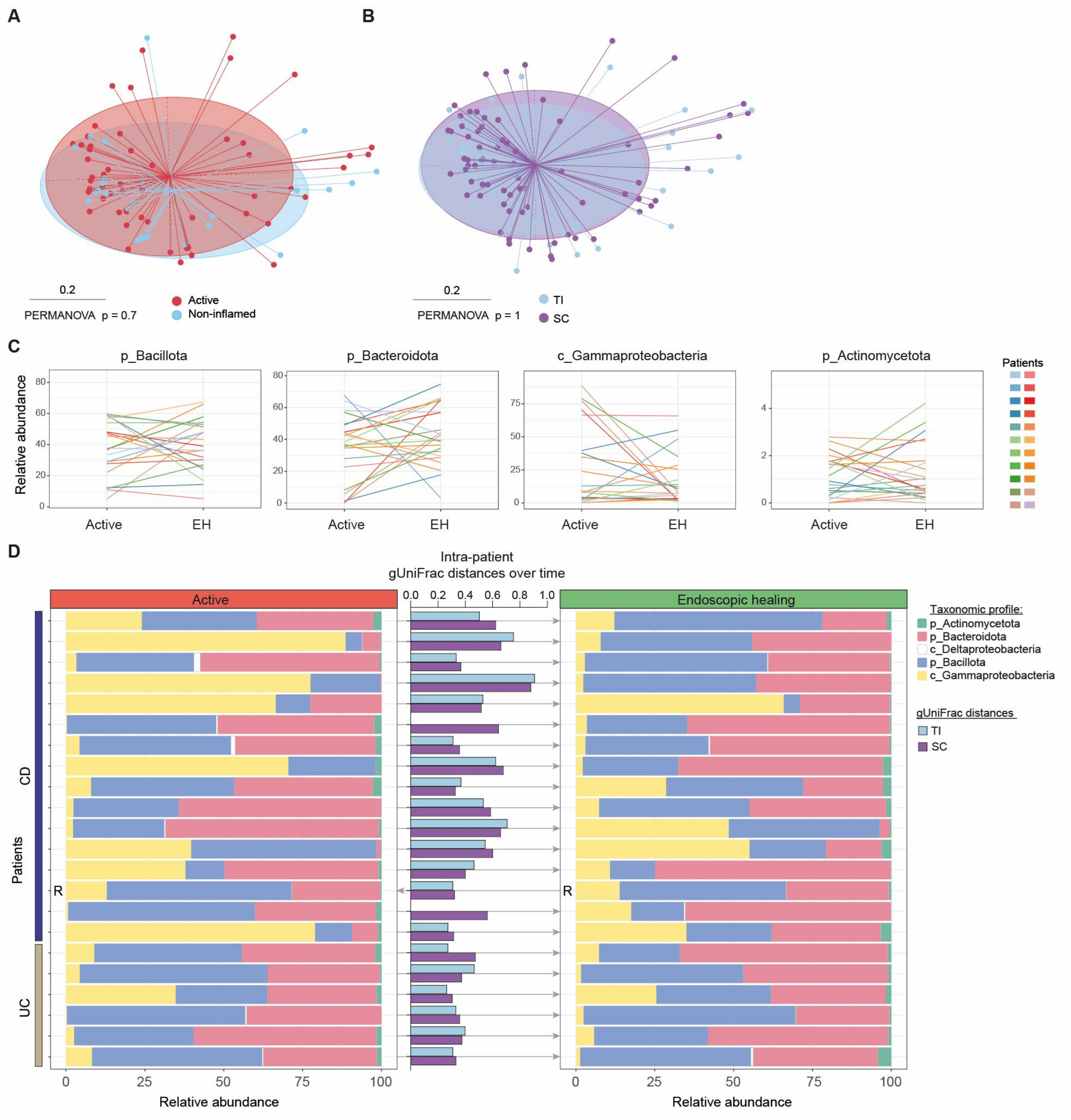

Figure S9. ***(A-B)*** *Beta-diversity MDS plots of* ***(A)*** *active (n=56) vs. non-inflamed (non-inflamed region in an active disease state, n=26) IBD and of* ***(B)*** *anatomic location (terminal ileum (TI, n=62) and sigmoid colon (SC, n=73)).* ***(C)*** *Time-series analysis between active and EH of individual patients (N=22) of Bacillota, Bacteroidota, Actinomycetota and Gammaproteobacteria, showing the mean (TI and SC) relative abundance for active and EH, respectively.* ***(D)*** *Mean taxonomic profile changes of individual patients between active and EH (N=22) as color-coded stacked bar plots. In addition, intra-patient generalized UniFrac (gUniFrac) distances with location specific changes between active and EH are shown. The index patient, who experienced a relapse (T_{F1}) within 10 weeks after discontinuing anti-TNF medication following EH (T_{BL}) is highlighted with an R. For the CD patient who first reached EH at T_{F2}, T_{BL} and T_{F2} are shown. Related to Figure 4.*

Figure S10

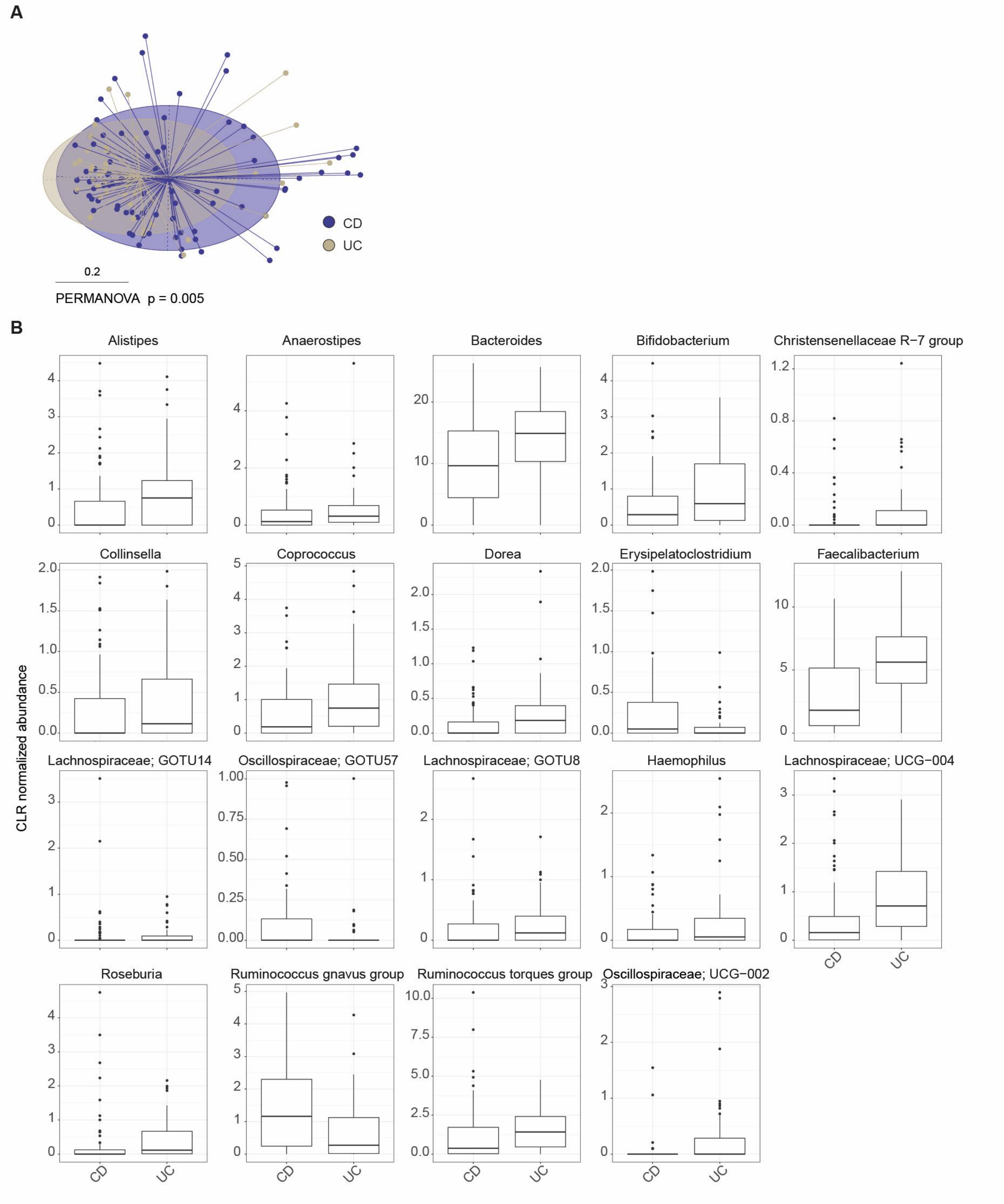

Figure S10 (A) *Beta-diversity MDS plot (generalized UniFrac distances) of IBD phenotype groups (CD, n=82; UC, n=43).* ***(B)*** *Boxplots showing centered log-ratio (CLR) transformed abundance of significantly different genera between CD and UC (Wilcoxon test, significance cutoff = 0.05). Related to Figure 4.*

Figure S11

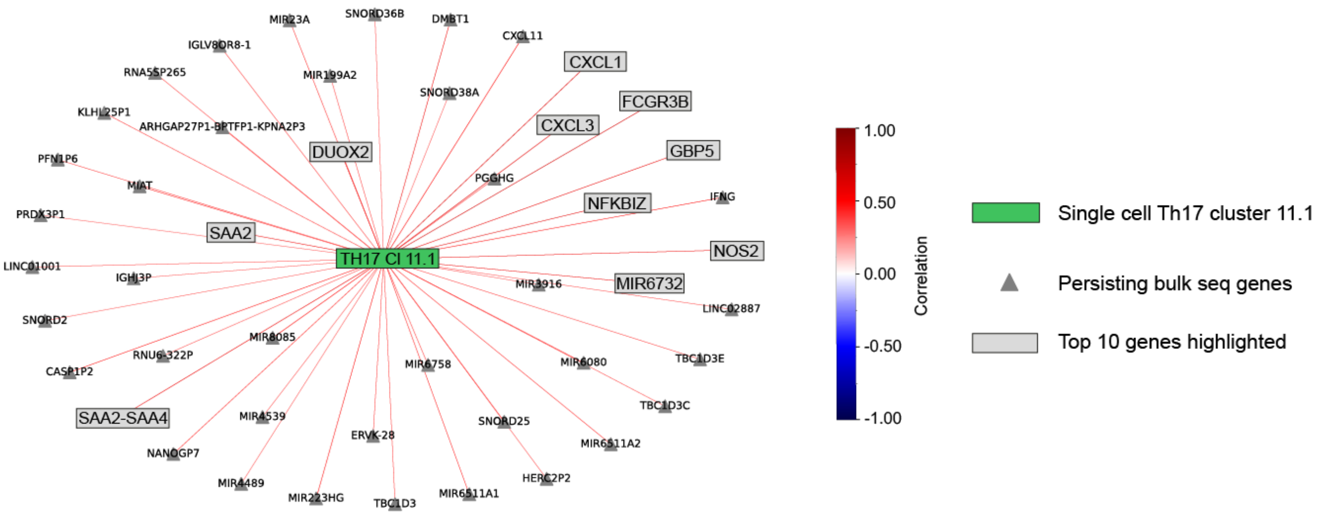

Figure S11. *Correlation network between Th17 cells (cluster 11.1 from Figure 2) and 282 persistently up-regulated genes (with official gene symbol) from bulk RNA sequencing (Figure 1G): nodes, identified significant features (top 10 genes according to correlation and p.adj-values are highlighted in gray boxes); edges, significant (Spearman) correlations between features of the two modalities (red, positive; blue, negative). Related to Figure 5.*

Figure S12

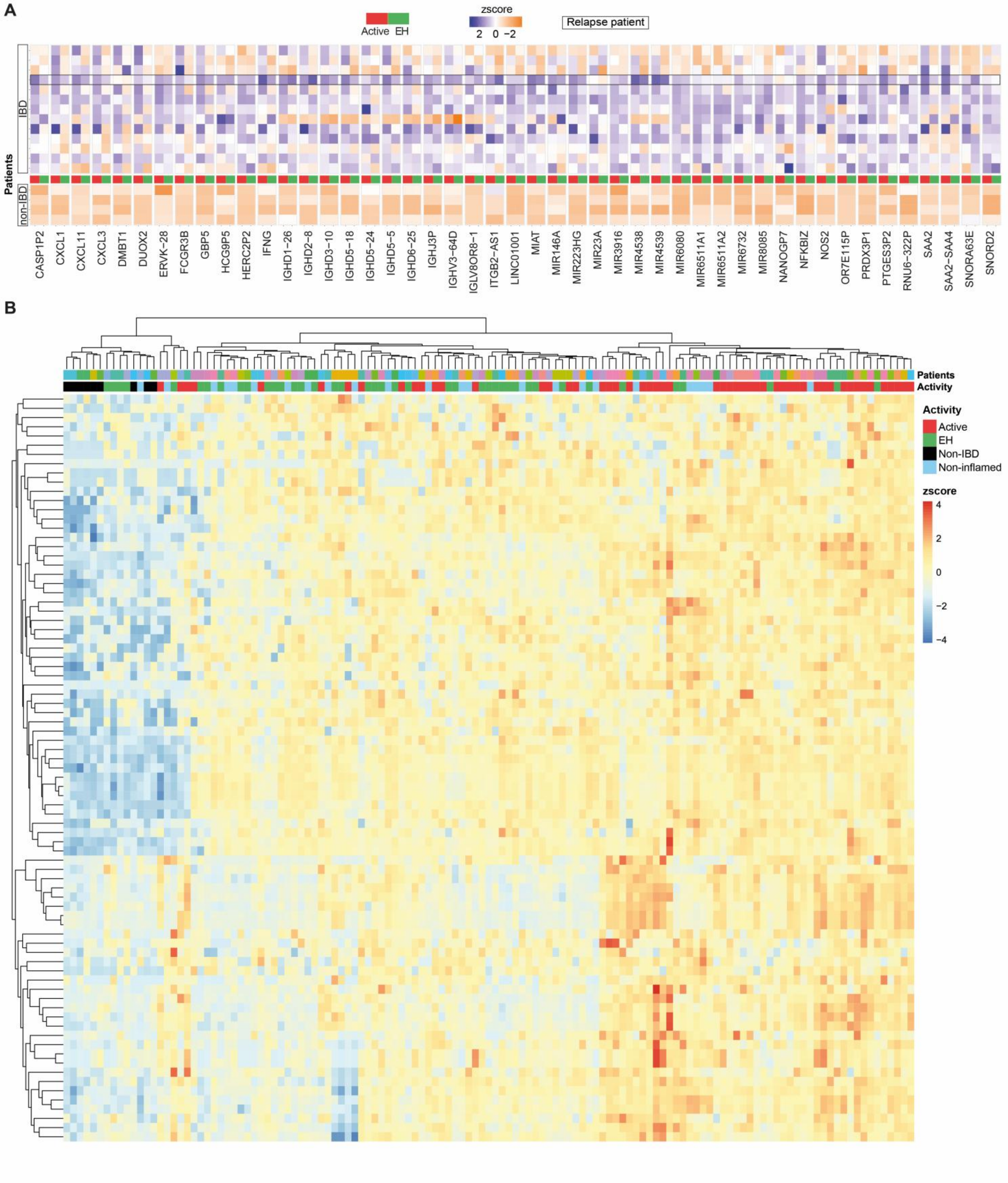

Figure S12. ***(A)*** *Individual expression of IBrD signature genes (>1 edge, from Figure 5B) in IBD patients and in non-IBD controls. For different anatomical locations, but with corresponding disease activity (e.g., active), the average value was computed. Subsequently, mean gene expression values were z-score scaled for visualization.* ***(B)*** *The heatmap of our bulk RNA sequencing samples (samples n = 127; IBD patients, N = 30; non-IBD controls, N = 5) illustrates the expression levels of the 81 IBrD signature genes across individual biopsies of patients, including hierarchical clustering. The biopsy origin according to patient and disease activity is highlighted. Related to Figure 5.*

Figure S13

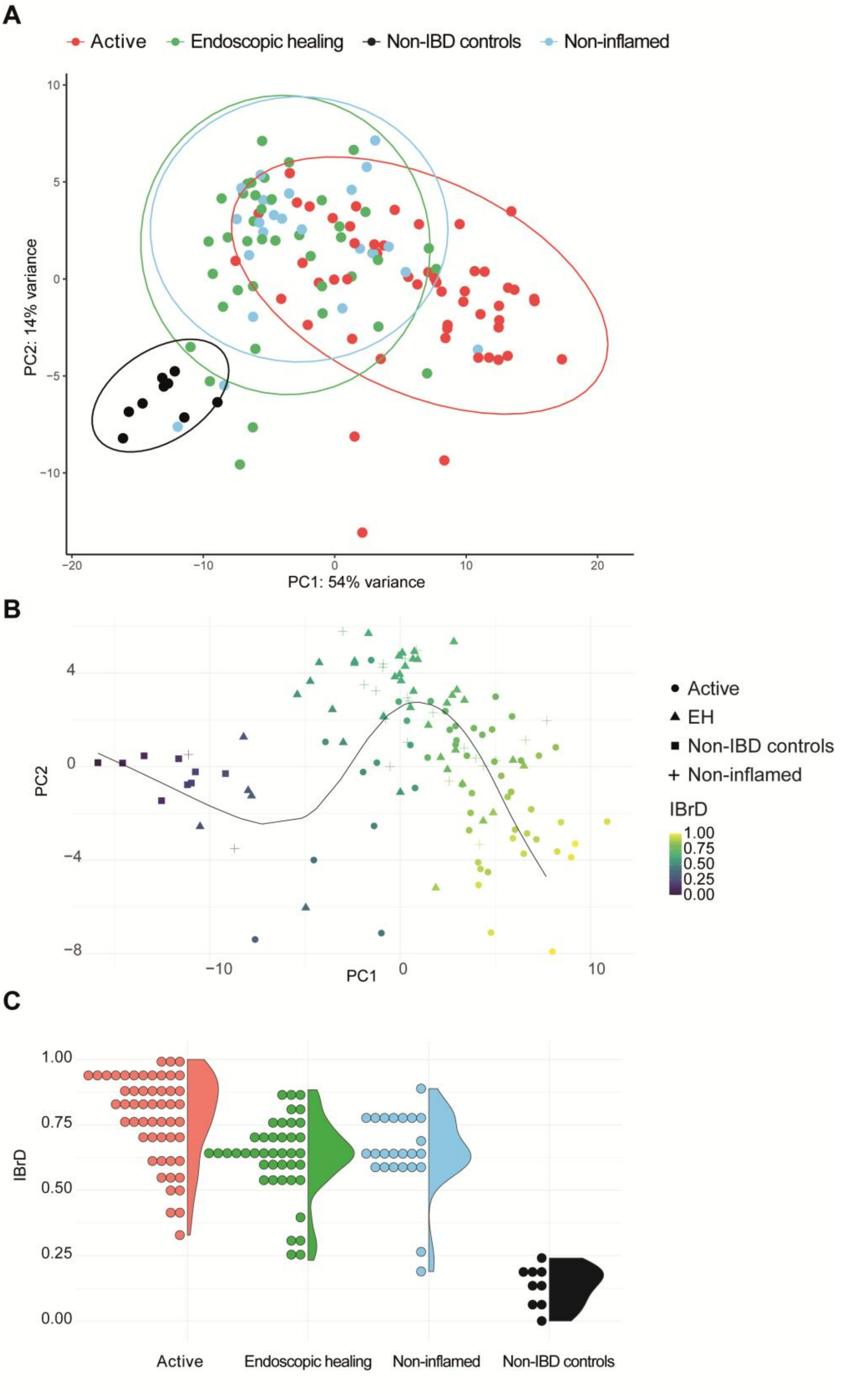

Figure S13. ***(A)*** *Principal component analysis (PCA, corrected for batch and location) showing distribution of bulk RNA sequencing samples (n=127) based on the IBrD signature.* ***(B)*** *PCA as in Figure 5D, including all bulk RNA samples (n=127), with color code indicating the Inflammatory Bowel residual Disease (IBrD, detailed description of how the IBrD was calculated for all samples are provided in the methods) scaled between 0 to 1, where 1 indicates highest activity.* ***(C)*** *IBrD values (interval between 0 and 1) for each sample according to endoscopic activity. Related to Figure 5.*

Figure S14

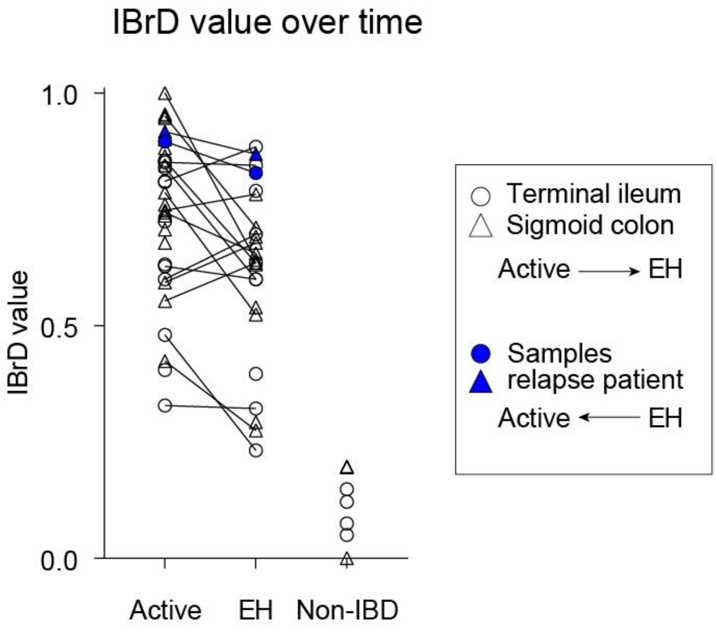

Figure S14. *Patient- and biopsy-specific differences of the IBrD over time (index patient, who experienced a relapse (T_F) within 10 weeks after discontinuing anti-TNF medication following EH (T_{BL}) is highlighted in blue). Active, n=33; EH, n=24; non-IBD controls, n=7. Related to Figure 5.*
