# Supplementary material for: Endoscopic healing in pediatric IBD perpetuates a persistent signature defined by Th17 cells with molecular and microbial drivers of disease
Source: Cell Rep Med. 2025 Jul 15;6(7):102236. doi: 10.1016/j.xcrm.2025.102236 (PMC12281438; doi:10.1016/j.xcrm.2025.102236)
Supplement: Document S2. Article plus supplemental information [file mmc12.pdf]

Cell Reports
Medicine

Article

Endoscopic healing in pediatric IBD perpetuates a persistent signature defined by Th17 cells with molecular and microbial drivers of disease

Graphical abstract

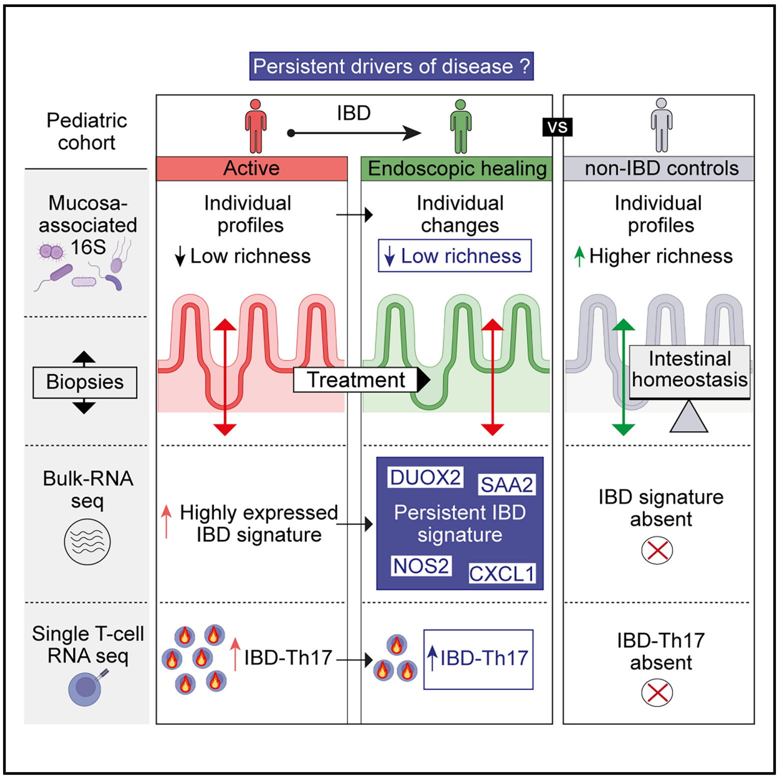

Highlights

- Multiple features with the potential to drive IBD persist despite endoscopic healing
- These changes vary among patients with IBD but are absent in non-IBD controls
- Residual IBD signature reflects persistently disturbed microbiome-host interaction
- Individual mucosal microbiota and TCRs can generate universal IBD mucosal response

Authors

Kolja Siebert, Tim Faro, Nikolai Köhler, ..., Dirk H. Busch, Dirk Haller, Tobias Schwerd

Correspondence

tschwerd@med.lmu.de

In brief

Persistent inflammation compromises long-lasting remission in patients with inflammatory bowel disease (IBD). Siebert et al. identify a transcriptomic signature of residual disease in intestinal biopsies from pediatric patients, even when endoscopic examinations suggest mucosal healing. These findings emphasize the need for more comprehensive healing beyond endoscopic appearance.

Siebert et al., 2025, Cell Reports Medicine *6*, 102236
July 15, 2025 © 2025 The Authors. Published by Elsevier Inc.
https://doi.org/10.1016/j.xcrm.2025.102236

CellPress

Cell Reports Medicine

CellPress
OPEN ACCESS

Article

Endoscopic healing in pediatric IBD perpetuates a persistent signature defined by Th17 cells with molecular and microbial drivers of disease

Kolja Siebert,[1] Tim Faro,[1,14] Nikolai Köhler,[2,14] Hannes Hölz,[1] Sebastian Jarosch,[3] Monica Matchado,[4] Deborah Häcker,[5,6] Federica De Zen,[1] Mohammad Samer Hajji,[1,15] Eberhard Lurz,[1] Sibylle Koletzko,[1,7] Josch K. Pauling,[2,8] Katja Steiger,[9] Klaus Neuhaus,[10] Caspar Ohnmacht,[11] Markus List,[4,12] Dirk H. Busch,[3] Dirk Haller,[5,6,13] and Tobias Schwerd[1,16,*]

[1]Department of Pediatrics, Dr. von Hauner Children's Hospital, University Hospital, LMU Munich, Munich, Germany
[2]LipiTUM, Chair of Experimental Bioinformatics, School of Life Sciences, Technical University of Munich, Freising, Germany
[3]Institute for Medical Microbiology, Immunology and Hygiene, Technical University of Munich, Munich, Germany
[4]Data Science in Systems Biology, School of Life Sciences, Technical University of Munich, Freising, Germany
[5]TUMCREATE, 1 CREATE Way, #10-02 CREATE Tower, Singapore 138602, Singapore
[6]Chair of Nutrition and Immunology, Technical University of Munich, Freising, Germany
[7]Department of Pediatrics, Gastroenterology and Nutrition, School of Medicine Collegium Medicum University of Warmia and Mazury, Olsztyn, Poland
[8]Computational Integrative Omics in Biomedicine, Institute for Clinical Chemistry and Laboratory Medicine, University Hospital and Faculty of Medicine Carl Gustav Carus, Dresden University of Technology, Dresden, Germany
[9]Institute of Pathology, TUM School of Medicine and Health, Technical University of Munich, Munich, Germany
[10]Core Facility Microbiome, ZIEL Institute for Food & Health, Technical University of Munich, Freising, Germany
[11]Center of Allergy and Environment (ZAUM), Technical University and Helmholtz Center Munich, Munich, Germany
[12]Munich Data Science Institute (MDSI), Technical University of Munich, 85748 Garching, Germany
[13]ZIEL Institute for Food and Health, Technical University of Munich, Freising, Germany
[14]These authors contributed equally
[15]Present address: Department of Pediatrics, Kinderklinik München Schwabing, Munich, Germany
[16]Lead contact
*Correspondence: tschwerd@med.lmu.de
https://doi.org/10.1016/j.xcrm.2025.102236

SUMMARY

Endoscopic healing (EH) is the major long-term treatment target for inflammatory bowel diseases (IBDs), mainly achieved by immune-suppressive therapies. However, the chronic and relapsing nature of the disease indicates a lifelong persistence of unknown tissue-associated IBD residues. Based on longitudinally collected gastrointestinal biopsies (n = 217) from pediatric patients with IBD (N = 32) and pediatric non-IBD controls (N = 5), we describe cellular, molecular, and microbial drivers of IBD that persist under EH in the terminal ileum and sigmoid colon. Whole biopsy transcriptomics in combination with single T cell analysis (72,026 cells) characterizes an inflammatory bowel residual disease (IBrD) signature, connecting stress- and inflammation-related tissue markers (e.g., *DUOX2*, *SAA2*, and *NOS2*) with pathogenic interleukin-17 (IL-17)-producing T helper cells. 16S rRNA gene sequencing reveals individual microbial composition with persistently low diversity, irrespective of disease location and activity. Overall, our study identifies a persisting IBD signature that reflects ongoing mucosal alterations despite EH. These markers may provide targets for future or sequential therapies.

INTRODUCTION

Inflammatory bowel diseases (IBDs) are chronic, lifelong disorders of the gastrointestinal tract caused by dysregulated interactions among the host, the intestinal microbiome, and further environmental factors.[1,2] The disease is mainly classified in one of its two major forms, Crohn's disease (CD) or ulcerative colitis (UC). Despite phenotypic differences, both share common features and treatment targets,[3–5] including mucosal healing on endoscopy (termed endoscopic healing [EH]). It is well established that clinical symptoms correlate poorly with endoscopic disease activity and that resolution of symptoms alone does not change IBD progression or cumulative bowel damage.[6] In contrast, EH, defined as the absence of ulcerations, can predict sustained clinical remission and resection-free survival.[7] It is of interest whether a more sustained remission, such as molecular remission, and the identification of EH-associated biomarkers provide additional benefits to patients with IBD.

Intestinal inflammation arises from dysregulated responses of tissue-resident immune cells, such as T cells, toward the intestinal microbiome. Microbial organisms and metabolites promote distinct T cell subsets with specific cytokine profiles and effector

© 2025 The Authors. Published by Elsevier Inc.
This is an open access article under the CC BY license (http://creativecommons.org/licenses/by/4.0/).

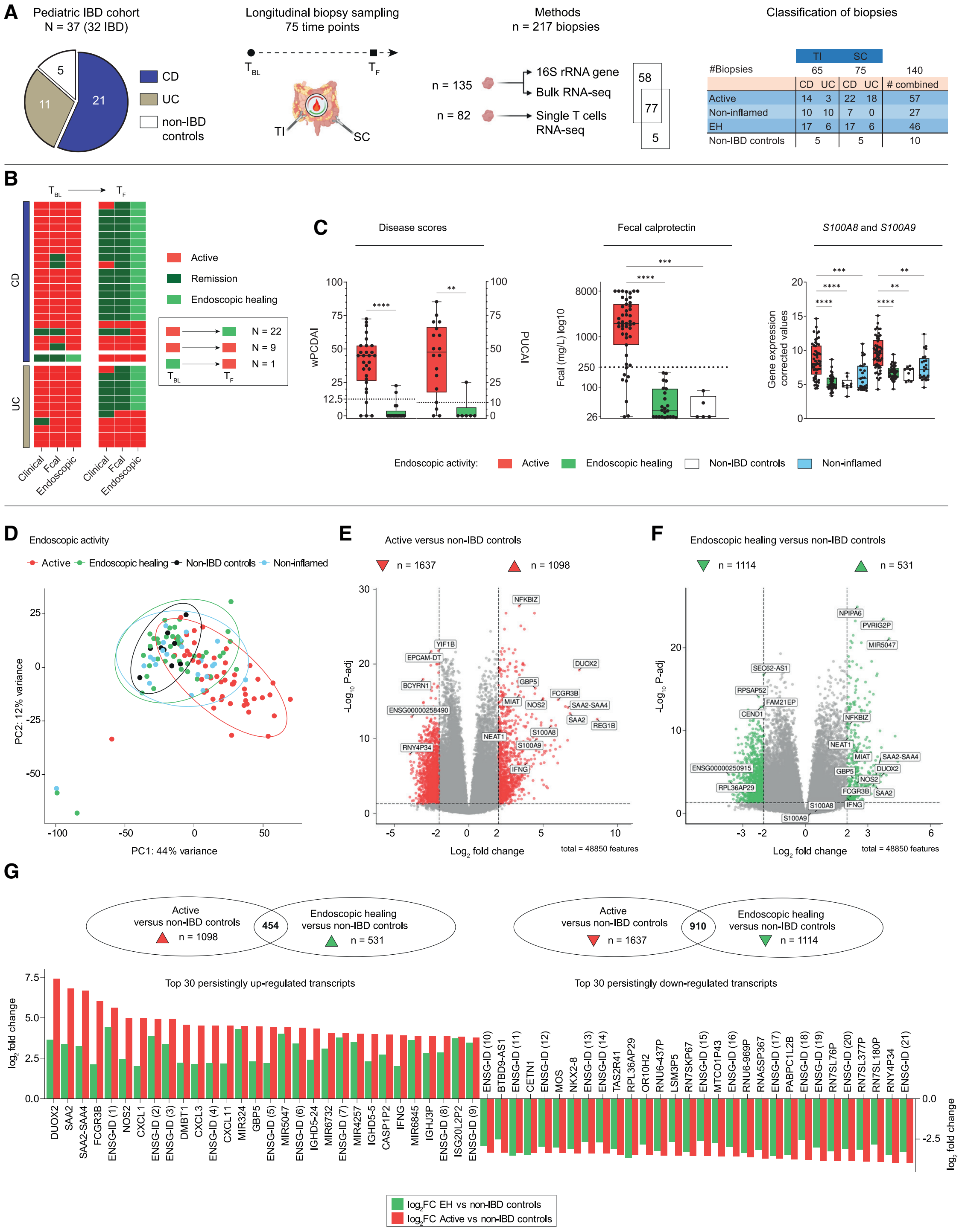

Figure 1. Cohort description and longitudinal tissue analysis

(A) Study overview (from left to right): patients of the pediatric IBD cohort and non-IBD controls. Paired biopsy sampling from 75 endoscopies (time point baseline [T_{BL}] = 32; time point follow-up [T_F] T_{F1} = 32, T_{F2} = 4, T_{F3} = 1, T_{F4} = 1; non-IBD controls = 5). In total, n = 217 biopsies from two sites (terminal ileum, TI; sigmoid colon, SC) were used for different methods: simultaneous isolation of bacterial DNA and host RNA (n = 135) for 16S rRNA gene amplicon sequencing (after quality control [QC], n = 135) and bulk RNA sequencing (after QC, n = 127) from single biopsies; single-cell RNA sequencing of live $CD45^+$, $CD3^+$ T cells (n = 82). Overview of biopsy classification according to IBD subtype (CD or UC), location (TI or SC), and endoscopic activity (active, non-inflamed [defined as "non-inflamed region in an active disease state"], endoscopic healing [EH], and non-IBD controls). The graphical representation of the intestine and biopsy samples was created with BioRender.com.

(legend continued on next page)

functions.[8] In addition to these environmental cues, T cell differentiation is shaped through their T cell receptors (TCRs), which discriminate pathogens from commensal intestinal microorganisms and, subsequently, influence T cell characteristics.[9] Interleukin-17 (IL-17)-producing T cells are an important subtype of $CD4^+$ T helper (Th) cells, responsible for homeostatic immune responses and conferring protection against bacterial and fungal infections.[10,11] However, in IBD, these cells become pathogenic and often produce additional cytokines, such as interferon (IFN)-γ or granulocyte-macrophage colony-stimulating factor (GM-CSF).[5,12] Over the last two decades, substantial efforts have been made to understand the upstream mechanisms that promote Th17 generation. These mechanisms include key cytokines, such as IL-23[13]; serum amyloid A (SAA) proteins[14]; and microbes adherent to the epithelium, such as *Citrobacter rodentium*, enterohemorrhagic *Escherichia coli O157:H7*, and segmented filamentous bacteria (SFB).[15,16] In this process, intestinal epithelial cells (IECs) act as a coordinating hub for the crosstalk between bacteria and immune cells.[17]

However, despite EH, loss of anti-inflammatory efficacy or treatment discontinuation often results in relapsing disease activity,[18,19] suggesting the persistence of molecular or microbial drivers of IBD. Therefore, EH does not equate to full disease resolution, and additional layers of healing are needed for prolonged remission. Analysis of residual tissue pathologies might help to identify persistent pathways, which are otherwise masked in the complexity of active inflammation. These pathways may be amenable to novel treatment strategies for maintaining long-term remission or could serve as biomarkers to determine the depth of remission. Here, we show that EH is associated with a persistent inflammatory bowel residual disease (IBrD), which is shared among patients despite individual mucosa-adherent microbes and patient-specific TCR repertoires.

RESULTS

IBD-associated features persist despite EH in pediatric IBD

We prospectively collected 217 biopsies from the terminal ileum (TI) and sigmoid colon (SC) of 32 pediatric patients with IBD (70 endoscopies) and 5 non-IBD controls (5 endoscopies). The median time between consecutive endoscopies was 38 weeks (range 3–107 weeks). Biopsies of patients with IBD were grouped according to endoscopic disease activity into active, EH, and non-inflamed (the non-inflamed region in an active disease state). Figure 1A gives an overview about the cohort, study design, methods, and biopsy samples, including origin and activity. Non-IBD controls matched patients with IBD in terms of age at endoscopy (12.6 ± 4.9 years), gender (2 females, 3 males), and biopsy sites (5/5 TI, 5/5 SC). Clinical details of patients with IBD are summarized in Table 1. At baseline (T_{BL}), 31/32 patients had active IBD on endoscopy (Figure 1B), involving the SC in all patients with UC and 16/21 patients with CD. Biopsies from the inflamed ileum were obtained from 11/21 patients with CD and 3/11 patients with UC (backwash ileitis) (Figure 1A). Among patients with active IBD, 22/31 patients (16 CD, 6 UC) achieved EH at the following endoscopy (T_{F1}) (Figure 1B), of whom one patient with CD reached EH at a subsequent endoscopy (T_{F2}). Ongoing endoscopic activity was found in 9/31 patients at follow-up endoscopies (T_{F1}; 3/9>T_{F1}). One patient with CD showed EH at T_{BL} and experienced disease exacerbation within 10 weeks after discontinuing anti-tumor necrosis factor (TNF) medication. Disease activity scores for IBD (weighted Pediatric CD Activity Index and Pediatric UC Activity Index, wPCDAI/PUCAI) and fecal calprotectin (Fcal) correlated with EH in the majority of patients (Figure 1B). EH in patients with IBD significantly improved disease activity as well as Fcal in stool and biopsy tissue (*S100A8* and *S100A9*) without further differences to non-IBD controls (Figure 1C).

Principal component (PC) analysis (PCA) of bulk transcriptomics data revealed the anatomical region as a major driver of separation by gene expression (IBD, $N = 30$; non-IBD controls, $N = 5$; samples, $n = 127$, Figure S1A). To establish molecular profiles across anatomical regions in IBD, we corrected for location and confirmed the absence of CD-, UC-, or treatment-specific clustering (Figure S1B). However, active samples partially separated from non-inflamed samples (Figures 1D, S1A, and S1B).

To identify differentially expressed genes (DEGs) that persist in whole tissue RNA of EH samples, we compared results from active vs. non-IBD controls and EH vs. non-IBD controls and looked for overlapping DEGs, defined as persistent genes (Figures 1E–1G). Active vs. non-IBD resulted in 1,098

(B) Heatmap showing different characteristics of IBD activity for each patient over time (from left to right): disease activity (wPCDAI and PUCAI), fecal calprotectin (Fcal), and endoscopic evaluation (Mayo endoscopic subscore or Simple Endoscopic Score for CD [SES-CD]). Dark green: remission by wPCDAI <12.5 points or PUCAI <10 points, Fcal <250 mg/L. Green: endoscopic healing defined as Mayo endoscopic subscore 0 or SES-CD ≤ 2 without ulcerations. Red: active IBD (not meeting remission criteria).

(C) Boxplots showing wPCDAI, PUCAI, Fcal, and gene expression levels of S100A8 and S100A9 according to endoscopic activity.

(D) Principal component analysis (PCA, after correction for location, batch, $n = 127$; patients with IBD, $N = 30$; non-IBD controls, $N = 5$) showing distribution of samples according to gene expression (top 500 most variable genes), labeled with endoscopic activity groups.

(E and F) Identifying up- and down-regulated genes in IBD compared to non-IBD controls. Volcano plots showing differentially expressed genes (DEGs; corrected for location, batch, age, gender; p.adj <0.05, $\log_2$ fold change ≥ 2.0 or ≤ -2.0) between (E) active ($n = 53$) vs. non-IBD controls ($n = 9$) and (F) EH ($n = 41$) versus non-IBD controls.

(G) Top 30 persistent DEGs shared between (E) and (F) as barplots, showing $\log_2$ fold changes of active (red) and EH (green) in comparison to non-IBD controls. These genes were significantly differentially expressed in active IBD compared to non-IBD controls and remained differentially expressed in EH compared to controls. CD, Crohn's disease; UC, ulcerative colitis.

Statistical tests: Mann-Whitney (p value) and Kruskal-Wallis (followed by comparison of all mean ranks and corrected by controlling the false discovery rate [FDR], Benjamini-Hochberg; p.adj value) with values indicated as * <0.05, ** <0.01, *** <0.001, and **** <0.0001. Boxplots showing the median, interquartile range, and the range from min to max. See also Figures S1–S6; Tables S1–S3.

Table 1. Baseline patient characteristics

		IBD (*N* = 32)	CD (*N* = 21)	–	UC (*N* = 11)
Gender	male	20 (63%)	14 (67%)	–	6 (55%)
	female	12 (37%)	7 (33%)	–	5 (45%)
Age at diagnosis (years)		11.3 (4.5)	11.8 (4.1)	–	10.1 (5.3)
Age at first endoscopy (years)		13.0 (3.5)	13.0 (3.1)	–	12.9 (4.3)
First degree family history of IBD		5 (16%)	3 (14%)	–	2 (18%)
Mean height (*Z* score)[a]		−0.6 (1.0)	−0.6 (1.0)	–	−0.6 (1.1)
Mean weight (*Z* score)[a]		−1.1 (1.4)	−1.0 (1.5)	–	−1.2 (1.1)
Mean body mass index (*Z* score)[a]		−1.0 (1.4)	−0.9 (1.6)	–	−1.1 (1.0)
Disease characteristics					
Location - lower GI[b]	none	–	0	proctitis	0
	ileum only	–	3 (14%)	left sided	0
	colon only (SC involvement)	–	5 (24%) (5/5)	extensive	4 (36%)
	ileocolonic (SC involvement)	–	13 (62%) (11/13)	pancolitis	7 (64%)
–	–	–	–	backwash ileitis	2 (18%)
Location - upper GI[b]	proximal (*n*, %)	–	18 (86%)	–	–
	distal (*n*, %)	–	4 (19%)	–	–
Behavior[b]	nonstricturing-nonpenetrating	–	19 (%)	never severe	6 (55%)
	stricturing	–	1 (5%)	ever severe	5 (45%)
	penetrating	–	0	–	–
	stricutring and penetrating	–	1 (5%)	–	–
Perianal involvement		–	2 (10%)	–	–
Evidence of growth delay		–	5 (24%)	–	–
Extraintestinal manifestations[c]		–	6 (29%)	–	1 (9%)
Previous surgery		–	2 (10%)	–	0
Disease activity					
Median wPCDAI		–	45 (32.5–55)	PUCAI	50 (30–70)
Inactive (wPCDAI <12.5)		–	2 (10%)	inactive (PUCAI <10)	1 (9%)
Mild (wPCDAI 12.5 to 40)		–	6 (29%)	mild (PUCAI 10 to 34)	2 (18%)
Moderate (wPCDAI 42.5 to 57.5)		–	9 (43%)	moderate (PUCAI 35 to 64)	4 (36%)
Severe (wPCDAI >57.5)		–	4 (19%)	severe (PUCAI ≥65)	4 (36%)
Baseline laboratory data					
Mean CRP (mg/dL)		3.0 (3.4)	3.4 (3.6)	–	2.3 (3.1)
Mean ESR (mm/h)		35.2 (22.5)	37 (22.3)	–	31.7 (23.6)
Elevated inflammatory marker[d]		26 (82%)	17 (81%)	–	9 (82%)
Mean albumin (g/dL)		4.1 (0.7)	4.1 (0.6)	–	4.1 (1.0)
Hypoalbuminemia (<3.5 g/dL)		4 (13%)	3 (14%)	–	1 (9%)
Mean hemoglobin, g/dL		11.5 (2.0)	11.7 (1.8)	–	11.1 (2.5)

(*Continued on next page*)

CellPress
OPEN ACCESS

Table 1. *Continued*

		IBD (*N* = 32)	CD (*N* = 21)	–	UC (*N* = 11)
Anemia (<10 g/dL)		6 (19%)	4 (19%)	–	2 (18%)
Median fecal calprotectin (mg/L) (IQR)		2,121 (881–4,307)	1,810 (557–4,334)	–	3,089 (1,591–4,397)
Treatment					
Newly diagnosed (<3 months)/treatment-naive		15 (47%)/13 (41%)	14 (67%)/12 (57%)	–	1 (9%)/1 (9%)
Treatment 1st endoscopy	EEN	–	3 (14%)	–	N/A
	5-ASA	–	2 (10%)	–	6 (55%)
	oral steroids	–	1 (5%)	–	6 (55%)
	topical steroids	–	1 (5%)	–	0
	thiopurines	–	0	–	2 (18%)
	methotrexate	–	3 (14%)	–	0
	anti-TNF	–	3 (14%)	–	3 (27%)
Treatment 2nd endoscopy	EEN	–	1 (5%)	–	N/A
	5-ASA	–	1 (5%)	–	8 (73%)
	oral steroids	–	0	–	1 (9%)
	thiopurines	–	0	–	2 (18%)
	methotrexate	–	9 (43%)	–	3 (27%)
	anti-TNF	–	12 (57%)	–	8 (73%)
	vedolizumab	–	1 (5%)	–	1 (9%)

Data are *N* (%), mean (SD), or median (IQR 25th to 75th percentile). 5-ASA, 5-aminosalicylic acids; BMI, body mass index; CRP, C-reactive protein; EEN, exclusive enteral nutrition; ESR, erythrocyte sedimentation rate; IBD, inflammatory bowel disease; IM, immunomodulator (e.g., azathioprine and methotrexate); PUCAI, Pediatric Ulcerative Colitis Activity Index; SC, sigmoid colon; TNF, tumor necrosis factor; wPCDAI, weighted Pediatric Crohn's Disease Activity Index.

[a] *Z* scores were calculated based on data of the German Health Interview and Examination Survey for Children and Adolescents (KiGGS).

[b] As per Paris classification.

[c] Three patients had joint manifestations, one had eye manifestations, six had skin manifestations, and one had autoimmune sclerosing cholangitis.

[d] CRP $\geq$ 0.5 mg/L or ESR $\geq$ 25 mm/h.

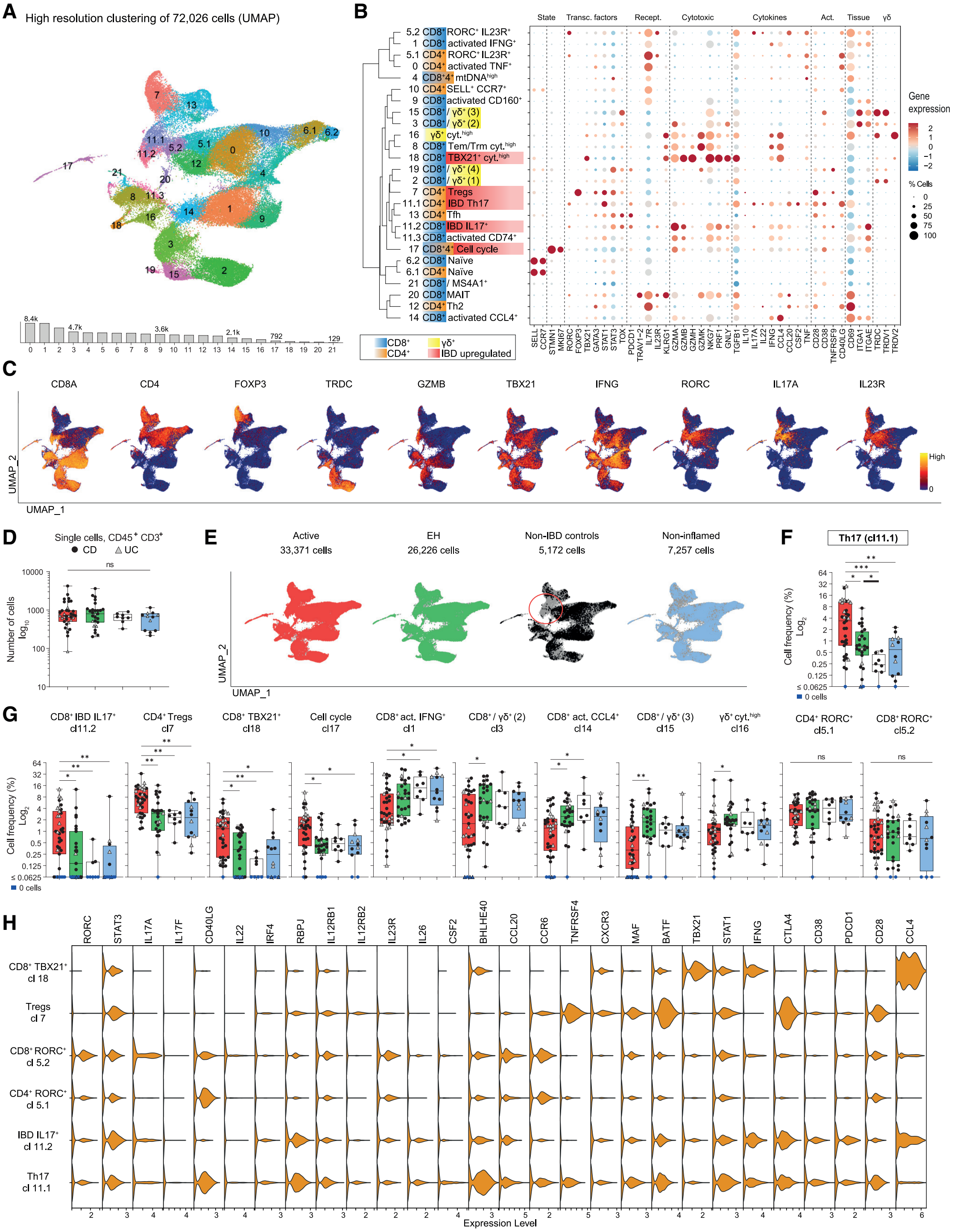

Figure 2. Single T cell analysis of paired IBD biopsies and non-IBD controls

(A) Uniform manifold approximation and projection (UMAP) showing high-resolution clustering of 72,026 live CD45+ CD3+ cells from 82 biopsies collected from 19 patients with IBD (CD, N = 14; UC N = 5) and 4 non-IBD controls. The bar graph below the UMAP shows total number of cells per cluster.

(B) Cluster tree with a dot plot highlighting gene expression of cluster-relevant genes (dimensions of circles represent the percentage of cells/cluster; color shows the average gene expression within this cluster, scaled in relationship to the other clusters).

(C) Feature plots showing selected gene expression of cells across the UMAP (from A).

(D) Number of single cells per biopsy (CD, black circles; UC, gray triangles) across endoscopic activity groups (active, n = 35; endoscopic healing (EH), n = 27; non-inflamed, n = 12; non-IBD controls, n = 8).

(legend continued on next page)

CellPress OPEN ACCESS

up-regulated and 1,637 down-regulated DEGs (log2 fold change (FC) ≥ 2, ≤ -2 and p.adj < 0.05, Figure 1E; Table S1). In EH, compared to non-IBD controls, 531 DEGs were up-regulated and 1,114 were down-regulated (Figure 1F; Table S2). Whereas *S100A8* (log2FC: 5.5) and *S100A9* (log2FC: 4.6) were up-regulated in active IBD, both transcripts returned to expression levels of non-IBD controls in EH. Identification of persisting gene transcripts in EH resulted in 454 up-regulated and 910 down-regulated genes (Figure 1G; Table S3). For 282 up-regulated and 556 down-regulated genes, we report the official gene symbols; otherwise, the Ensembl ID is provided (Figure 1G; Tables S1–S3). Genes, such as *DUOX2*, *NOS2*, *CXCL1*, CXCL3, *CXCL11*, *IFNG*, and *SAA2* and *SAA2-SAA4* from the SAA family, remained up-regulated in EH (Figures 1G and S2). Increased epithelial expression of DUOX2 and SAA in ileal and colonic EH samples was validated by immunohistochemistry (Figure S3), and *DUOX2*/*SAA2*/*NOS2*-expressing colonocytes were not detectable in single-cell data[20] of healthy controls compared to patients with IBD (Figure S4). Very limited overlap between site-specific DEGs of CD and UC samples and the 454 persistently up-regulated genes supports that these genes derive from both IBD subtypes and anatomical regions (Figure S5; Tables S4–S6). Moreover, associations between genes and functional pathways identified significant up-regulated pathway families that are mainly associated with inflammation and host-environmental interactions, such as the nuclear factor κB signaling pathway, which persisted in EH. In addition, down-regulated functions persisted in EH, such as oxidative phosphorylation (Figures S6A and S6B).

Classification of 72k single cells provided a high-resolution overview of intestinal T cells

To investigate IBD T cell signatures at high resolution, we analyzed 72,026 single, live CD45$^+$, CD3$^+$ cells from 82 biopsies, 19 patients with IBD (14 CD, 5 UC), and 4 non-IBD controls. In total, 33,371, 26,226, 7,257, and 5,172 cells were analyzed in active, EH, non-inflamed, and non-IBD control biopsies, respectively. Clustering cells according to transcriptomic profiles revealed 26 distinct clusters (cl). Among those, cl5, cl6, and cl11 were found to be divided in sub-clusters, respectively (Figures 2A and 2B, expression profiles; Table S7). Transcriptional profiles confirmed the presence of IBD-relevant genes and T cell subtypes, such as IL-17-producing or regulatory T cells (Figures 2B and 2C).

No separation was found according to IBD phenotype (CD, UC, and non-IBD controls), location (TI and SC), or endoscopic activity in a PCA (PC1 and PC2) plot based on T cell frequencies (Figure S7A). In addition, no IBD phenotype-, location-, or patient-specific T cell clusters were present (Figures S7B and S7C).

IBD-specific Th17 cells persist under EH

Mucosal T cell composition of patients showed individual dynamics over time (Figure S7D). To quantify the relative contribution of the different tissue-infiltrating T cell subsets, we calculated relative cell frequencies for each of the aforementioned cell cluster. No significant differences in cell numbers per biopsy were found (Figure 2D). Relative frequencies of ten T cell clusters differed significantly between IBD activity groups (active and EH) or non-IBD controls, but other clusters remained unchanged despite inflammation (Figures 2E–2G and S7E). Clusters with increased frequencies in active IBD (p.adj < 0.05) included IL-17-producing T cells (cl11.1 and 11.2), proliferating T cells (cl17), regulatory T cells (Tregs; cl7), and CD8$^+$ *TBX21*$^+$ cytotoxic T cells (cl18) (Figures 2F and 2G). Active IBD showed decreased frequencies for five clusters (p.adj < 0.05): CD8$^+$ $\gamma\delta^+$ tissue-resident intraepithelial lymphocytes (cl3, cl15, and cl16) and activated CD8$^+$ T cells (cl1 and cl14; Figure 2G). Only cl4, characterized by high levels of mitochondrial DNA, showed a clear CD-specific up-regulation compared to UC in active IBD (Figures S7E and S7F). With one exception, relative T cell frequencies returned to levels of non-IBD controls. However, Th17 cells (cl11.1) persisted at increased rates in EH tissue compared to non-IBD controls (p.adj < 0.05; Figure 2F). Notably, cl11.1 was almost completely absent in non-IBD controls (Figure 2E). In all non-IBD biopsies containing 5,172 single T cells, only 14 cells (0.27%) were part of this cluster. However, in IBD, 1,669 cells (5.00%) from active, 338 cells (1.29%) from EH, and 50 cells (0.69%) from non-inflamed biopsies were classified as Th17. Th17 cells from IBD biopsies expressed high levels of *RORC*, *IL23R*, *STAT3*, and *CSF2* (Figure 2H). A comparison to the other RORC$^+$, *IL23R*$^+$ T cell clusters without cell frequency alterations (cl5.1 and cl5.2) (Figure 2G) highlighted functionally relevant DEGs, such as *CSF2*, *CTLA4*, *RBPJ*, *IL26*, *BHLHE40*, and *IFNG* (Figure 2H). The expression profiles within a T cell cluster were, overall, similar between active IBD and EH. However, expression of *IL26* and *CSF2* was higher in Th17 cells derived from active tissue (Figure S8).

TCR analysis

To investigate the clonal distribution and expansion of T cells, we analyzed TCRs harboring an alpha and a beta chain (Figure 3A). TCR-expressing T cells (n = 48,273) were distributed across all clusters with higher expansion in CD8$^+$ T cells (i.e., cl1, cl9, and cl14; Figure 3B) and lowest appearance in CD8$^+$ $\gamma\delta^+$ T cells (i.e., cl15 and cl16) (Figure 3C). Coverage rates for single

(E) Distribution of cells highlighted for each activity group across the UMAP (from A). Numbers indicate available cells for the respective UMAP.

(F) Boxplot from T cell cluster 11.1 showing relative cell frequencies per cluster and biopsy (per individual biopsy: number of cells per cluster/total cell number per biopsy; CD, black circles; UC, gray triangles) according to endoscopic activity groups.

(G) Relative cell frequencies (as in F) of clusters with significant differences between activity groups and two RORC$^+$ clusters showing no significant differences between activity groups; statistical test used in (D, F, and G): Kruskal-Wallis test followed by comparing mean ranks between individual columns and multiple-testing correction using Benjamini-Hochberg, p.adj value indicated as * <0.05, ** <0.01, *** <0.001. Boxplots showing the median, interquartile range, and the range from min to max.

(H) Violin plots showing gene expression of selected clusters; CD, Crohn's disease; UC, ulcerative colitis; endoscopic activity groups (D–G): active, red; endoscopic healing, EH, green; non-IBD, white; non-inflamed (defined as "non-inflamed region in an active disease state"), light blue.

See also Figures S7 and S8; Table S7.

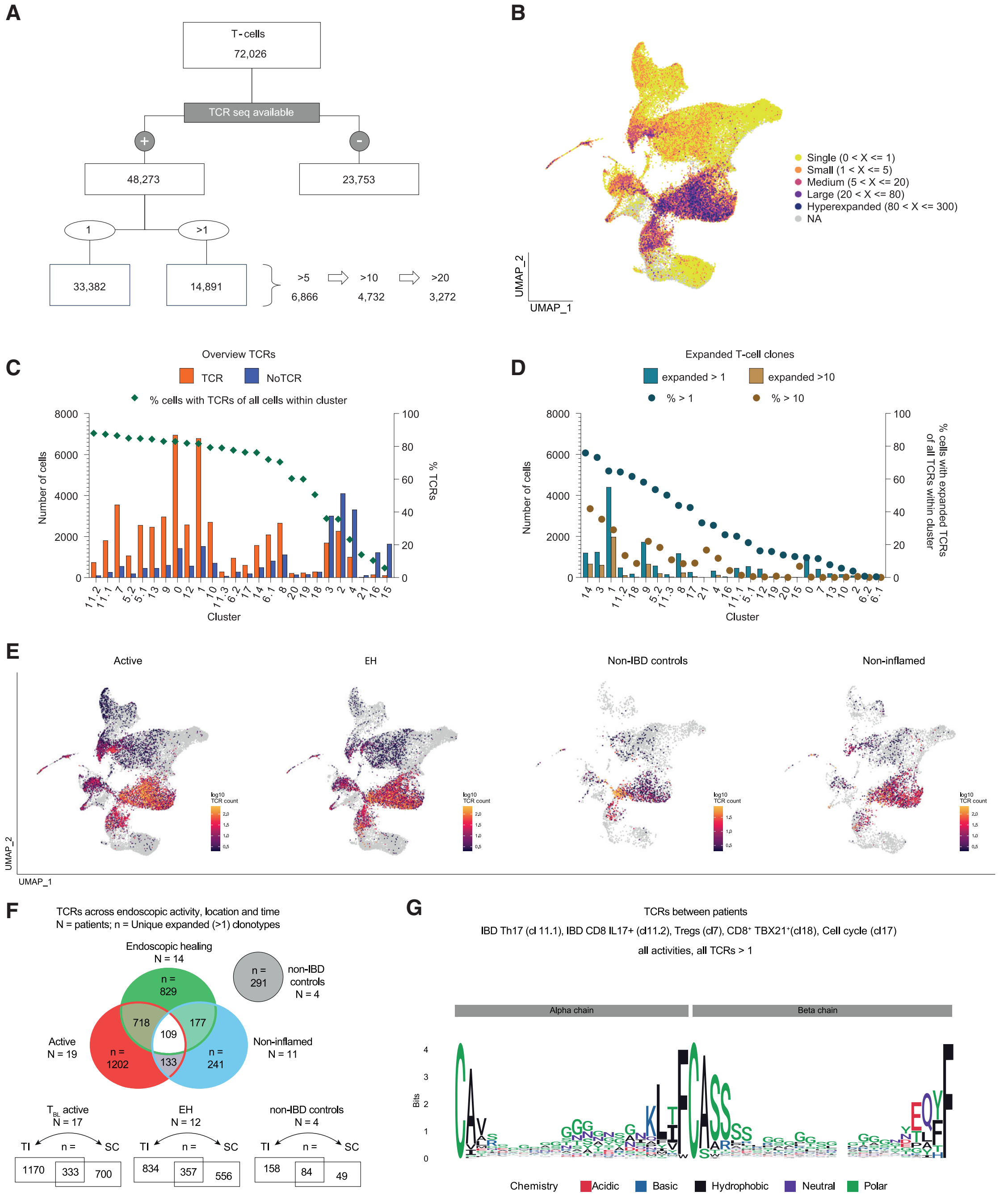

Figure 3. T cell receptor analysis of paired IBD biopsies and non-IBD controls

(A) Overview of single T cells (from Figure 2) with available T cell receptor (TCR) sequences (alpha and beta chains) and their clonal expansion (>1, >5, >10, and >20 clones).

(B) Distribution of single and expanded TCRs across the UMAP from Figure 2A. Color code indicates the level of expansion.

(C) Bar plot shows the number of cells with and without TCRs across single-cell clusters from Figure 2. Symbols depict percentages of TCR-expressing cells among total cluster cells.

(D) Bar plot as in (C), but showing expanded T cell clones (belonging to the group of >1 or >10 clones). Symbols show percentages of >1 and >10 expanded TCR-expressing cells of all TCR-expressing cells within this cluster, respectively.

(E) TCR-expressing clonotypes colored by level of expansion shown in UMAPs across endoscopic activity groups: active, endoscopic healing (EH), non-IBD and non-inflamed (defined as “non-inflamed region in an active disease state”).

(F) Venn diagrams giving an overview of unique expanded (>1) clonotypes (counted as one clonotype) shared across endoscopic activity groups, time, and location (N = number of patients, n = number of unique expanded clonotypes). Upper: shared clonotypes across endoscopic activity groups, including time

(legend continued on next page)

T cells with available TCR sequences differed between clusters, but Th17 (cl11) and Tregs (cl7) showed rates between 87% and 88%; Figure 3C). Six clusters had TCR coverage below 50% (cl2, cl3, cl4, cl15, cl16, and cl21).

Next, we examined clonal expansion based on a combination of complementarity-determining region 3 of both alpha and beta chain nucleotide sequences, defined as one clonotype. TCRs of 14,891 T cells were clonally expanded with two or more T cells sharing the same clonotype (Figure 3A). Absolute T cell expansion was found highest in activated $CD8^+$ $IFNG^+$ T cells (65% of cl1), which corresponded to 30% of all 14,891 clonally expanded T cells (Figure 3D). In contrast, among activated $CD4^+$ T cells (cl0), only 12% were clonally expanded. The $IL17^+$ T cells (cl11) showed a similar picture: among Th17 cells, 25%, and among $CD8^+$ $IL17^+$ T cells, 64% were clonally expanded. Of note, IBD Th17 cells showed the highest percentage of clonal expansion among all $CD4^+$ T cell clusters (Figure 3D).

Next, we looked at the distribution of clonally expanded T cells according to disease activity groups (Figure 3E). In all four groups, including non-IBD controls, clonal expansion was highest in activated $CD8^+$ T cells (i.e., cl1, cl9, and cl14). The five up-regulated clusters in active IBD (i.e., cl7, cl11.1, cl11.2, cl17, and cl18) were all hotspots for clonal expansion in active IBD, as well as EH (Figure 3E). Expanded TCRs appeared at both locations (TI and SC) and also persisted over time (from active/non-inflamed to EH; Figure 3F). In addition, expanded TCRs showed highly individual TCR sequences (Figure 3G).

Heterogeneous community profiles at the intestinal mucosa between pediatric patients with IBD

To identify mucosa-associated bacterial drivers, 16S rRNA gene amplicon sequencing was performed on 135 biopsy samples (31 patients with IBD, 5 non-IBD controls). Here, we used denoised amplicons, which each represent a molecular sequence to obtain operational taxonomic units (i.e., zero-radius operational taxonomic units [zOTU]). Richness, as indicated by the number of zOTUs, remained persistently low in IBD compared to non-IBD controls (Figure 4A). Longitudinal analysis revealed no specific zOTU clustering concerning IBD phenotype or disease activity (Figure 4B). Multi-dimensional scaling (MDS) of microbial profiles for the different IBD activity groups (active, $n = 56$; non-inflamed, $n = 26$; EH, $n = 43$) and non-IBD controls ($n = 10$) revealed significant differences but also intersections between groups: active vs. non-inflamed was indistinguishable (p.adj = 0.7, Figure 4C and S9A); active vs. EH (p.adj = 0.03), as well as active vs. non-IBD controls (p.adj = 0.03), showed significant differences; and EH vs. non-IBD controls was not significantly different or barely so (p.adj = 0.08, Figure 4C). In addition, samples from the TI showed no separation from the SC ($p = 1$, Figure S9B). Overall, the majority of zOTUs displayed a higher mean abundance in the non-IBD group (Figure 4D). Accordingly, differential analysis, focusing on significant differences of both active and EH versus non-IBD controls, only revealed zOTUs and genera, such as *Parabacteroides*, that were persistently low in abundance in IBD samples. *Clostridium sensu stricto 1*, which did not occur in non-IBD controls, showed a tendency of persistently higher abundance in IBD but decreased in most patients under EH. However, this genus was only present in some patients with IBD (Figure 4E). Time-series analysis of individual mucosa-associated bacteria showed highly patient-specific behavior (decreasing and increasing), resulting in diverse EH microbial community profiles (Figures 4F, 4G, and S9C). In addition, communities from same patients in both locations (TI and SC) clustered together, indicating a strong individual impact and more global rather than site-specific community changes (Figure 4G). Hence, these results align with the persistently low microbial richness observed and suggest the presence of permanently dysbiotic microbial communities, characterized by highly individual and dynamic profiles (Figures 4E–4G and S9D).

Subsequently, we looked for correlations between zOTUs and significant features from bulk and single-cell RNA sequencing. Despite the lack of disease-specific clustering (Figure 4B) and considerable overlap between CD and UC, a significant difference in the MDS analysis between CD and UC communities was found ($p = 0.005$, Figure S10A). Differential analysis between CD and UC identified 19 genera (Figure S10B). Correlation analysis for CD and UC showed diverse results, including few overlapping genera, such as *Bacteroides*, which correlated with most features across different zOTUs, and *Ruminococcus*, which correlated with *IFNG* in patients with CD (in addition to *NOS2*, *CXCL3*, and *CXCL1*) and UC (p value < 0.05, Figures 4H and 4I). In patients with UC, a zOTU of the genus *Escherichia* correlated with Th17 cells and *DUOX2*. However, only two correlations with a corresponding false discovery rate-corrected p value below 0.05 were identified, including one zOTU belonging to *Parabacteroides* and one belonging to *Bacteroides*, both correlating with *IFNG* in CD (Figure 4H). Overall, the results confirm a high degree of inter-individual variation, resulting in a variety of host-microbiota correlations.

Data integration reveals an IBrD signature in endoscopically uninflamed mucosa

Previous research demonstrated that transcriptionally based scores represent a sensitive measure of disease activity and persistent inflammation in macroscopically normal mucosa.[21,22] However, these scores involve several hundred DEGs ($n = 317$[21] and $n = 636$[22]) and showed very little separation between EH and non-IBD control samples (in contrast to active vs. EH, Figure 5A), limiting the potential deduction of mechanistic and clinically relevant insights in silently persistent disease. Therefore, we hypothesized that data integration of persistently up-regulated genes (Figure 1) and IBD-relevant T cell clusters (cl11.1, cl11.2, cl7, cl17, cl18, cl1, cl3, cl14, and cl15; Figure 2) could exploit inter-patient variability to discover distinct features of an IBrD signature. We used a bipartite correlation network between the modalities to identify DEGs associated with IBD-relevant T cell

(shared between active and EH, and between non-inflamed and EH) and location (shared between active and non-inflamed). Lower: shared clonotypes across locations (terminal ileum [TI], sigmoid colon [SC]) for active patients (including expanded clonotypes from active and non-inflamed samples) at T_{BL}, for EH (without the relapse patient and a patient with only SC available), and for non-IBD controls (from left to right).

(G) Sequence logo plot shows consensus of multiple sequence alignments of patient-specific TCR sequences from the five IBD-related T cell subtypes (from Figure 2: cl11.1, cl11.2, cl7, cl17, and cl18) at the level of amino acids.

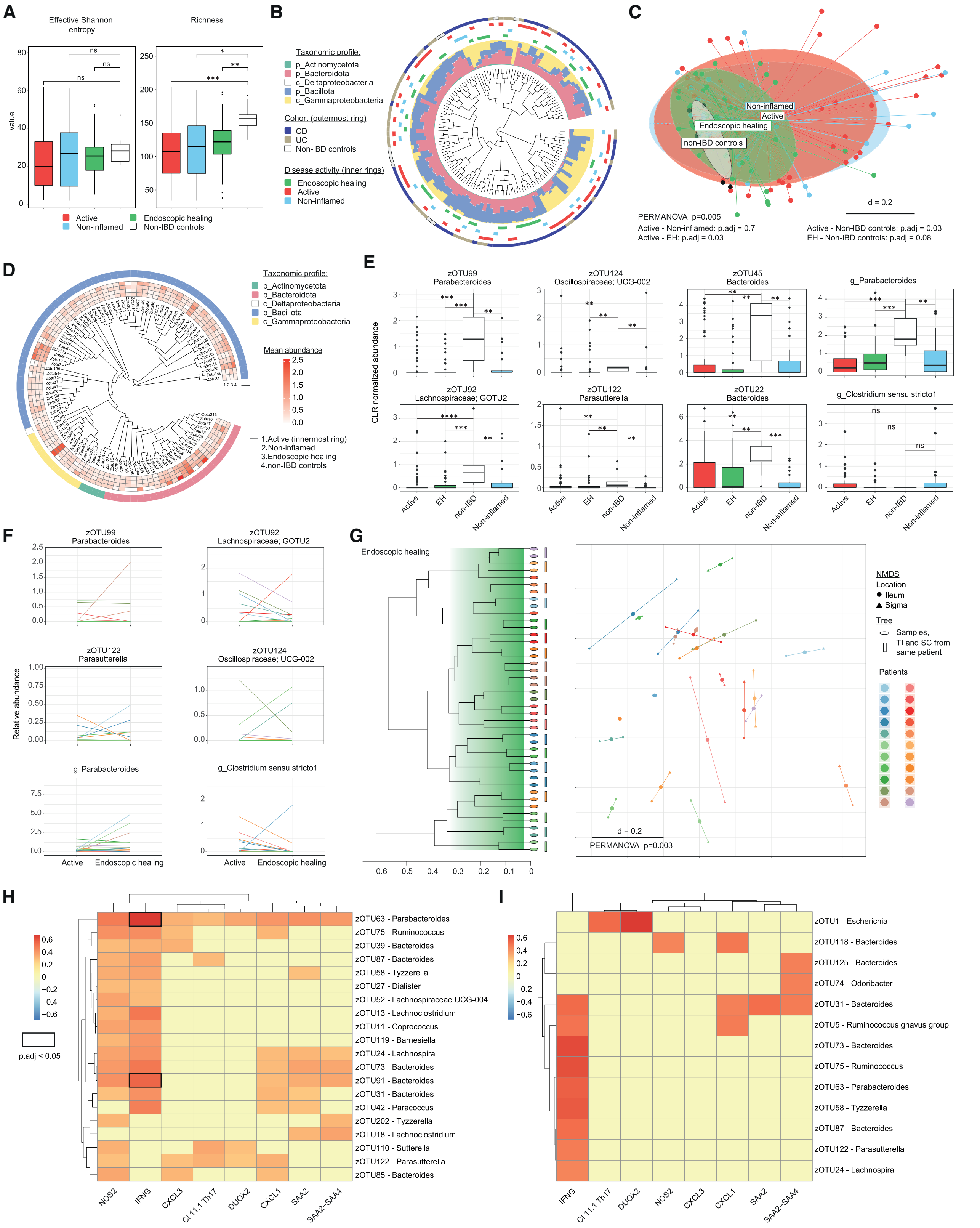

(legend on next page)

clusters, resulting in 81 of the initial 282 annotated and persistent DEGs (Figure 5B). Among those, DEGs in the network core were significantly correlated with surrounding T cell clusters. Transcripts such as *SAA2*, *DUOX2*, and *NOS2* showed strong positive association to Th17 cells (Figure S11). Importantly, our IBrD signature (81 DEGs, Table S8) improved the separation of active and EH mucosa from non-IBD control samples (Figure 5C), and signature genes were homogeneously expressed at low levels in all non-IBD controls (Figure S12). To control for covariates, we applied a linear mixed model (LMM) analysis, which reveals significant separation of active and EH samples from non-IBD samples (p.adj < 0.0001, p.adj = 0.003) but no separation by location or anti-TNF treatment (p.adj > 0.8). Excluding non-IBD controls, an additional LMM showed no differences between CD and UC (p.adj = 1) (Figure 5C).

Despite EH, only a few samples become more similar to non-IBD controls over time (indicated by arrows in the PCA, Figure 5C). To evaluate the degree of residual inflammation in EH, we quantified the IBrD signature and computed a trajectory through the space of the 14 first PCs (explaining 80% of the variance) and assigned a normalized pseudotime to each sample. The normalized pseudotimes reflect the extent of IBrD, where 0 corresponds to "no residual disease" and 1 to "active inflammation" (Figure 5D). Applying the IBrD signature to samples demonstrates a clear separation between IBD (mainly >0.5 for both active and EH) and non-IBD controls (<0.25) in biopsies with matched datasets (Figure 5E). Similar results were obtained in the total cohort of bulk RNA sequencing samples (n = 127, Figure S13). To confirm the robustness of the IBrD signature, we generated 200 PCAs, each based on 81 randomly selected genes taken from 3,016 DEGs that we found to be regulated by IBD (i.e., active and EH vs. non-IBD controls, Figure 1). There was a significant difference in the distances between the centroids of IBD groups and non-IBD controls in the PCA of the IBrD signature compared to PCAs derived from random gene selection (p value < 0.0001, Figures 5F and 5G). To corroborate our findings, we also tested the performance of the IBrD signature on an external dataset (GSE73661[23]) and found high discriminatory power between endoscopically healed mucosa (Mayo score 0) and non-IBD controls (Figures 5H–5J). Of note, the patient who experienced relapsing disease activity within 10 weeks after medication discontinuation (as indicated in Figure 1B) was found to have a high level of residual disease (Figure S14).

DISCUSSION

To briefly summarize, we analyzed 217 longitudinally collected biopsies from 32 pediatric patients with IBD and 5 non-IBD controls. We conducted high-resolution profiling and integrated network analysis of EH tissue combining single-cell transcriptomics of mucosal T cells with bulk RNA and microbial 16S rRNA amplicon sequencing data. Although 22 of 31 patients reached EH, our results demonstrate the persistence of cellular, molecular, and microbial drivers of inflammation, which is reflected in the IBrD and its pathognomonic signature of persistently up-regulated genes.

The IBrD signature is characterized by increased tissue expression of genes, such as *SAA2*, *NOS2*, *DUOX2*, *CXCL1*, *CXCL3*, *GBP5*, *DMBT1*, and *IFNG* and perpetuates persisting Th17 cells, as well as an associated perturbation of the mucosa-associated microbiota. Increased Th17 cell differentiation and pathogenic IL-17 responses to microbial stimulation are characteristic hallmarks of IBD.[24–26] In line with previous descriptions, the identified Th17 cells can be classified as pathogenic due to expression of pro-inflammatory genes for transcription factors (e.g., *RORC*, *TBX21*, *BHLHE40*, *STAT1*, *STAT3*, *PRDM1*, *IRF4*, and *BATF*), cytokines (e.g., *IL17A*, *IL22*,

Figure 4. Mucosa-associated microbiota by 16S rRNA gene amplicon analysis of paired biopsies and correlation heatmaps

(A) Alpha-diversity plotted as Shannon effective number and richness (number of zOTUs) across groups. Pairwise comparison between groups (Wilcoxon test, Benjamini-Hochberg corrected, p.adj values indicated as * <0.05, ** <0.01, *** <0.001).

(B) Beta-diversity of the cohort (patients with IBD, N = 31; non-IBD controls, N = 5; biopsies, n = 135) based on generalized UniFrac (gUniFrac) distances shown in a circular tree. Taxonomic profiles (relative abundance) are shown for each sample as color-coded stacked bar plots. Both classes, Delta- and Gammaproteobacteria, belong to the phylum Pseudomonadota (formerly known as Proteobacteria). Outer rings label samples according to IBD activity and phenotypes: active, red, n = 56; endoscopic healing, EH, green, n = 43; non-inflamed (defined as "non-inflamed region in an active disease state"), light blue, n = 26; non-IBD controls, white, n = 10; CD, dark blue, n = 82; UC, beige, n = 43.

(C) Beta-diversity of microbial profiles in a multi-dimensional scaling (MDS) plot showing disease groups. Comparison between pairs of groups: PERMANOVA with correction for multiple testing (Benjamini-Hochberg; p.adj values).

(D) Phylogenetic tree of top 100 (highest cumulative abundance) zOTUs. Mean abundances across endoscopic activities are shown in a heatmap (from inner to outer ring: active, non-inflamed, EH, and non-IBD controls) as well as the taxonomic group (outermost ring).

(E) Differential abundance analysis between IBD and non-IBD controls, focusing on the top zOTUs that showed differences in both active and EH compared to non-IBD controls. In addition, the top differentially abundant genus is shown and one of the scarce genera that was increased in IBD, but only in few patients. Wilcoxon test: the abundance of the taxa in each activity group is compared to the other groups (pairwise comparison between groups), multiple-testing correction using Benjamini-Hochberg, p.adj value indicated as * <0.05, ** <0.01, *** <0.001, and **** <0.0001. Boxplots showing the median, interquartile range, and whiskers extending to data points within 1.5 times interquartile range from the quartiles.

(F) Time-series analysis between active and EH of individual patients for zOTUs and genera from (E) (N = 22 patients). The mean relative abundance of the anatomical locations (TI and SC) was calculated for active and EH, respectively. For the patient with CD who first reached EH at T_{F2}, T_{BL} and T_{F2} are shown.

(G) Beta-diversity showing EH samples (n = 43) from both locations (TI and SC) from individual patients (N = 22) in a tree and non-metric multidimensional scaling (NMDS) based on gUniFrac distances. Samples are shown in different colors for each patient, respectively. Samples clustering together in the tree are highlighted with a bar in the patient-specific color.

(H and I) Correlation heatmaps between identified IBD-specific features (from bulk and single T cell RNA sequencing) and zOTUs of patients with CD (H) and UC (I). For CD, top 20/45 zOTUs are shown, which correlated to at least 2 IBD-specific features (H). The color code indicates Spearman's correlation coefficients among the respective T cell cluster, bulk genes, and zOTUs. All non-zero correlations have a corresponding p value <0.05 (corresponding FDR-corrected p values <0.05 are highlighted with a black frame).

See also Figures S9 and S10.

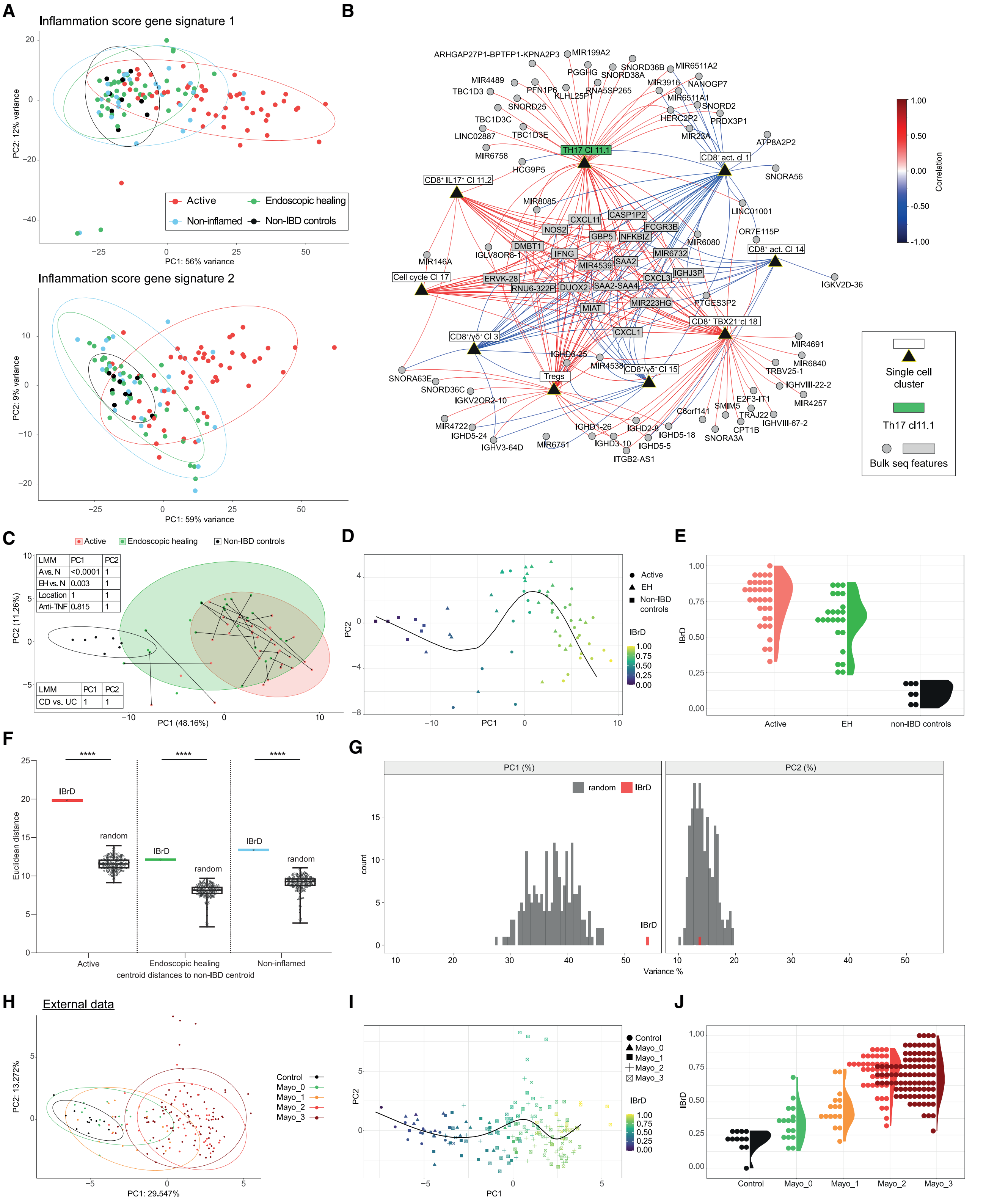

(legend on next page)

IL26, *IFNG*, *CSF2*, or *TNF*), and signaling receptors and regulators (e.g., *IL23R*, *CCR6*, *CD40L*, *CTLA4*, *CD28*, and *RBPJ*). Notably, Th17 cells were almost non-existing in non-IBD controls.

Development of pathogenic Th17 cells is mediated by a combination of the cytokines IL-6 and IL-23 and increased expression of SAA2,[13,27] through the molecular activity of multiple transcription factors. For instance, *IRF4* and *BATF* regulate chromatin accessibility and, in combination with *STAT3* and *RORC*, are key players for Th17 development.[28] Expression of *IL23R* can be regulated by the Notch signaling mediator *RBPJ*, which also represses anti-inflammatory IL-10 production within Th17 cells.[29] Interestingly, *RBPJ* was considerably less expressed in other *RORC*$^+$ and *IL23R*$^+$ T cells, which showed no differences in quantity between IBD and non-IBD controls. Following the cascade of IL23R, Blimp-1 (*PRDM1*) is one of the downstream elements of IL23R that acts together with RORγt (*RORC*) in activating Th17 and increasing the expression of *CSF2*, *IL17A/F*, and *IL23R*.[30] Cytokine expression of *IL17A*, *CSF2*, and *IFNG* is also regulated by *BHLHE40*, which can drive IFNG production independent of T-bet.[31,32] *CSF2* (GM-CSF) and *IL26* are the top two DEGs in our pathogenic Th17 cell cluster. GM-CSF is a key player of inflammatory responses and links pathogenic Th17 cells to the adaptive immune system and *IL23* expression.[33,34] *IL26* is a polyfunctional interleukin. It binds DNA, enhances innate immune responses, and kills extracellular bacteria by generating pores in the membrane.[35–37]

In contrast to the pathogenic Th17 cell cluster, all other T cell clusters returned to non-IBD levels in most biopsies, suggesting that environmental cues remain active in EH to promote pathogenic Th17 cell survival. The current paradigm is that Th17 development in the intestinal tract requires intestinal microbes.[15,16] Indeed, the transfer of human fecal IBD microbiota in mice exacerbated colitis and altered the balance of gut Th17 and RORγt$^+$ regulatory T cells.[38] Consistent with these findings, the mucosa-associated microbiota in our patients with IBD showed a patient-specific microbial composition, and the species richness was persistently reduced compared to non-IBD controls. However, at the mucosal level, we did not find a previously described dominance of IBD-associated Proteobacteria, which were found to be increased in fecal samples of patients with active IBD.[39] Nevertheless, despite highly individual sets of microbes being found, these might still result in a shared inflammatory tissue response perpetuating pathogenic Th17 cells. Similarly, colonizing mice with single bacteria, such as SFB, *Citrobacter rodentium* or enterohemorrhagic *Escherichia coli O157:H7*, showed that Th17 development is able to be mediated by phylogenetically diverse human gut microbes. In any case, it requires direct microbial interaction with IECs.[15,16,40] Interestingly, these strains are able to induce *SAA2*, *DUOX2*, and *NOS2* in IECs, which were among the top DEGs persistent in EH, linking intestinal microbes to pathogenic Th17 cells.[15] The direct contact of microbes with IECs induces epithelial SAA1/2 proteins, which promote differentiation and pathogenicity of Th17 cells.[41,42] In our study, *SAA2* is one of the top DEGs, which remained up-regulated in EH compared to non-IBD controls. More recently, Duan et al. provided more insight into how microbial epithelial adherence promotes Th17 cell differentiation. They showed that Th17 cell-inducing bacteria stimulate an endoplasmic reticulum stress response in IECs, which, in turn, promoted *Duoxa2/Duox2* expression, resulting in increased reactive oxygen species generation and Th17 induction via increased release of xanthine from IECs.[43] The antimicrobial DUOX2 is up-regulated in IBD and found together with an expansion of Proteobacteria in both treatment-naive pediatric UC and CD.[44] Of note, genetic *DUOX2* variants have been linked to increased plasma IL-17C concentrations.[45] NOS2, nitric oxide synthase 2, catalyzes the production of nitric oxide, a broad-spectrum anti-microbial agent, and NOS2 is found at increased levels in colonic IECs from patients with CD and UC.[17] The IBrD signature genes

Figure 5. Data integration of single-cell and bulk RNA sequencing identifies an IBrD

(A) Principal component analysis (PCA, batch and location corrected) of our bulk RNA sequencing data (*n* = 127) based on DEGs from two previously described inflammation scores/signatures tested on our data: (upper (1) 296 identified Ensembl IDs in our data from 317 possible gene symbols[21] and lower (2) 636 Ensembl IDs[22]).

(B) Correlation network of identified bulk and single T cell RNA sequencing features based on 64 samples from 18 patients with IBD and 4 non-IBD controls with matched datasets after data integration: nodes represent identified significant features (9 T cell clusters and 81 DEGs; derived from 282 annotated persisting up-regulated genes [172 from the 454 genes without an official gene symbol were excluded] from Figure 1G); edges represent significant (Spearman) correlations between features of the two modalities (red, positive; blue, negative); correlations between features of the same modality were not considered.

(C) PCA shows separation of IBD groups and non-IBD controls based on the inflammatory bowel residual disease signature: 81 significant DEGs from (B); samples: active, *n* = 33; EH, *n* = 24; non-IBD, *n* = 7. Arrows indicate patient-specific changes over time (only same locations for T_{BL} to T_F are connected). The following linear mixed model (LMM) was used to test for significant differences between IBD and non-IBD controls (A [active] vs. N [non-IBD controls] and EH vs. N) and to control for location or treatment-specific influences: PC $\sim$ (intercept) + disease status + anti-TNF + location + (1|patient). The LMM takes into account from which patient each sample originates; *p* values were Bonferroni corrected over all principal components and are shown in a table for PC1 and PC2. A similar LMM was performed to test for differences between CD and UC by including IBD phenotype and excluding non-IBD controls.

(D) PCA as in (C) with color code indicating IBrD scaled between 0 and 1, where 1 indicates highest activity.

(E) IBrD values (interval between 0 and 1) for each sample according to endoscopic activity.

(F) Boxplot of Euclidean distances between centroids of active, EH, and non-inflamed to the centroid of non-IBD controls calculated from the IBrD signature PCA (PC1 and PC2) (red) and from 200 PCAs derived from randomly selected genes (black, 81 genes taken from a pool of 3,016 IBD regulated genes). PCAs were always generated on 127 biopsy samples. Statistical test: one-sample Wilcoxon test, *p* value indicated as **** <0.0001. Boxplots showing the median, interquartile range, and the range from min to max.

(G) Explained variance (percent) of PC1 (left) and PC2 (right) of the PCAs from the IBrD signature (red) and from the 200 times randomly selected gene signatures (from F; dark gray).

(H–J) IBrD signature on an external dataset (GSE73661,[23] 26 genes were identified due to technical differences) following the pipeline as described in (C)–(E). See also Figures S11–S14.

also contained the chemokine CXC family members CXCL1, CXCL3, and CXCL11. These chemokines are enriched in mucosa of patients with CD and UC and are produced by macrophages, neutrophils, fibroblasts, and epithelial cells.[20,46] Their increased expression promotes the recruitment of neutrophils and other immune cells (e.g., activated T cells), potentially sustaining the inflammatory cycle in the intestinal mucosa.[47–49]

Our findings have several clinical implications. The majority of patients reached EH with anti-TNF therapy. However, our findings emphasize that while current IBD therapies suppress inflammatory pathways, they are unable to fully interrupt IBD-associated signaling cascades. One can speculate whether microbiome-modifying therapies targeting the mucosa-adherent pathobionts by, e.g., fecal microbiota transfer or using lytic bacteriophages, can attenuate the DUOX2-SAA-Th17 axis. In a mouse colitis model, the transfer of healthy donor-derived microbes reversed the inflammatory phenotype and reduced the proportion of mucosal Th17 cells.[50] Additional dietary factors might either directly or indirectly affect the intestinal microbiota or the host immune system.[51] Alternatively, the persistence of Th17 cells, which express IL23R and multiple pro-inflammatory cytokines, suggests the simultaneous or sequential use of several anti-cytokine agents, such as anti-TNF/anti-IL-23 in combination, or initial anti-TNF therapy to induce EH, followed by anti-IL23 for deeper remission. In contrast to anti-IL23, directly targeting IL-17 has, however, been clinically ineffective in CD and was associated with disease exacerbation and adverse events in some patients. This suggests that IL-17A per se might play a protective role in CD or in a subgroup of these patients, which may be mediated by other *IL17*-expressing clusters (e.g., cl 5.2).[52,53]

Strengths of this study include the representative pediatric IBD cohort and the longitudinal approach with homogeneous treatment. We identified a persisting signature that represents both IBD subtypes and both locations. Genes of our signature, including *DUOX2* and *NOS2*, were identified in epithelial cells of both the TI and the colon.[17] Despite these strengths, we identified several caveats of our study, which will need to be the focus of future research. First, our cohort is limited in size. The low number of samples may have limited the statistical power to detect more subtle or complex effects, and a larger cohort will potentially uncover additional molecular features that may have been missed in our study. In particular, in the highly individual 16S rRNA gene analysis, a larger sample size would be helpful to identify smaller effects. Future studies with larger, more homogeneous cohorts would be essential to validate our findings and to uncover additional molecular signatures. Despite this, our cohort is still representative of a pediatric IBD population with detailed clinical and endoscopic characterization. Stringent assessment of mucosal healing by endoscopy is supported by reductions in Fcal and change in the *S100A8* and *S100A9* transcripts that contribute to increases of the calprotectin protein. Despite low numbers of non-IBD controls, analysis of their tissues gave homogeneous results. Second, although we can link the persistence of pathogenic Th17 cells to up-regulated genes and disrupted bacterial communities, additional factors (e.g., metabolites) likely contribute and need to be identified. Third, we use 16S rRNA gene amplicon sequencing to characterize the mucosa-associated microbiota. Metagenomic sequencing was tested but not feasible due to the high abundance of human DNA in biopsy material. However, using a single endoscopic biopsy for analysis of host gene expression and adherent microbial composition, we aimed to detect residual microbial and molecular alterations in the closest possible proximity. Forth, we did not include follow-up of patients beyond EH. All patients in our clinic undergo proactive monitoring, including treatment adjustments, to prevent disease relapse. Our study reveals persistent cellular and molecular disease-associated features in patients achieving EH. We hypothesize that these features are merely suppressed by ongoing treatment rather than eliminated. Consequently, we propose that the degree of residual disease is more likely to determine the time to IBD recurrence after treatment withdrawal, rather than predicting disease relapse under an effective, ongoing treatment. In our cohort, almost all patients with EH exhibited ongoing molecular inflammation, as indicated by the IBrD signature, albeit to varying degrees. Therefore, measuring the depth of remission using the IBrD signature might help stratify patients who could potentially discontinue treatment versus those who should not (e.g., the relapse patient shown in Figure S14). However, prospective clinical trials are necessary to validate this approach.

In conclusion, our study provides evidence of an individual degree of residual disease in IBD despite EH, which can be determined through molecular profiling. Future research needs to show whether a refined treat-to-target approach based on treatment combinations or sequential medication administration adds further benefit to patients with IBD.

Limitations of the study

Our cohort provides longitudinal data on endoscopically healed mucosal tissue in a representative pediatric IBD population. While the IBrD signature was identified across patients, anatomical sites, and IBD subtypes (CD and UC), our analysis is based on single, rice-grain-sized biopsies intended to reflect the inflammatory status of the entire ileocolonic region. Adequate tissue sampling will be critical in future IBrD signature studies. The single-cell RNA sequencing of T cells increased the resolution of T cell subpopulations, but examining other cell clusters in our setup is crucial to identify additional persistent, IBD-associated cell types. Larger cohorts are needed to confirm our findings and potentially uncover additional molecular features, which may have been missed in our study. Increasing non-IBD controls may also provide new insights into IBD vs. non-IBD differences. Identifying microbial strains or environmental factors perpetuating the disturbed epithelial-immune-Th17 cell crosstalk could open new treatment options. As 16S rRNA gene amplicon sequencing limits microbiota analysis to genus/species level, identifying immunogenic microbes at the mucosal surface would require microbiota cultivation or novel methods of preprocessing for metagenomic sequencing to remove host DNA from endoscopic biopsies.

RESOURCE AVAILABILITY

Lead contact

Further information and requests should be directed to and will be fulfilled by the lead contact, Tobias Schwerd (tschwerd@med.lmu.de).

Materials availability

This study did not generate new, unique reagents.

Data and code availability

- Data that support the findings of this study are available in the manuscript and its supplementary materials. Bulk RNA sequencing data (raw feature count and corresponding metadata tables) are available at mediaTUM: https://doi.org/10.14459/2025mp1771962 and in Tables S9 and S10. Additional data are available on reasonable request from the lead contact. Raw sequencing data are protected and not publicly available due to pediatric patient privacy concerns. The requestor must describe the objectives of the research project and will need to sign a data access agreement (Article 26 EU GDPR "Joint controllers"). Data access will be considered for research purposes and non-commercial use only. In order to ensure patient privacy, access to personally identifiable information or sensitive clinical information will not be provided, and requests for data access must rigorously adhere to the consent agreements established with study participants.
- This paper does not report original code. Software and packages used for the analysis are provided in the key resources table. The code for the generation of the IBrD signature is provided at https://doi.org/10.14459/2025mp1771962.
- Any additional information required to reanalyze the data reported in this work paper is available from the lead contact upon request.

ACKNOWLEDGMENTS

We thank all our patients and their families for participation in this study. We thank Anja Jurk, Marie-Luise Frank, Simon Bühler, Katharina Socas, Till Siedenberg, and Maximilian Koch for assistance in biopsy collection. We also thank the comparative experimental pathology (CEP) and the tissue biobank (IBioTUM tissue, MRI, and TUM) for their immunohistochemistry (IHC) service.

This study was funded by the European Crohn's and Colitis Organisation (ECCO), the Deutsche Forschungsgemeinschaft (DFG; German Research Foundation) SFB1371/1-395357507 (projects P01 and P04), and the Leona M. and Harry B. Helmsley Charitable Trust (project number #2847). T.S. is supported by the Medical & Clinician Scientist Program (MCSP) of the Faculty of Medicine at LMU Munich. C.O. is supported by grants from the European Research Council (ERC Starting grant, project number 716718) and the DFG (project number OH 282/1-2 within FOR2599, project number 490846870–TRR355/1 TP05, and project number 395357507–CRC1371 TP07). Contributions by N.K. and J.K.P. were funded by the Bavarian State Ministry of Education and the Arts in the framework of the Bavarian Research Institute for Digital Transformation (bidt; grant LipiTUM).

AUTHOR CONTRIBUTIONS

K. Siebert and T.S. designed the study and experiments and wrote the first draft of the manuscript. K. Siebert generated the figures. K. Siebert, F.D.Z., H.H., and T.S. collected samples and extracted and analyzed clinical data. H.H. and T.S. recruited and characterized patients. T.S., M.S.H., and E.L. performed endoscopies and scored activity. M.M. and M.L. prepared RNA data and performed QC, which was analyzed by K. Siebert, T.F., M.M., and M.L. K. Steiger performed IHC and semi-quantification. K. Siebert, K. Steiger, and T.S. analyzed and evaluated the data. K. Siebert and S.J. prepared lamina propria mononuclear cells for single-cell analysis. S.J. performed the 10× pipeline. T.F. performed single-cell data integration, preparation, and QC. K. Siebert and T.F. analyzed single-cell and TCR data and, together with S.J., C.O., D.H.B., and T.S., evaluated and interpreted the results. K. Siebert, N. K., M.M., D. Häcker, K.N., and D. Haller performed, evaluated, and interpreted 16S rRNA gene amplicon analysis. N.K. integrated the 16S rRNA, bulk RNA, and single-cell RNA data and performed network analysis. N.K., J.K.P., M. L., K. Siebert., T.F., and T.S. evaluated the integrated data. K. Siebert, T.F., and N.K. performed validation of the IBrD. K. Siebert, T.F., N.K., and T.S. interpreted the final results. S.K. gave conceptual advice. All authors provided input on the manuscript, critically revised, and approved the final version of the manuscript.

DECLARATION OF INTERESTS

T.S. received lecture honoraria from Nutricia and MSD, personal honorarium as a member of an advisory board from AstraZeneca, and travel support from AbbVie and Ferring outside the submitted work. M.L. is a consultant for mbiomics GmbH. D. Haller served on the Microbiome Expert Panel from Reckitt Benckiser Health Limited. H.H. received a lecture honorarium from Nutricia outside the submitted work. K.N. received funding from HiPP, Pfaffenhofen, Germany, outside the submitted work. S.K. reports personal honorarium as a member of an advisory board or speaker's fee from AstraZeneca, AbbVie, Nestle Nutrition, Danone, Janssen, Pfizer, Sanofi, and Takeda outside the submitted work.

STAR★METHODS

Detailed methods are provided in the online version of this paper and include the following:

- KEY RESOURCES TABLE
- EXPERIMENTAL MODEL AND STUDY PARTICIPANT DETAILS
  - Patient cohort and study design
- METHOD DETAILS
  - Biopsy specimen collection and isolation of lamina propria mononuclear cells (LPMC)
  - Bulk tissue mRNA and mucosa-associated microbial DNA isolation
  - Immunohistochemistry (IHC)
  - IHC imaging
  - High-throughput 16S ribosomal RNA gene amplicon sequencing
  - Single cell sequencing
- QUANTIFICATION AND STATISTICAL ANALYSIS
  - RNA-seq analysis
  - IHC analysis
  - High-throughput 16S ribosomal RNA gene amplicon analysis
  - Single cell sequencing and analysis
  - Identification of a disease-associated correlation network
  - Inflammatory bowel residual disease (IBrD)
  - Linear mixed model (LMM)
- ADDITIONAL RESOURCES

SUPPLEMENTAL INFORMATION

Supplemental information can be found online at https://doi.org/10.1016/j.xcrm.2025.102236.

Received: April 15, 2024
Revised: December 3, 2024
Accepted: June 17, 2025
Published: July 15, 2025

REFERENCES

1. Kaser, A., Zeissig, S., and Blumberg, R.S. (2010). Inflammatory bowel disease. Annu. Rev. Immunol. *28*, 573–621.
2. MacDonald, T.T., Monteleone, I., Fantini, M.C., and Monteleone, G. (2011). Regulation of homeostasis and inflammation in the intestine. Gastroenterology *140*, 1768–1775.
3. Baumgart, D.C., and Sandborn, W.J. (2012). Crohn's disease. Lancet (London, England) *380*, 1590–1605.
4. Danese, S., and Fiocchi, C. (2011). Ulcerative colitis. N. Engl. J. Med. *365*, 1713–1725.
5. Schett, G., McInnes, I.B., and Neurath, M.F. (2021). Reframing Immune-Mediated Inflammatory Diseases through Signature Cytokine Hubs. N. Engl. J. Med. *385*, 628–639.
6. Turner, D., Ricciuto, A., Lewis, A., D'Amico, F., Dhaliwal, J., Griffiths, A.M., Bettenworth, D., Sandborn, W.J., Sands, B.E., Reinisch, W., et al. (2021). STRIDE-II: An Update on the Selecting Therapeutic Targets in

Inflammatory Bowel Disease (STRIDE) Initiative of the International Organization for the Study of IBD (IOIBD): Determining Therapeutic Goals for Treat-to-Target strategies in IBD. Gastroenterology *160*, 1570–1583.

7. Neurath, M.F., and Vieth, M. (2023). Different levels of healing in inflammatory bowel diseases: mucosal, histological, transmural, barrier and complete healing. Gut *72*, 2164–2183.
8. Arpaia, N., Campbell, C., Fan, X., Dikiy, S., van der Veeken, J., deRoos, P., Liu, H., Cross, J.R., Pfeffer, K., Coffer, P.J., and Rudensky, A.Y. (2013). Metabolites produced by commensal bacteria promote peripheral regulatory T-cell generation. Nature *504*, 451–455.
9. Snook, J.P., Kim, C., and Williams, M.A. (2018). TCR signal strength controls the differentiation of CD4+ effector and memory T cells. Sci. Immunol. *3*, eaas9103.
10. Korn, T., Bettelli, E., Oukka, M., and Kuchroo, V.K. (2009). IL-17 and Th17 Cells. Annu. Rev. Immunol. *27*, 485–517.
11. Harrington, L.E., Hatton, R.D., Mangan, P.R., Turner, H., Murphy, T.L., Murphy, K.M., and Weaver, C.T. (2005). Interleukin 17-producing CD4+ effector T cells develop via a lineage distinct from the T helper type 1 and 2 lineages. Nat. Immunol. *6*, 1123–1132.
12. Schnell, A., Littman, D.R., and Kuchroo, V.K. (2023). TH17 cell heterogeneity and its role in tissue inflammation. Nat. Immunol. *24*, 19–29.
13. Ahern, P.P., Schiering, C., Buonocore, S., McGeachy, M.J., Cua, D.J., Maloy, K.J., and Powrie, F. (2010). Interleukin-23 drives intestinal inflammation through direct activity on T cells. Immunity *33*, 279–288.
14. Lee, J.-Y., Hall, J.A., Kroehling, L., Wu, L., Najar, T., Nguyen, H.H., Lin, W.-Y., Yeung, S.T., Silva, H.M., Li, D., et al. (2020). Serum Amyloid A Proteins Induce Pathogenic Th17 Cells and Promote Inflammatory Disease. Cell *183*, 2036–2039.
15. Atarashi, K., Tanoue, T., Ando, M., Kamada, N., Nagano, Y., Narushima, S., Suda, W., Imaoka, A., Setoyama, H., Nagamori, T., et al. (2015). Th17 Cell Induction by Adhesion of Microbes to Intestinal Epithelial Cells. Cell *163*, 367–380.
16. Ivanov, I.I., Atarashi, K., Manel, N., Brodie, E.L., Shima, T., Karaoz, U., Wei, D., Goldfarb, K.C., Santee, C.A., Lynch, S.V., et al. (2009). Induction of intestinal Th17 cells by segmented filamentous bacteria. Cell *139*, 485–498.
17. Li, J., Simmons, A.J., Hawkins, C.V., Chiron, S., Ramirez-Solano, M.A., Tasneem, N., Kaur, H., Xu, Y., Revetta, F., Vega, P.N., et al. (2024). Identification and multimodal characterization of a specialized epithelial cell type associated with Crohn's disease. Nat. Commun. *15*, 7204.
18. Louis, E., Resche-Rigon, M., Laharie, D., Satsangi, J., Ding, N., Siegmund, B., D'Haens, G., Picon, L., Bossuyt, P., Vuitton, L., et al. (2023). Withdrawal of infliximab or concomitant immunosuppressant therapy in patients with Crohn's disease on combination therapy (SPARE): a multicentre, open-label, randomised controlled trial. The lancet. Lancet Gastroenterol. Hepatol. *8*, 215–227.
19. Buhl, S., Steenholdt, C., Brynskov, J., Christensen, K.R., Dorn-Rasmussen, M., Thomsen, O.Ø., Bendtzen, K., Klausen, T.W., Dahlerup, J.F., Thorsgaard, N., et al. (2022). Discontinuation of Infliximab Therapy in Patients with Crohn's Disease. NEJM Evid. *1*, EVIDoa2200061.
20. Garrido-Trigo, A., Corraliza, A.M., Veny, M., Dotti, I., Melón-Ardanaz, E., Rill, A., Crowell, H.L., Corbí, Á., Gudiño, V., Esteller, M., et al. (2023). Macrophage and neutrophil heterogeneity at single-cell spatial resolution in human inflammatory bowel disease. Nat. Commun. *14*, 4506.
21. Argmann, C., Hou, R., Ungaro, R.C., Irizar, H., Al-Taie, Z., Huang, R., Kosoy, R., Venkat, S., Song, W.-M., Di'Narzo, A.F., et al. (2023). Biopsy and blood-based molecular biomarker of inflammation in IBD. Gut *72*, 1271–1287.
22. Thomas, T., Rich-Griffin, C., Pohin, M., Friedrich, M., Aschenbrenner, D., Pakpoor, J., Jainarayanan, A., Voda, A., Sanches-Peres, R., Nee, E., et al. (2024). A longitudinal single-cell therapeutic atlas of anti-tumour necrosis factor treatment in inflammatory bowel disease. Nat. Immunol. *25*, 2152–2165.
23. Arijs, I., De Hertogh, G., Lemmens, B., van Lommel, L., de Bruyn, M., Vanhove, W., Cleynen, I., Machiels, K., Ferrante, M., Schuit, F., et al. (2018). Effect of vedolizumab (anti-α4β7-integrin) therapy on histological healing and mucosal gene expression in patients with UC. Gut *67*, 43–52.
24. Fujino, S., Andoh, A., Bamba, S., Ogawa, A., Hata, K., Araki, Y., Bamba, T., and Fujiyama, Y. (2003). Increased expression of interleukin 17 in inflammatory bowel disease. Gut *52*, 65–70.
25. Pène, J., Chevalier, S., Preisser, L., Vénéreau, E., Guilleux, M.-H., Ghannam, S., Molès, J.-P., Danger, Y., Ravon, E., Lesaux, S., et al. (2008). Chronically inflamed human tissues are infiltrated by highly differentiated Th17 lymphocytes. J. Immunol. (Baltimore, Md.: 1950) *180*, 7423–7430.
26. Kobayashi, T., Okamoto, S., Hisamatsu, T., Kamada, N., Chinen, H., Saito, R., Kitazume, M.T., Nakazawa, A., Sugita, A., Koganei, K., et al. (2008). IL23 differentially regulates the Th1/Th17 balance in ulcerative colitis and Crohn's disease. Gut *57*, 1682–1689.
27. Lee, Y., Awasthi, A., Yosef, N., Quintana, F.J., Xiao, S., Peters, A., Wu, C., Kleinewietfeld, M., Kunder, S., Hafler, D.A., et al. (2012). Induction and molecular signature of pathogenic TH17 cells. Nat. Immunol. *13*, 991–999.
28. Ciofani, M., Madar, A., Galan, C., Sellars, M., Mace, K., Pauli, F., Agarwal, A., Huang, W., Parkhurst, C.N., Muratet, M., et al. (2012). A validated regulatory network for Th17 cell specification. Cell *151*, 289–303.
29. Meyer Zu Horste, G., Wu, C., Wang, C., Cong, L., Pawlak, M., Lee, Y., Elyaman, W., Xiao, S., Regev, A., and Kuchroo, V.K. (2016). RBPJ Controls Development of Pathogenic Th17 Cells by Regulating IL-23 Receptor Expression. Cell Rep. *16*, 392–404.
30. Jain, R., Chen, Y., Kanno, Y., Joyce-Shaikh, B., Vahedi, G., Hirahara, K., Blumenschein, W.M., Sukumar, S., Haines, C.J., Sadekova, S., et al. (2016). Interleukin-23-Induced Transcription Factor Blimp-1 Promotes Pathogenicity of T Helper 17 Cells. Immunity *44*, 131–142.
31. Lin, C.-C., Bradstreet, T.R., Schwarzkopf, E.A., Jarjour, N.N., Chou, C., Archambault, A.S., Sim, J., Zinselmeyer, B.H., Carrero, J.A., Wu, G.F., et al. (2016). IL-1-induced Bhlhe40 identifies pathogenic T helper cells in a model of autoimmune neuroinflammation. J. Exp. Med. *213*, 251–271.
32. Piper, C., Zhou, V., Komorowski, R., Szabo, A., Vincent, B., Serody, J., Alegre, M.-L., Edelson, B.T., Taneja, R., and Drobyski, W.R. (2020). Pathogenic Bhlhe40+ GM-CSF+ CD4+ T cells promote indirect alloantigen presentation in the GI tract during GVHD. Blood *135*, 568–581.
33. Egea, L., Hirata, Y., and Kagnoff, M.F. (2010). GM-CSF: a role in immune and inflammatory reactions in the intestine. Expert Rev. Gastroenterol. Hepatol. *4*, 723–731.
34. Hamilton, J.A. (2008). Colony-stimulating factors in inflammation and autoimmunity. Nat. Rev. Immunol. *8*, 533–544.
35. Dambacher, J., Beigel, F., Zitzmann, K., De Toni, E.N., Göke, B., Diepolder, H.M., Auernhammer, C.J., and Brand, S. (2009). The role of the novel Th17 cytokine IL-26 in intestinal inflammation. Gut *58*, 1207–1217.
36. Meller, S., Di Domizio, J., Voo, K.S., Friedrich, H.C., Chamilos, G., Ganguly, D., Conrad, C., Gregorio, J., Le Roy, D., Roger, T., et al. (2015). T(H)17 cells promote microbial killing and innate immune sensing of DNA via interleukin 26. Nat. Immunol. *16*, 970–979.
37. Poli, C., Augusto, J.F., Dauvé, J., Adam, C., Preisser, L., Larochette, V., Pignon, P., Savina, A., Blanchard, S., Subra, J.F., et al. (2017). IL-26 Confers Proinflammatory Properties to Extracellular DNA. J. Immunol. (Baltimore, Md. : 1950) *198*, 3650–3661.
38. Britton, G.J., Contijoch, E.J., Mogno, I., Vennaro, O.H., Llewellyn, S.R., Ng, R., Li, Z., Mortha, A., Merad, M., Das, A., et al. (2019). Microbiotas from Humans with Inflammatory Bowel Disease Alter the Balance of Gut Th17 and RORγt+ Regulatory T Cells and Exacerbate Colitis in Mice. Immunity *50*, 212–224.e4.
39. Schirmer, M., Garner, A., Vlamakis, H., and Xavier, R.J. (2019). Microbial genes and pathways in inflammatory bowel disease. Nat. Rev. Microbiol. *17*, 497–511.
40. Geva-Zatorsky, N., Sefik, E., Kua, L., Pasman, L., Tan, T.G., Ortiz-Lopez, A., Yanortsang, T.B., Yang, L., Jupp, R., Mathis, D., et al. (2017). Mining the

Human Gut Microbiota for Immunomodulatory Organisms. Cell *168*, 928–943.e11.

41. Lee, J.-Y., Hall, J.A., Kroehling, L., Wu, L., Najar, T., Nguyen, H.H., Lin, W.-Y., Yeung, S.T., Silva, H.M., Li, D., et al. (2020). Serum Amyloid A Proteins Induce Pathogenic Th17 Cells and Promote Inflammatory Disease. Cell *180*, 79–91.e16.
42. Sano, T., Huang, W., Hall, J.A., Yang, Y., Chen, A., Gavzy, S.J., Lee, J.-Y., Ziel, J.W., Miraldi, E.R., Domingos, A.I., et al. (2015). An IL-23R/IL-22 Circuit Regulates Epithelial Serum Amyloid A to Promote Local Effector Th17 Responses. Cell *163*, 381–393.
43. Duan, J., Matute, J.D., Unger, L.W., Hanley, T., Schnell, A., Lin, X., Krupka, N., Griebel, P., Lambden, C., Sit, B., et al. (2023). Endoplasmic reticulum stress in the intestinal epithelium initiates purine metabolite synthesis and promotes Th17 cell differentiation in the gut. Immunity *56*, 1115–1131.e9.
44. Haberman, Y., Tickle, T.L., Dexheimer, P.J., Kim, M.-O., Tang, D., Karns, R., Baldassano, R.N., Noe, J.D., Rosh, J., Markowitz, J., et al. (2014). Pediatric Crohn disease patients exhibit specific ileal transcriptome and microbiome signature. J. Clin. Investig. *124*, 3617–3633.
45. Grasberger, H., Magis, A.T., Sheng, E., Conomos, M.P., Zhang, M., Garzotto, L.S., Hou, G., Bishu, S., Nagao-Kitamoto, H., El-Zaatari, M., et al. (2021). DUOX2 variants associate with preclinical disturbances in microbiota-immune homeostasis and increased inflammatory bowel disease risk. J. Clin. Investig. *131*, e141676.
46. Pavlidis, P., Tsakmaki, A., Pantazi, E., Li, K., Cozzetto, D., Digby-Bell, J., Yang, F., Lo, J.W., Alberts, E., Sa, A.C.C., et al. (2022). Interleukin-22 regulates neutrophil recruitment in ulcerative colitis and is associated with resistance to ustekinumab therapy. Nat. Commun. *13*, 5820.
47. Groom, J.R., and Luster, A.D. (2011). CXCR3 ligands: redundant, collaborative and antagonistic functions. Immunol. Cell Biol. *89*, 207–215.
48. Liu, Z., Chen, X., Wang, X., Chen, X., Song, C.H., Du, Y., Su, J., Yaseen, S. A., and Yang, P.C. (2011). Chemokine CXCL11 links microbial stimuli to intestinal inflammation. Clin. Exp. Immunol. *164*, 396–406.
49. Cole, K.E., Strick, C.A., Paradis, T.J., Ogborne, K.T., Loetscher, M., Gladue, R.P., Lin, W., Boyd, J.G., Moser, B., Wood, D.E., et al. (1998). Interferon-inducible T cell alpha chemoattractant (I-TAC): a novel non-ELR CXC chemokine with potent activity on activated T cells through selective high affinity binding to CXCR3. J. Exp. Med. *187*, 2009–2021.
50. Britton, G.J., Contijoch, E.J., Spindler, M.P., Aggarwala, V., Dogan, B., Bongers, G., San Mateo, L., Baltus, A., Das, A., Gevers, D., et al. (2020). Defined microbiota transplant restores Th17/RORγt+ regulatory T cell balance in mice colonized with inflammatory bowel disease microbiotas. Proc. Natl. Acad. Sci. USA *117*, 21536–21545.
51. Monteleone, I., Marafini, I., Dinallo, V., Di Fusco, D., Troncone, E., Zorzi, F., Laudisi, F., and Monteleone, G. (2017). Sodium chloride-enriched Diet Enhanced Inflammatory Cytokine Production and Exacerbated Experimental Colitis in Mice. J. Crohns Colitis *11*, 237–245.
52. Hueber, W., Sands, B.E., Lewitzky, S., Vandemeulebroecke, M., Reinisch, W., Higgins, P.D.R., Wehkamp, J., Feagan, B.G., Yao, M.D., Karczewski, M., et al. (2012). Secukinumab, a human anti-IL-17A monoclonal antibody, for moderate to severe Crohn's disease: unexpected results of a randomised, double-blind placebo-controlled trial. Gut *61*, 1693–1700.
53. Targan, S.R., Feagan, B., Vermeire, S., Panaccione, R., Melmed, G.Y., Landers, C., Li, D., Russell, C., Newmark, R., Zhang, N., et al. (2016). A Randomized, Double-Blind, Placebo-Controlled Phase 2 Study of Brodalumab in Patients With Moderate-to-Severe Crohn's Disease. Am. J. Gastroenterol. *111*, 1599–1607.
54. Reitmeier, S., Kiessling, S., Neuhaus, K., and Haller, D. (2020). Comparing Circadian Rhythmicity in the Human Gut Microbiome. STAR Protoc. *1*, 100148.
55. Dobin, A., Davis, C.A., Schlesinger, F., Drenkow, J., Zaleski, C., Jha, S., Batut, P., Chaisson, M., and Gingeras, T.R. (2013). STAR: ultrafast universal RNA-seq aligner. Bioinformatics (Oxford, England) *29*, 15–21.
56. Liao, Y., Smyth, G.K., and Shi, W. (2014). featureCounts: an efficient general purpose program for assigning sequence reads to genomic features. Bioinformatics (Oxford, England) *30*, 923–930.
57. Love, M.I., Huber, W., and Anders, S. (2014). Moderated estimation of fold change and dispersion for RNA-seq data with DESeq2. Genome Biol. *15*, 550.
58. Yu, G., Wang, L.-G., Han, Y., and He, Q.-Y. (2012). clusterProfiler: an R package for comparing biological themes among gene clusters. OMICS A J. Integr. Biol. *16*, 284–287.
59. Kevin Blighe (2018). EnhancedVolcano (Bioconductor).
60. Ritchie, M.E., Phipson, B., Wu, D., Hu, Y., Law, C.W., Shi, W., and Smyth, G.K. (2015). limma powers differential expression analyses for RNA-sequencing and microarray studies. Nucleic Acids Res. *43*, e47.
61. Lagkouvardos, I., Joseph, D., Kapfhammer, M., Giritli, S., Horn, M., Haller, D., and Clavel, T. (2016). IMNGS: A comprehensive open resource of processed 16S rRNA microbial profiles for ecology and diversity studies. Sci. Rep. *6*, 33721.
62. Quast, C., Pruesse, E., Yilmaz, P., Gerken, J., Schweer, T., Yarza, P., Peplies, J., and Glöckner, F.O. (2013). The SILVA ribosomal RNA gene database project: improved data processing and web-based tools. Nucleic Acids Res. *41*, D590–D596.
63. Dietrich, A., Matchado, M.S., Zwiebel, M., Ölke, B., Lauber, M., Lagkouvardos, I., Baumbach, J., Haller, D., Brandl, B., Skurk, T., et al. (2022). Namco: a microbiome explorer. Microb. Genom. *8*, mgen000852.
64. Lagkouvardos, I., Fischer, S., Kumar, N., and Clavel, T. (2017). Rhea: a transparent and modular R pipeline for microbial profiling based on 16S rRNA gene amplicons. PeerJ *5*, e2836.
65. Subramanian, B., Gao, S., Lercher, M.J., Hu, S., and Chen, W.-H. (2019). Evolview v3: a webserver for visualization, annotation, and management of phylogenetic trees. Nucleic Acids Res. *47*, W270–W275.
66. Butler, A., Hoffman, P., Smibert, P., Papalexi, E., and Satija, R. (2018). Integrating single-cell transcriptomic data across different conditions, technologies, and species. Nat. Biotechnol. *36*, 411–420.
67. Finak, G., McDavid, A., Yajima, M., Deng, J., Gersuk, V., Shalek, A.K., Slichter, C.K., Miller, H.W., McElrath, M.J., Prlic, M., et al. (2015). MAST: a flexible statistical framework for assessing transcriptional changes and characterizing heterogeneity in single-cell RNA sequencing data. Genome Biol. *16*, 278.
68. Borcherding, N., Bormann, N.L., and Kraus, G. (2020). scRepertoire: An R-based toolkit for single-cell immune receptor analysis. F1000Res. *9*, 47.
69. Street, K., Risso, D., Fletcher, R.B., Das, D., Ngai, J., Yosef, N., Purdom, E., and Dudoit, S. (2018). Slingshot: cell lineage and pseudotime inference for single-cell transcriptomics. BMC Genom. *19*, 477.
70. Turner, D., Ruemmele, F.M., Orlanski-Meyer, E., Griffiths, A.M., de Carpi, J.M., Bronsky, J., Veres, G., Aloi, M., Strisciuglio, C., Braegger, C.P., et al. (2018). Management of Paediatric Ulcerative Colitis, Part 1: Ambulatory Care-An Evidence-based Guideline From European Crohn's and Colitis Organization and European Society of Paediatric Gastroenterology, Hepatology and Nutrition. J. Pediatr. Gastroenterol. Nutr. *67*, 257–291.
71. van Rheenen, P.F., Aloi, M., Assa, A., Bronsky, J., Escher, J.C., Fagerberg, U.L., Gasparetto, M., Gerasimidis, K., Griffiths, A., Henderson, P., et al. (2020). The Medical Management of Paediatric Crohn's Disease: an ECCO-ESPGHAN Guideline Update. J. Crohn's Colitis *15*, jjaa161.
72. Jarosch, S., Köhlen, J., Ghimire, S., Orberg, E.T., Hammel, M., Gaag, D., Evert, M., Janssen, K.-P., Hiergeist, A., Gessner, A., et al. (2023). Multimodal immune cell phenotyping in GI biopsies reveals microbiome-related T cell modulations in human GvHD. Cell Rep. Med. *4*, 101125.
73. Schneider, V.A., Graves-Lindsay, T., Howe, K., Bouk, N., Chen, H.-C., Kitts, P.A., Murphy, T.D., Pruitt, K.D., Thibaud-Nissen, F., Albracht, D.,

et al. (2017). Evaluation of GRCh38 and de novo haploid genome assemblies demonstrates the enduring quality of the reference assembly. Genome Res. *27*, 849–864.

74. Jost, L. (2007). Partitioning diversity into independent alpha and beta components. Ecology *88*, 2427–2439.

75. Chen, J., Bittinger, K., Charlson, E.S., Hoffmann, C., Lewis, J., Wu, G.D., Collman, R.G., Bushman, F.D., and Li, H. (2012). Associating microbiome composition with environmental covariates using generalized UniFrac distances. Bioinformatics (Oxford, England) *28*, 2106–2113.

76. McGinnis, C.S., Patterson, D.M., Winkler, J., Conrad, D.N., Hein, M.Y., Srivastava, V., Hu, J.L., Murrow, L.M., Weissman, J.S., Werb, Z., et al. (2019). MULTI-seq: sample multiplexing for single-cell RNA sequencing using lipid-tagged indices. Nat. Methods *16*, 619–626.

77. Osorio, D., and Cai, J.J. (2021). Systematic determination of the mitochondrial proportion in human and mice tissues for single-cell RNA-sequencing data quality control. Bioinformatics (Oxford, England) *37*, 963–967.

78. Stuart, T., Butler, A., Hoffman, P., Hafemeister, C., Papalexi, E., Mauck, W. M., Hao, Y., Stoeckius, M., Smibert, P., and Satija, R. (2019). Comprehensive Integration of Single-Cell Data. Cell *177*, 1888–1902.e21.

79. Waltman, L., and van Eck, N.J. (2013). A smart local moving algorithm for large-scale modularity-based community detection. Eur. Phys. J. B *86*, 471.

STAR★METHODS

KEY RESOURCES TABLE

REAGENT or RESOURCE	SOURCE	IDENTIFIER
**Antibodies**		
Anti-human DUOX2	Merck	Cat#MaBN787
Anti-human SAA1/2	Novus Biologicals, Bio-Techne	Cat#NBP3-07766; RRID:AB_3555148
Anti-human CD45	DAKO	Cat#PB986
Anti-human CD3	Life Technologies, Invitrogen	Cat#17-0038-42; RRID:AB_10805861
TotalSeq™-C anti-human Hashtag antibodies (1–8)	BioLegend	Cat#394661; RRID:AB_2801031 - Cat#394675; RRID:AB_2820044
**Biological samples**		
Pediatric intestinal biopsy samples	This publication	DRKS00013306
**Chemicals, peptides, and recombinant proteins**		
RNAlater™	Invitrogen™	Cat#AM7020
RPMI 1640	Sigma Aldrich	Cat#R8758
Fetal Bovine Serum (FBS)	Gibco™	Cat#10500064
HBSS	Sigma Aldrich	Cat#H9394
HEPES	Cell Concepts	Cat#B-L3100-H
Collagenase IV	Sigma Aldrich	Cat#C5138
Dimethyl sulfoxide (DMSO)	Carl Roth	Cat#4720.4
β-Mercaptoethanol	Gibco™	Cat#21985023
Mutanolysin	Sigma Aldrich	Cat#M9901
Lysozyme	Carl Roth	Cat#8259.1
Propidium Iodide (PI)	Thermo Fisher	Cat#P1304MP
**Critical commercial assays**		
AllPrep DNA/RNA Mini Kit (50)	Qiagen	Cat#80204
NucleoSpin Tissue, Mini Kit	Macherey-Nagel	Cat#740952.50
Chromium Next GEM Single Cell 5′ Kit v2	10× Genomics	Cat#1000263
**Deposited data**		
IBD transcriptome dataset	Arijs et al.[23]	GSE73661
Single cell data	Garrido-Trigo et al.[20]	https://servidor2-ciberehd.upc.es/external/garrido/app/
Inflammation score 1	Argmann et al.[21]	bMIS_IBD
Inflammation score 2	Thomas et al.[22]	Inflammation score genes
Bulk RNA seq data (raw feature counts) and the code for the IBrD computations	This publication	mediaTUM: https://doi.org/10.14459/2025mp1771962; Tables S9 and S10
**Oligonucleotides**		
16S rRNA gene Illumina sequencing primers	Reitmeier et al.[54]	341F-ovh and 785r-ovh
**Software and algorithms**		
FastQC (v0.11.9)	Andrews S.	http://www.bioinformatics.babraham.ac.uk/projects/fastqc
Trim Galore (v0.6.6)	Krueger F.	https://www.bioinformatics.babraham.ac.uk/projects/trim_galore/
STAR (v2.7.9a)	Dobin et al.[55]	https://doi.org/10.1093/bioinformatics/bts635
featureCounts (v2.0.2)	Liao et al.[56]	https://doi.org/10.1093/bioinformatics/btt656

(*Continued on next page*)

Continued

REAGENT or RESOURCE	SOURCE	IDENTIFIER
DEseq2 (v1.42.0)	Love et al.[57]	https://doi.org/10.1186/s13059-014-0550-8
R (v4.3.2)	R Core Team	https://www.R-project.org
clusterprofiler (v4.10.0)	Yu et al.[58]	https://doi.org/10.1089/omi.2011.0118
EnhancedVolcano (v1.20.0)	Blighe[59]	https://doi.org/10.18129/B9.bioc.EnhancedVolcano
limma (v3.58.1)	Ritchie et al.[60]	https://doi.org/10.1093/nar/gkv007
IMNGS2 (date: 06.03.2024)	Lagkouvardos et al.[61]	https://www.imngs2.org
SILVA (v138)	Quast et al.[62]	https://doi.org/10.1093/nar/gks1219
Namco	Dietrich et al.[63]	https://exbio.wzw.tum.de/namco/
Rhea	Lagkouvardos et al.[64]	https://github.com/Lagkouvardos/Rhea
EvolView (date: 18.11.2024)	Subramanian et al.[65]	http://www.evolgenius.info/evolview/
cellranger (v6.0.2)	10× Genomics	https://support.10xgenomics.com/single-cell-gene-expression/software/pipelines/latest/installation
Seurat (v4.1.0)	Butler et al.[66]	https://satijalab.org/seurat/
MAST (v1.28.0)	Finak et al.[67]	https://doi.org/10.1186/s13059-015-0844-5
Prism (v10.1.1)	Graphpad	https://www.graphpad.com
scRepertoire (v1.11.0)	Borcherding et al.[68]	https://doi.org/10.12688/f1000research.22139.2
Slingshot (v2.4.0)	Street et al.[69]	https://doi.org/10.1186/s12864-018-4772-0

EXPERIMENTAL MODEL AND STUDY PARTICIPANT DETAILS

Patient cohort and study design

Pediatric patients aged 3–18 years, with suspected or established IBD were recruited to a mono-centric IBD cohort study (recruitment 2019–2022, Dr von Hauner Children's Hospital, LMU Munich) and followed prospectively (Female, $N = 12$; Male, $N = 20$; Western Europe, $N = 20$; Eastern Europe, $N = 7$; Asian, $N = 2$; Arab, $N = 2$; Other $N = 1$; Further details are provided in Table 1. Baseline patient characteristics). Written informed consent was obtained from parents/legal guardians and children older than 6 years signed a statement of assent (ethical approval LMU Munich, approval no. 17–801; German Clinical Trials Register accession no. DRKS00013306, date of registration 19.03.2018). Patients with an IBD-unclassified phenotype were excluded from our analysis. The diagnosis of IBD was made according to revised Porto criteria. Patients with confirmed IBD were treated according to current ESPGHAN/ECCO guidelines.[70,71] Patients without any evidence of inflammation after complete diagnostic work-up served as non-IBD controls. Patient assessment was performed at time of enrollment and at least every three months. Evaluation included medical history and physical examination, standard laboratory measures (including Fcal), calculation of the mathematically weighted Pediatric Crohn's Disease Activity Index (wPCDAI) for CD or the Pediatric Ulcerative Colitis Activity Index (PUCAI) for UC, and anthropometric data. PUCAI or wPCDAI less than 10 or 12.5, respectively, or fecal calprotectin <250 mg/L denoted remission. At baseline (T_{BL}), IBD work-up included an ileocolonoscopy, which was followed by ileocolonoscopy/sigmoidoscopy for evaluation of EH (T_F). Timing of re-evaluation endoscopy was based on clinical needs and/or recommendations by STRIDE-II guidelines (target window 6–12 months after T_{BL}). The goal of our study was to collect samples from patients that achieved EH after active IBD. Patients, who did not reach EH at T_{F1} were followed in $>T_{F1}$ endoscopies within 1 year for additional assessment of EH. We did not sample more than one EH time point from each patient. EH was defined according to STRIDE-II as Mayo endoscopic subscore of 0 and Simple Endoscopic Score for CD (SES-CD) ≤ 2 and absence of ulcerations.[6]

METHOD DETAILS

Biopsy specimen collection and isolation of lamina propria mononuclear cells (LPMC)

Single biopsy samples from terminal ileum and sigmoid colon were directly transferred into (1) RNAlater (Invitrogen; stored at −80°C after 24h incubation at 4°C) for bulk RNA sequencing and 16S rRNA gene amplicon sequencing, and (2) RPMI 1640 (Sigma Aldrich) + 10% FBS (Gibco) for single T cell RNA sequencing. For each site, biopsies for (1) and (2) were taken directly adjacent to each other with a small safety margin.

Biopsies from (2) were processed immediately after endoscopy to isolate lamina propria mononuclear cells (LPMCs) according to Jarosch et al.[72] with following adaptations: each biopsy was disrupted in tubes (gentleMACS C Tubes) containing 2 mL digestion medium (HBSS w/o Ca^{2+}, Mg^{2+} supplemented with HEPES 25 mM, FBS 2% and Collagenase IV 1 mg/mL) using a dissociator (Miltenyi Biotec: gentleMACS Octo Dissociator with Heaters) for 61 min at 37°C. Finally, after washing and passing the cell suspension through a 70 μm cell strainer using washing buffer (RPMI 1640 + 10% FBS +25 mM HEPES), cells were centrifuged (450×*g*), taken up in FBS with 10% DMSO, cooled down to −80°C, and then stored in liquid nitrogen until further processing.

Bulk tissue mRNA and mucosa-associated microbial DNA isolation

Microbial DNA and host RNA from single RNAlater preserved biopsy samples were isolated by combined mechanical tissue disruption with chemical and enzymatic cell lysis. Biopsies were transferred to a guanidine thiocyanate and β-mercaptoethanol solution and subsequently, mechanically disrupted for 1 min using the gentleMACS Octo Dissociator (same as used for LPMC isolation) with gentleMACS M Tubes. Remaining and intact bacterial cells were collected by centrifugation (14,000×*g*, 4 min). Supernatant DNA and RNA were isolated using the AllPrep DNA/RNA-isolation kit (Qiagen). The pellet was further processed in a second step using enzymatic lysis of bacterial cell walls through proteinase K, lysozyme and mutanolysin followed by isolation of the DNA (NucleoSpin Tissue, Macherey-Nagel). Chemical lysis (e.g., by detergents from kit-solutions) contributed to RNA/DNA isolation efficacy in both steps. The DNA from step one (in case bacteria were already mechanically disrupted) and step two (after enzymatic lysis) were finally combined and analyzed together. Our protocol resulted in high quality RNA with RNA integrity numbers RIN >7 and amounts of >300 ng/μL. However, 8/135 samples were excluded from sequencing or further analysis, either due to too low RNA concentration, poor RIN or low quality reads. DNA and RNA were stored at −80°C until sequencing. RNA-seq libraries (NEBNext Ultra II RNA Library Prep Kit; mRNA enrichment) were prepared for Illumina NovaSeq 6000 sequencing (2 × 150 paired end).

Immunohistochemistry (IHC)

For immunolabeling of target proteins, 2 μm sections of the paraffin embedded patient specimens were collected on superfrost plus slides (Thermo Scientific) and processed with the autostainer Bond RXm system (Leica, Wetzlar, Germany; all reagents from Leica) following the standard protocol provided by Leica. Briefly, sections were de-paraffinized with the Leica de-wax kit, then rehydrated by alcohol washes with decreasing concentrations (100%, 96%, 70%). Heat induced antigen retrieval with epitope retrieval solution 2 (that corresponds to EDTA buffer pH 8) for 30 min was followed by inactivation of endogenous peroxidase using 3% hydrogen peroxidase for 5 min. Monoclonal primary antibodies against *DUOX2* (mouse-*anti*-human; Merck, Darmstadt, Germany; MaBN787), diluted 1:100, or *SAA1/2* (mouse-*anti*-human; Bio-Techne, Wiesbaden, Germany, NBP-07766-100 μg), diluted 1:50, were applied for 15 min at room temperature. The polymer refine detection kit without post primary reagent detected the antibody during 8 min incubation and diaminobenzidine turn-over into dark brown precipitate served as visualization of target-protein presence (DAB refine detection kit protocol with total duration of 10 min). Counterstaining was performed with the included hematoxyline kit (10 min incubation) before dehydration of the samples with washes of increasing alcohol concentrations (70%, 96%, 100%) and xylene. Stained samples were then mounted with Pertex mounting medium (Histolab, Goeteborg, Sweden; 00801).

IHC imaging

Stained samples were imaged using the Aperio AT2 scanner (Leica) at 40× magnification. Representative images were taken at 20× magnification.

High-throughput 16S ribosomal RNA gene amplicon sequencing

Sequencing libraries for 16S ribosomal RNA (rRNA) gene amplicon sequencing were prepared in a two-step PCR as previously described.[54] In brief, for 16S ribosomal RNA (rRNA) gene amplicon sequencing, V3-V4 regions were amplified in a first PCR (primer 341f, 5′-CCT ACG GGN GGC WGC AG-3′, and 785r, 5′-GAC TAC HVG GGT ATC TAA TCC-3'; both with overhangs). The second PCR targets the overhangs and adds adapters to generate Illumina libraries. Fifteen cycles for each PCR were used. A MiSeq (Illumina) with v3 cartridges (PE300) was used for amplicon sequencing.

Single cell sequencing

Isolated LPMCs were prepared in two batches for fluorescence-activated cell sorting (live $CD45^{+}CD3^{+}$ cells; BD FACSAria III) and single cell analysis. Thawed LPMC suspensions were stained on ice with anti-CD45 (murine anti-CD45 PB450, clone T29/33, DAKO, #PB986), anti-CD3 (murine anti-CD3 APC, clone UCHT1, Life Technologies, #17-0038-42), individual hashtag oligos (HTOs; TotalSeq anti-human Hashtag antibodies) and propidium iodide (PI). $CD45^{+}CD3^{+}PI^{-}$ cells were sorted and pooled based on different HTOs. We aimed for 5000–7000 cells per biopsy. The 10× Genomics protocol (Chromium Next GEM Single Cell 5′ Reagent KitsKit, v2 Chemistry Dual Index) was followed as previously described[72] for scRNA sequencing (20,000 reads/cell for gene expression libraries and 5,000 reads/cell for TCR libraries; Illumina 2 × 150 paired-end sequencing on a NovaSeq 6000).

QUANTIFICATION AND STATISTICAL ANALYSIS

Each biopsy was treated as an individual data point. Unless otherwise stated, "N = " represents the number of patients and "n = " the number of biopsies. Boxplots show the median and whiskers the range from min to max (Figure 1 and scRNA frequency results) or 1.5 times interquartile range (Bulk RNA and 16S rRNA gene sequencing). For analysis and the generation of plots, GraphPad Prism Software v10.1.1 for Windows, Namco (v1.1) and R v4.3.2 were used. *p*-Values <0.05 were defined as significant with * <0.05, ** <0.01, *** <0.001, and **** <0.0001. Benjamini-Hochberg was used for correction unless stated otherwise. Detailed explanations of statistical analysis are provided below for each section and in the figure legends.

RNA-seq analysis

Quality assessment of raw reads was carried out using FastQC (v0.11.9), followed by Trim Galore (v0.6.6) to remove low-quality reads. The remaining high-quality reads were aligned to the human reference genome GRCh38 using STAR (v2.7.9a)[55,73] and quantified using featureCounts (v2.0.2).[56] Genes with a count lower than 5 were removed. DEseq2 (v1.42.0[57]) was used to run variance stabilizing transformation and to identify differentially expressed genes (DEGs) in pairwise comparisons (corrected for batch, location, age at endoscopy [groups: <6, 6–12, and >12] and gender). DEGs were selected based on an absolute log2FC ≥ 2, ≤ -2 and p. adj <0.05 (Benjamini-Hochberg corrected). Gene set enrichment analysis (GSEA) was performed using the R (v4.3.2) package clusterprofiler (v4.10.0).[58] The Wald statistic computed by DESeq2 was used as ranking metric for the GSEA. Volcano plots were created using the R package EnhancedVolcano (v1.20.0).[59] To visualize the batch effect corrected data, we used the function *removeBatchEffect* from the limma package (v3.58.1) in R.[60]

IHC analysis

Results were evaluated by a blinded pathologist using Aperio ImageScope (v12.4.6.5003), who scored expression intensity (0–3, for absent, weak, moderate or strong) and the percentage of positive cells. The values were evaluated separately for the luminal epithelium and the intestinal crypts.

High-throughput 16S ribosomal RNA gene amplicon analysis

FASTQ files were analyzed using IMNGS2 beta (https://www.imngs2.org/, last date accessed 06.03.2024), which is an improved version of IMNGS.[61] SILVA v138[62] was used for taxonomic assignment. To mitigate the amount of spurious zOTUs, all zOTUs with relative abundance below 0.25% across all samples and prevalence $\leq$10% were removed. Downstream analysis was performed with Namco (v1.1, last access date April 2025)[63] and Rhea.[64] zOTU tables were subjected to centered log-ratio transformation to account for compositionality. Taxa richness and Shannon effective number of zOTUs were used to describe alpha-diversity[74] and generalized UniFrac distances for beta-diversity.[75] Relative abundance was used to show taxonomic profiles. The circular tree (Figure 4B) was visualized using EvolView (www.evolgenius.info/evolview/, last date accessed: April 2025).[65]

Single cell sequencing and analysis

Annotations were generated via cellranger v6.0.2 (10× Genomics) against the human reference genome GRCh38 (GENCODEv32 with Ensembl 98) and the associated VDJ reference genome. For scRNA analysis, we followed the Seurat vignette using Seurat v4.1.0[66] and filtered as follows: features that occurred in <3 cells and cells with <200 features were removed. We normalized the RNA data with log normalization, then calculated and scaled the 2000 most variable genes, which were later used for PCA and visualization. Centered log-ratio transformation was applied to the HTO assay data and then demultiplexed using *MULTIseqDemux*, Seurats implementation of the method MULTI-seq.[76] To ensure high quality data, we filtered for 10% mtDNA,[77] removed cells which had more than 4000 detected genes and those that were annotated as 'negative' or 'doublet' by the demultiplexing algorithm.

Since our data consisted of two separate runs, we performed data integration using reciprocal PCA (RPCA) for dimensionality reduction and then applied the anchor-based approach[78] implemented in the Seurat package. We identified clusters using the Louvain algorithm[79] implemented in the *FindClusters* Seurat method with a resolution of 0.7 (on the first 15 dimensions), based on a shared nearest neighbor graph (determined by the *FindNeighbors* method). Clusters were consecutively numbered according to quantity of cells, starting with highest amount in cluster 0 (8380 cells) to lowest number of cells in cluster 21 (129 cells). Sub-clustering of cluster 5, 6 and 11 was performed using *FindNeighbors* and *FindClusters* with appropriate resolution to split individual filtered clusters into two (cluster 5 and 6) or three (cluster 11) sub-clusters. The tree was generated using the *BuildClusterTree* function. Annotation of clusters was based on known key markers of T cell subtypes and significant differences in gene expression. To determine DEGs between clusters (p.adj <0.05; average-log2FC), we used the *FindAllMarkers* function (Seurat) and the MAST test v1.28.0[67] to incorporate confounding variables ("Location", "Batch", "Disease phenotype") during the DEG testing.

For visualization and calculation, we used Seurat v4.1.0 in R v4.3.2. Boxplots were generated with GraphPad Prism Software v10.1.1 for Windows and significance calculated by Kruskal-Wallis test followed by comparing mean ranks between individual columns; p.adj-values <0.05 after correction for multiple testing were considered significant (Benjamini-Hochberg).

In addition to scRNA analysis, we performed TCR repertoire analysis of TCRs composed of alpha and beta chains. TCRs with a missing alpha or beta chain were removed from analysis and we discarded any additional chains. The combination of complementarity determining region 3 (CDR3) nucleotide sequences from alpha and beta chains were defined as one clonotype. Expanded T cell

CellPress
OPEN ACCESS

clones were determined by the number of cells expressing a specific clonotype. In addition to the Seurat pipeline, we used scRepertoire v1.11.0 to visualize the distribution of TCRs across activity groups and clusters.[68]

Identification of a disease-associated correlation network

Pairwise Spearman's correlation coefficients between single-cell clusters and bulk transcripts were computed. All coefficients, for which the corresponding Benjamini-Hochberg corrected *p*-values were >0.05, were set to zero. The resulting correlation matrix was used to generate the weighted adjacency matrix for the correlation network.

Inflammatory bowel residual disease (IBrD)

Based on the features extracted by the correlation network (Figure 5B), a Principal Component Analysis (PCA) was conducted. Subsequently, the number of Principal Components (PCs) explaining 80% of the variance was computed and the respective number of PCs were used as input for slingshot (v2.4.0)[69] to compute a trajectory through the space of PCs and assign a pseudotime to each sample. We define the inflammatory bowel residual disease (IBrD) values as the pseudotimes normalized to the interval [0,1], where "1" represents high molecular activity and "0" no residual disease. To compute the IBrD signature values on the whole cohort (bulk transcriptome; $n = 127$), all samples were scaled and centered with the mean and variance of the samples, which were initially used for the feature selection (Figure 5C). Subsequently, we used the rotation matrix of the PCA from the original scoring to compute the coordinates on the PCs for all new samples. We then projected the new samples onto the original slingshot trajectory using the *project_to_curve* function from the princurve package (v2.1.6). The resulting pseudotime values are again normalized by the min/max values from the original samples to obtain the final IBrD values.

Linear mixed model (LMM)

In Figure 5C (PCA) the following linear mixed model (LMM) was used to test for significant differences between IBD and non-IBD controls and to control for location or treatment-specific influences, while also taking into account from which patient each sample originates:

$$\text{PC} \sim (\text{intercept}) + \text{DiseaseStatus} + \text{AntiTNF} + \text{Location} + (1|\text{Patient})$$

where (1|Patient) indicates that patients were included as random effects, while disease status, location and anti-TNF treatment were included as fixed effects. *P*-Values were computed with t-tests and Bonferroni-corrected over all principal components. A similar LMM was performed to test for differences between CD and UC by including IBD phenotype (CD, UC) without non-IBD controls:

$$\text{PC} \sim (\text{intercept}) + \text{DiseasePhenotype} + \text{AntiTNF} + \text{Location} + (1|\text{Patient}).$$

ADDITIONAL RESOURCES

Our study is registered at the German Clinical Trials Register accession no. DRKS00013306, date registration 19.03.2018.

Cell Reports Medicine, Volume *6*

Supplemental information

Endoscopic healing in pediatric IBD perpetuates a persistent signature defined by Th17 cells with molecular and microbial drivers of disease

Kolja Siebert, Tim Faro, Nikolai Köhler, Hannes Hölz, Sebastian Jarosch, Monica Matchado, Deborah Häcker, Federica De Zen, Mohammad Samer Hajji, Eberhard Lurz, Sibylle Koletzko, Josch K. Pauling, Katja Steiger, Klaus Neuhaus, Caspar Ohnmacht, Markus List, Dirk H. Busch, Dirk Haller, and Tobias Schwerd

Supplemental information

Table of Contents

Figure S1-2: Bulk RNA sequencing, related to Figure 1

Figure S3: Immunohistochemistry, related to Figure 1

Figure S4: External single cell data, related to Figure 1

Figure S5-6: Bulk RNA sequencing, related to Figure 1

Figure S7-8: Single T-cell RNA sequencing, related to Figure 2

Figure S9-10: 16S rRNA genes of mucosa associated microbiota, related to Figure 4

Figure S11-14: IBrD signature, related to Figure 5

Figure S1

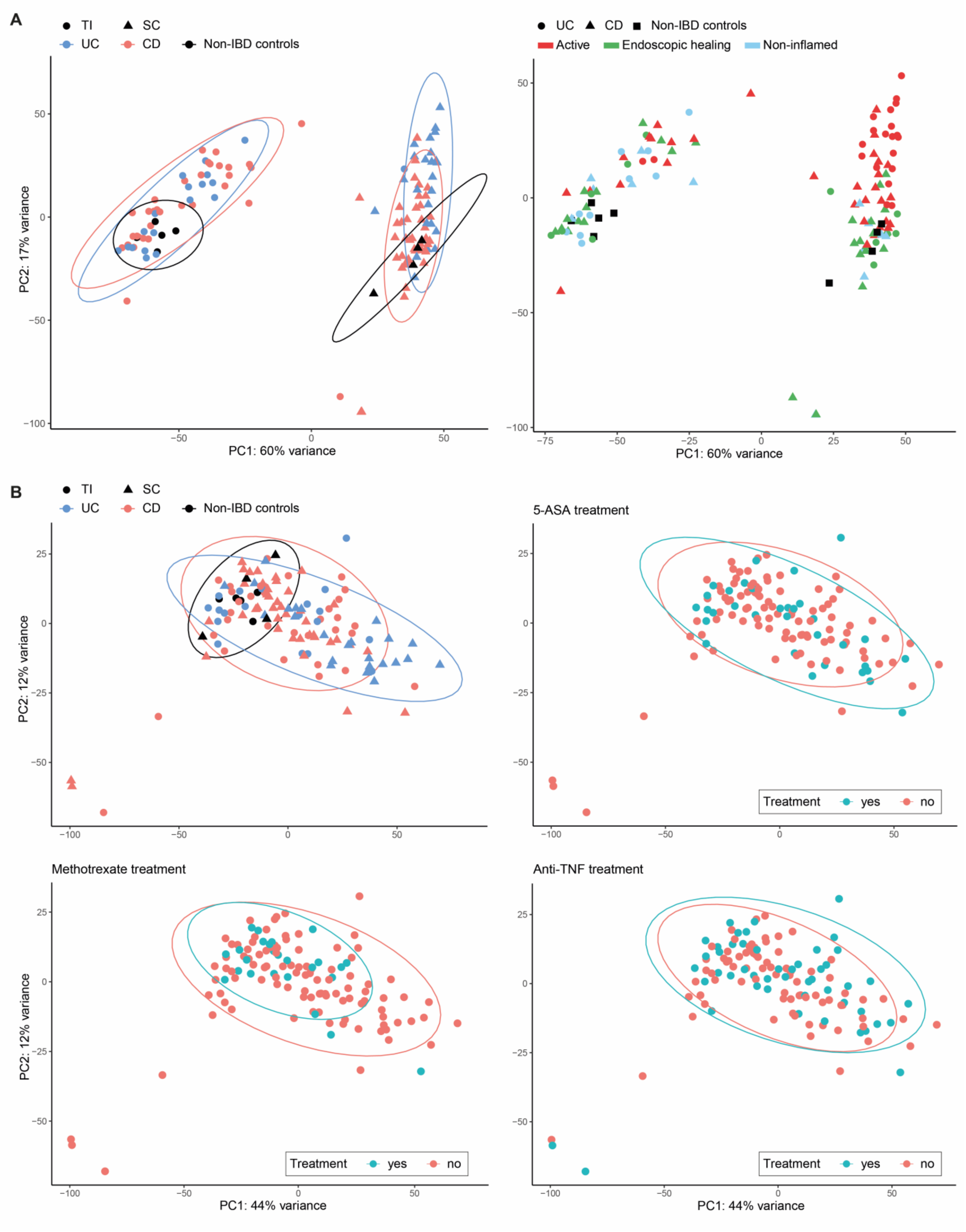

Figure S1. ***(A)*** *Principal component analysis (PCA, batch corrected) showing distribution of bulk RNA sequencing samples (samples n = 127; IBD patients, N = 30; non-IBD controls, N = 5) with (left) location (terminal ileum (TI) and sigmoid colon (SC)) and (right) endoscopic activity highlighted. Disease phenotype is shown in both.* ***(B)*** *PCA (after correction for location and batch), labeled with CD, UC and non-IBD controls, location and most common treatments. Related to Figure 1.*

Figure S2

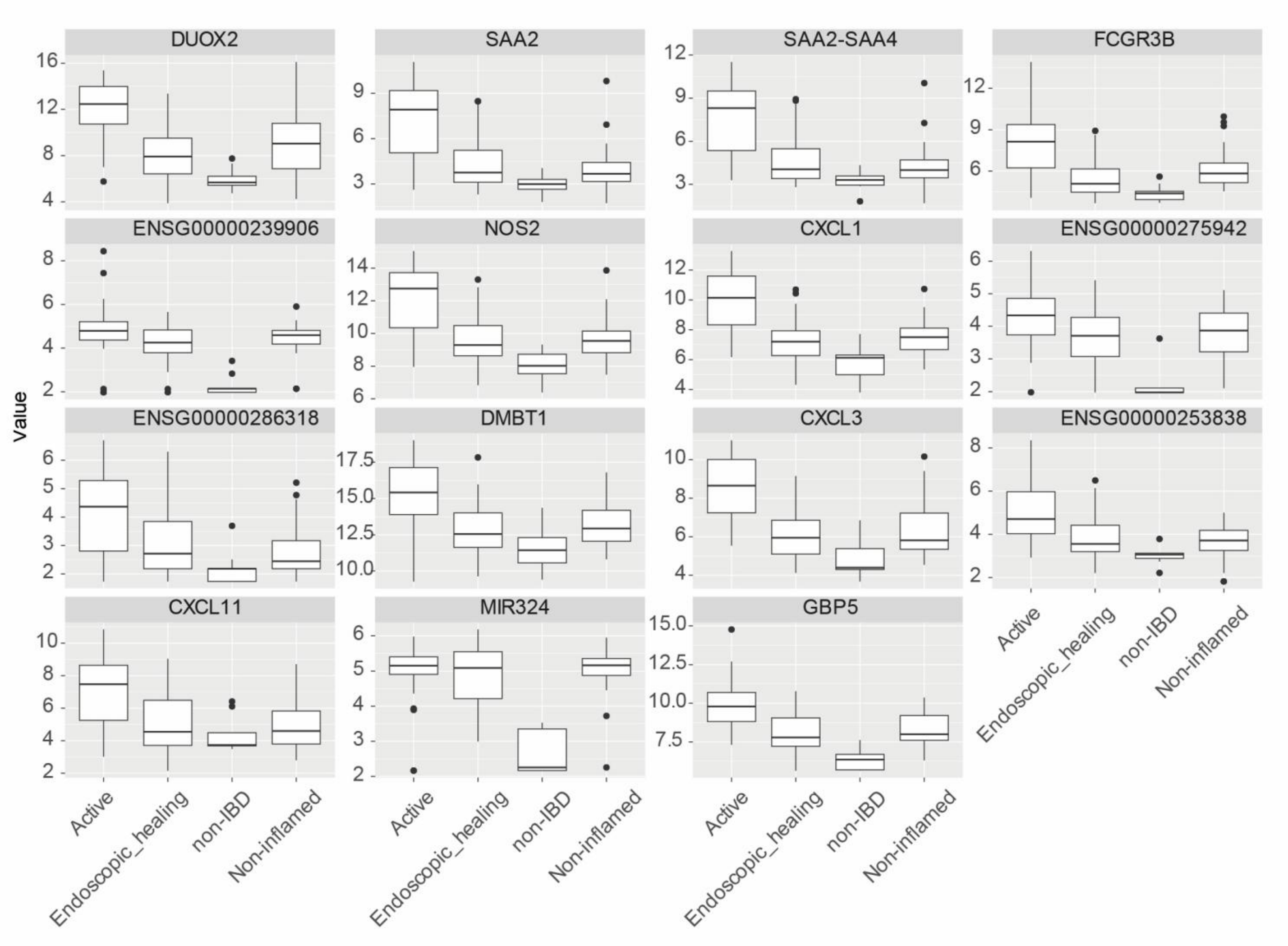

Figure S2. *Boxplots of top 15 up-regulated persistent genes according to endoscopic IBD activity (active, endoscopic healing (EH), non-inflamed) and compared to non-IBD controls (samples n = 127; IBD patients, N = 30; non-IBD controls, N = 5). Related to Figure 1.*

Figure S3

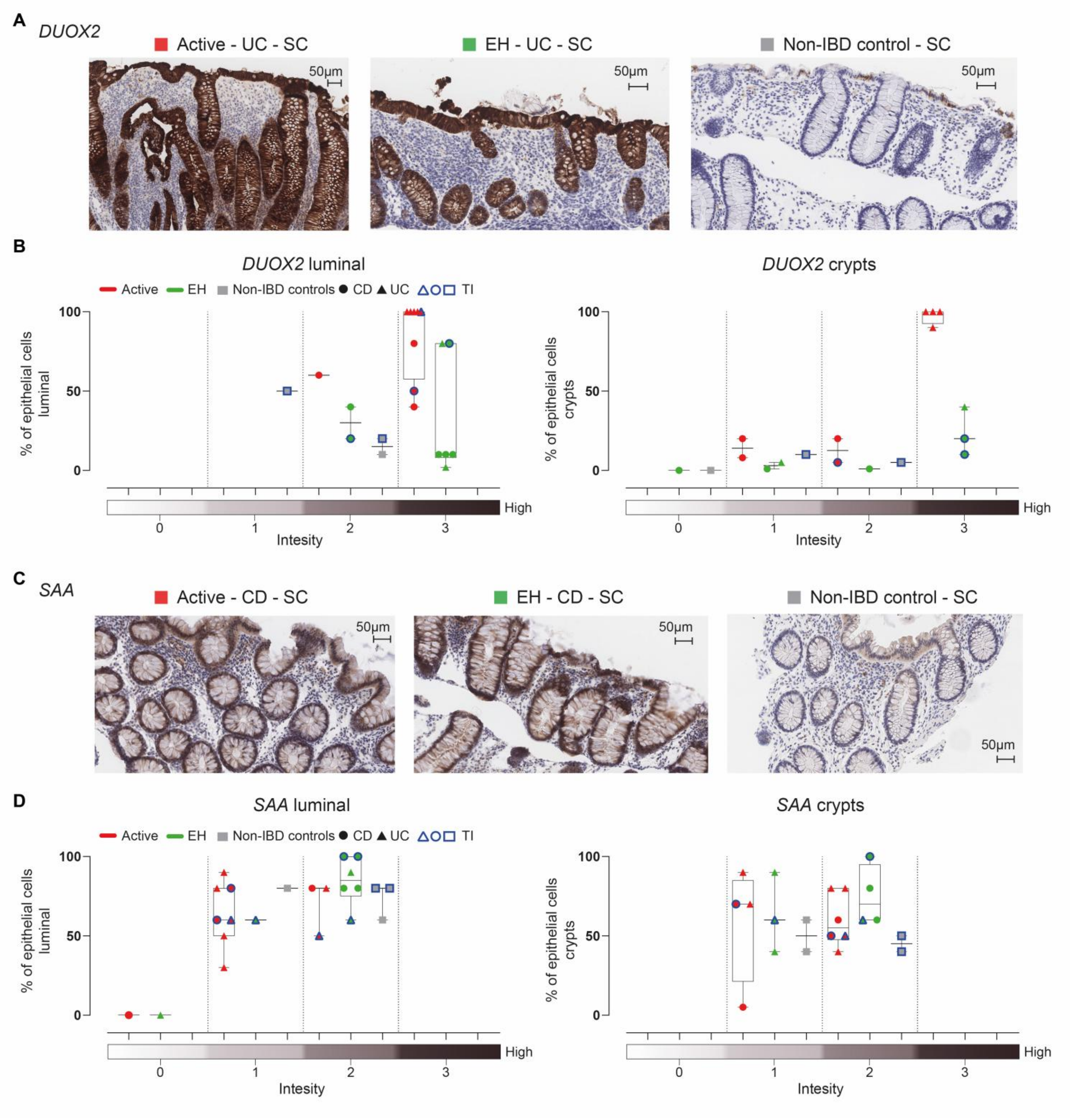

Figure S3. ***(A-B)*** *Immunohistochemistry (IHC) of DUOX2 in IBD patients (N=9, active (n=9) and endoscopic healing (EH, n=8)) and non-IBD controls (n=3).* ***(A)*** *Representative visualization of the IHC in an UC patient (sigmoid colon (SC), active and EH) and a non-IBD control.* ***(B)*** *Semi-quantification of staining intensity (from 0 [no staining] to 3 [high]) and frequency of epithelial cells (active: red; EH: green, non-IBD controls: gray, CD: circle, UC: triangle, terminal ileum (TI): blue frame, SC: no blue frame) differentiated by localization of cells in luminal (left panel) or crypts (right panel). Semi-quantification was performed by a blinded pathologist.* ***(C-D)*** *IHC of SAA.* ***(C)*** *Representative visualization of the IHC in a CD patient (SC, active and EH) and a non-IBD control.* ***(D)*** *Semi-quantification as described in (B) for IBD patients (N=9, active (n=11) and EH (n=8)) and non-IBD controls (n=4). Related to Figure 1.*

Figure S4

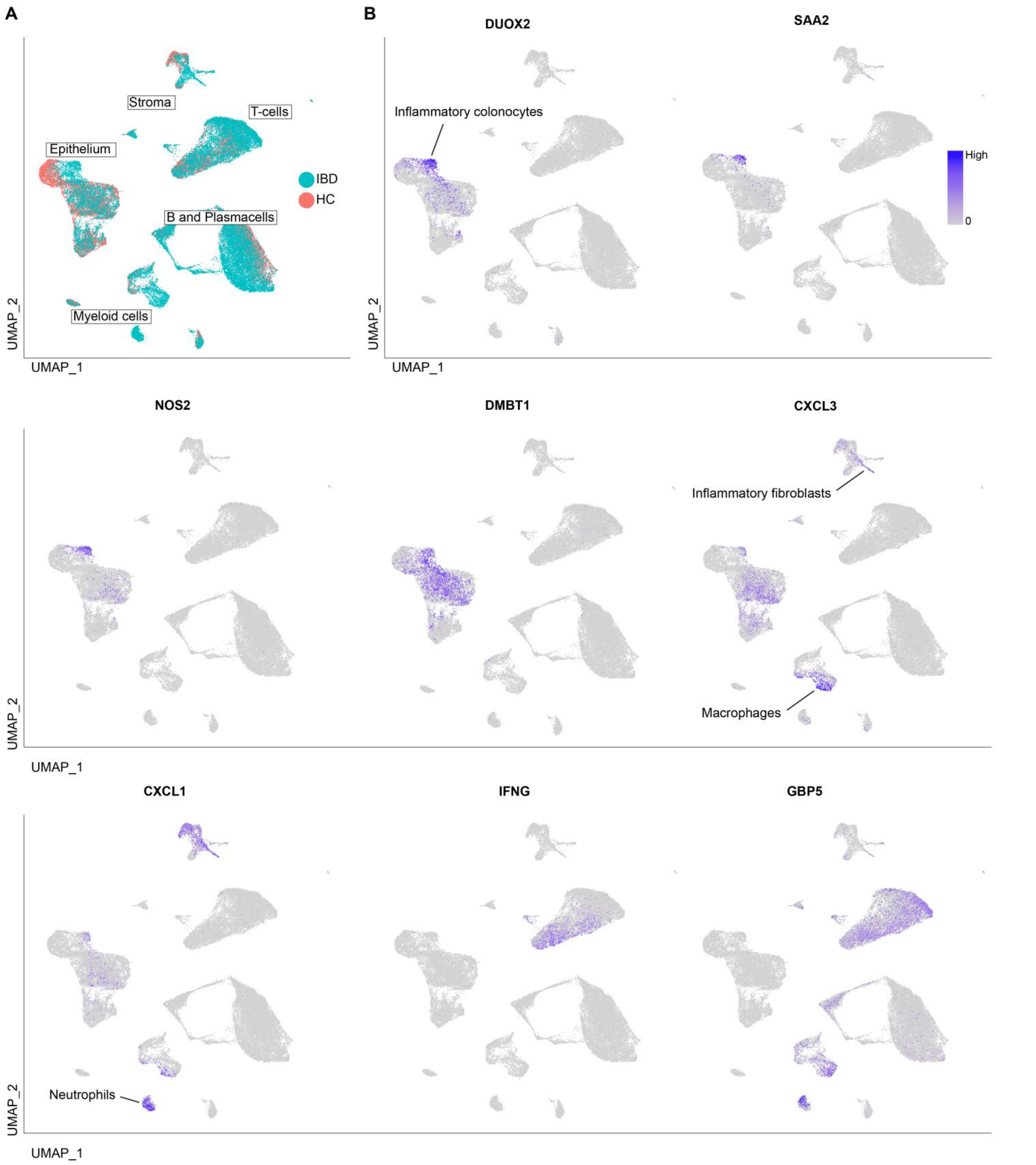

Figure S4. *External single cell data from Garrido-Trigo et al. (2023); graphs were generated at https://servidor2-ciberehd.upc.es/external/garrido/app/, accessed 08.09.2024.* ***(A)*** *The Uniform Manifold Approximation and Projection (UMAP) demonstrates the clustering of epithelial cells, T-cells, myeloid cells, stromal cells, B and plasma cells. Cells are from IBD patients (turquoise) and healthy controls (HC, red). (**B**) Feature plots show selected gene expressions of cells across the UMAP (from A). Related to Figure 1.*

Figure S5

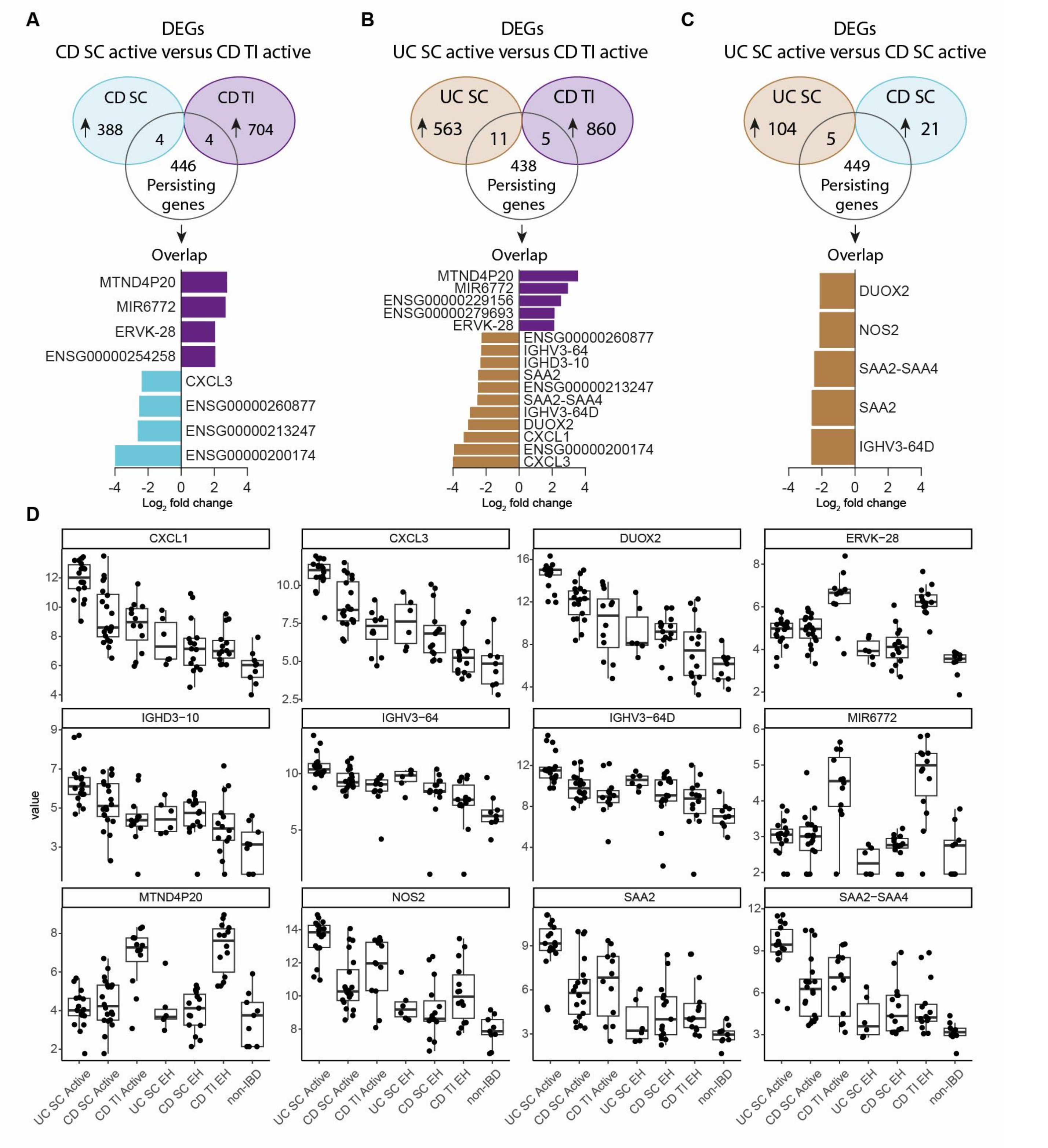

Figure S5. (A-C*)* *Testing for differentially expressed genes (DEGs; log2FC ≥ 2, ≤ -2 and p.adj < 0.05, Benjamini-Hochberg corrected) in our bulk RNA sequencing data between* ***(A)*** *CD samples with active disease at the terminal ileum (TI) versus CD with active disease at the sigmoid colon (SC),* ***(B)*** *CD TI active versus UC SC active and* ***(C)*** *CD SC active versus UC SC active. The DEGs that overlap with the persistent up-regulated genes (Figure 1G) are highlighted below.* ***(D)*** *The box plots show the expression levels of the annotated genes highlighted in A, B and C across disease subtypes (samples n = 94; IBD patients, N = 30; non-IBD controls, N = 5) and endoscopic activity: active, endoscopic healing (EH) and non-IBD controls (non-IBD). Related to Figure 1. See also Table S4-6.*

Figure S6

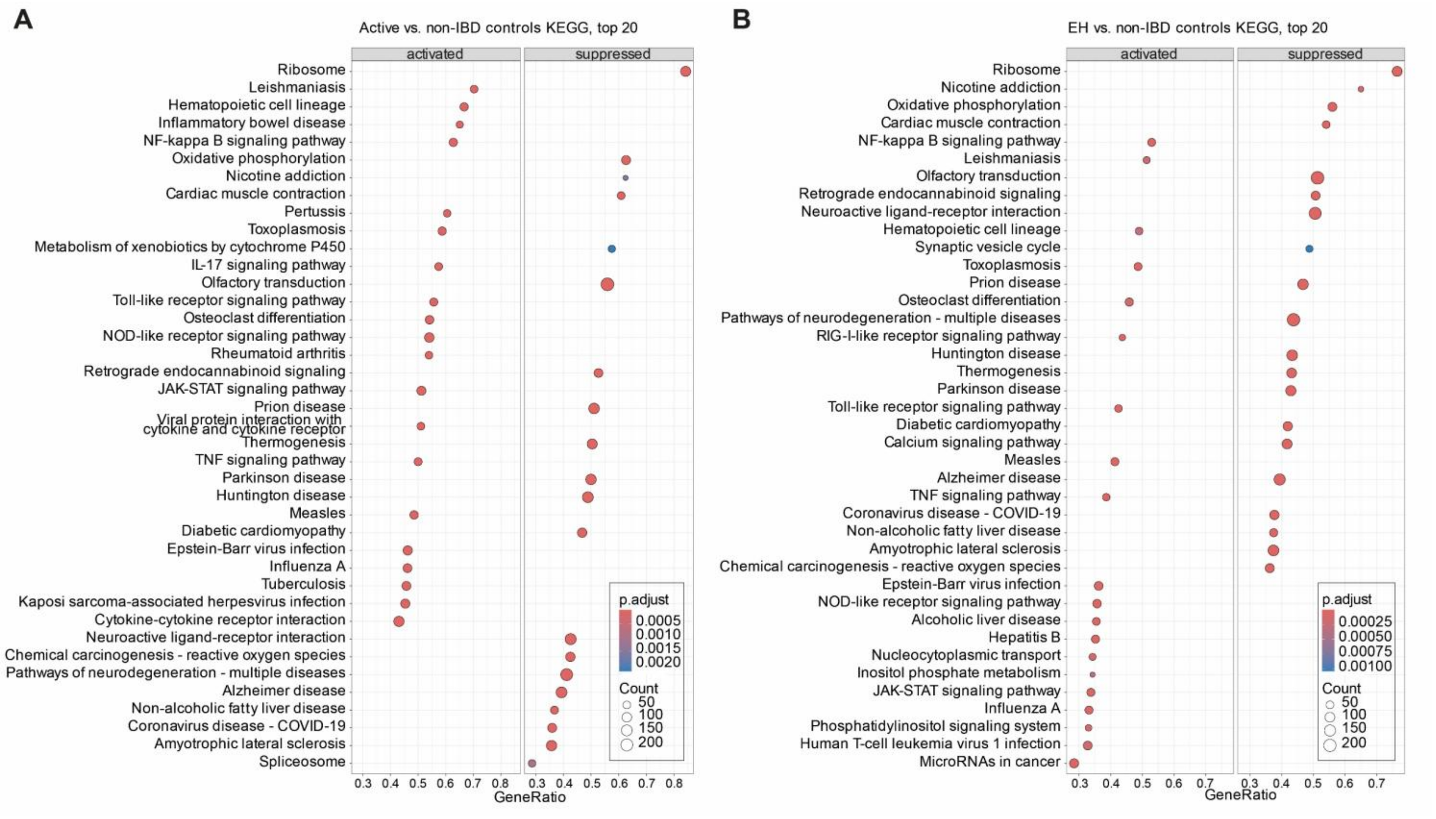

Figure S6. *Gene set enrichment analysis of* ***(A)*** *active vs. non-IBD controls and* ***(B)*** *endoscopic healing (EH) vs. non-IBD controls. Related to Figure 1.*

Figure S7

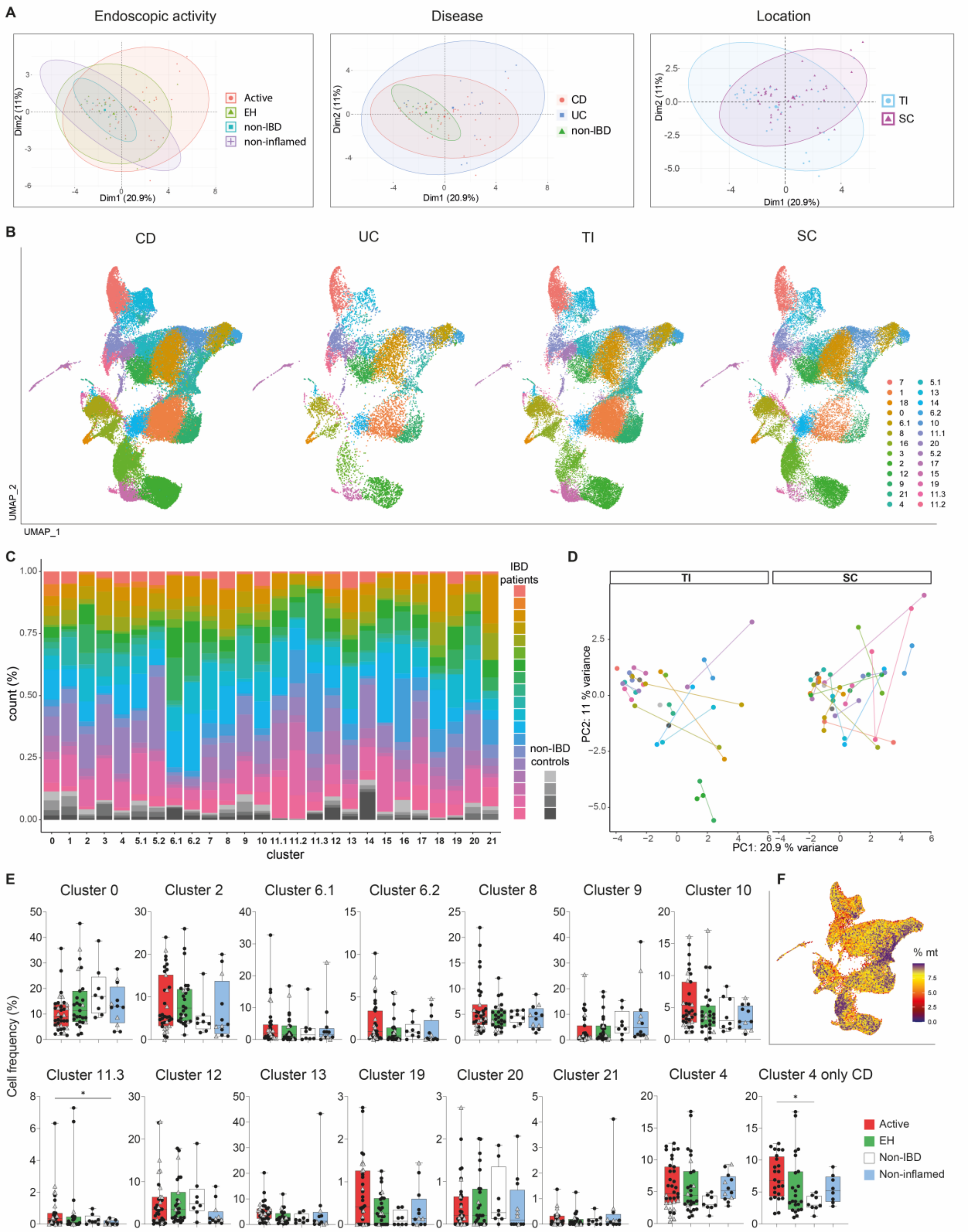

Figure S7. ***(A)*** *Principal component analysis (PCA, n = 82; IBD patients, N = 19; non-IBD controls, N = 4) showing distribution of samples according to single cell frequencies and (from left to right) endoscopic disease activity, IBD phenotype and location.* ***(B)*** *Distribution of cells highlighted for Crohn's disease (CD), ulcerative colitis (UC), terminal ileum (TI) and sigmoid colon (SC) across the UMAP (from Figure 2A).* ***(C)*** *Bar plot showing distribution of patients across all T-cell clusters. There was no over-representation of certain clusters in individual patients.* ***(D)*** *PCA of single T-cell frequencies, showing individual changes over time for each location (TI, SC).* ***(E)*** *Boxplots showing relative cell frequencies per cluster and biopsy (per individual biopsy: number of cells per cluster/total cell number per biopsy, CD: circles, UC: gray triangles) according to endoscopic activity groups; cluster 4 is shown additionally for CD only. Statistical tests: Kruskal-Wallis test followed by comparing mean ranks between individual columns and multiple testing correction using Benjamini-Hochberg, p.adj < 0.05, *=significant; non-significant is not labeled.* ***(F)*** *Feature plot showing percent of mitochondrial (mt) gene expression (QC cut-off: 10%) across T-cell clusters. Related to Figure 2.*

Figure S8

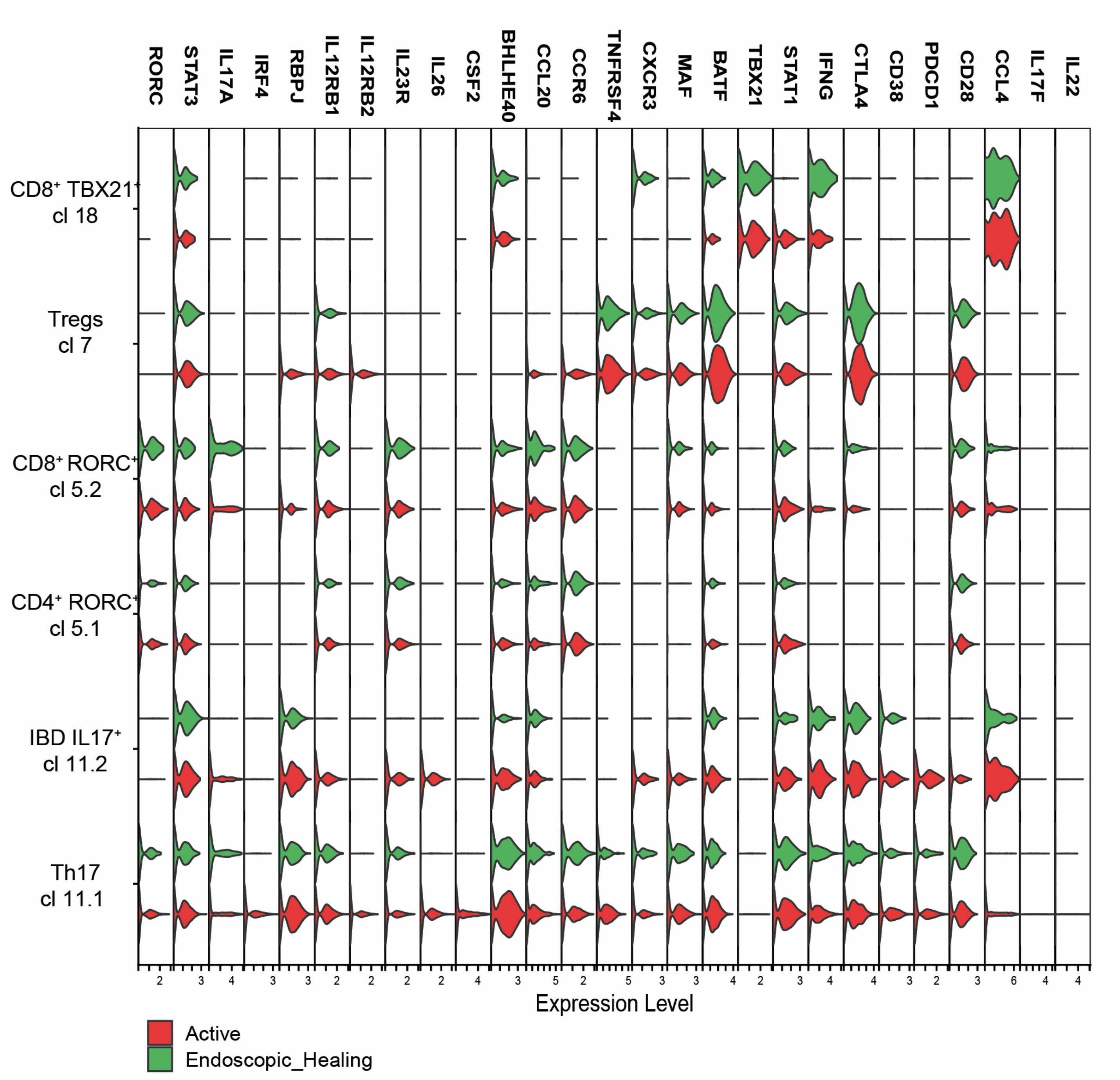

Figure S8. *Violin plot showing gene expression of selected clusters and selected genes according to disease activity groups (active in red; endoscopic healing in green). Related to Figure 2.*

Figure S9

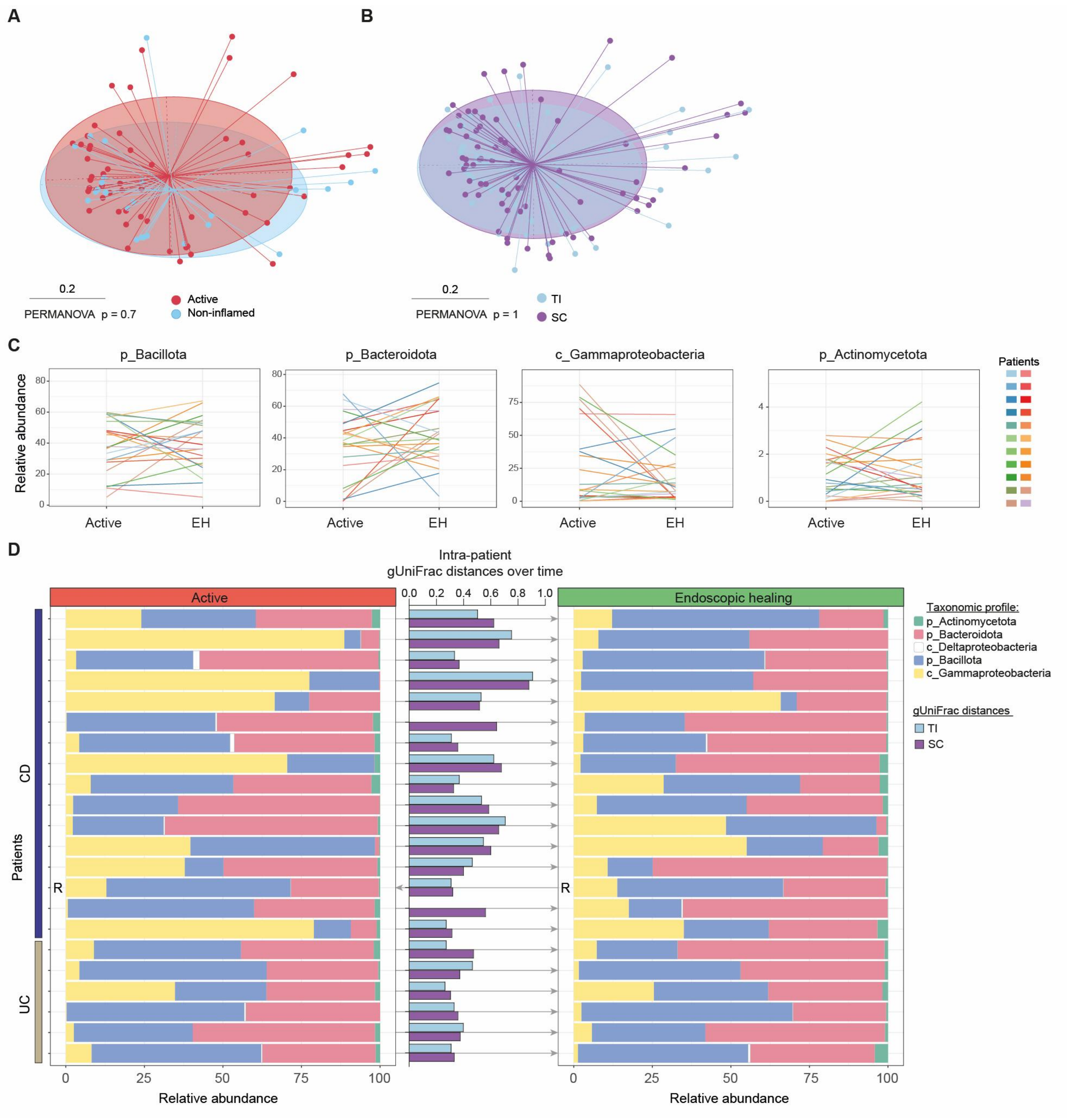

Figure S9. ***(A-B)*** *Beta-diversity MDS plots of* ***(A)*** *active (n=56) vs. non-inflamed (non-inflamed region in an active disease state, n=26) IBD and of* ***(B)*** *anatomic location (terminal ileum (TI, n=62) and sigmoid colon (SC, n=73)).* ***(C)*** *Time-series analysis between active and EH of individual patients (N=22) of Bacillota, Bacteroidota, Actinomycetota and Gammaproteobacteria, showing the mean (TI and SC) relative abundance for active and EH, respectively.* ***(D)*** *Mean taxonomic profile changes of individual patients between active and EH (N=22) as color-coded stacked bar plots. In addition, intra-patient generalized UniFrac (gUniFrac) distances with location specific changes between active and EH are shown. The index patient, who experienced a relapse (T_{F1}) within 10 weeks after discontinuing anti-TNF medication following EH (T_{BL}) is highlighted with an R. For the CD patient who first reached EH at T_{F2}, T_{BL} and T_{F2} are shown. Related to Figure 4.*

Figure S10

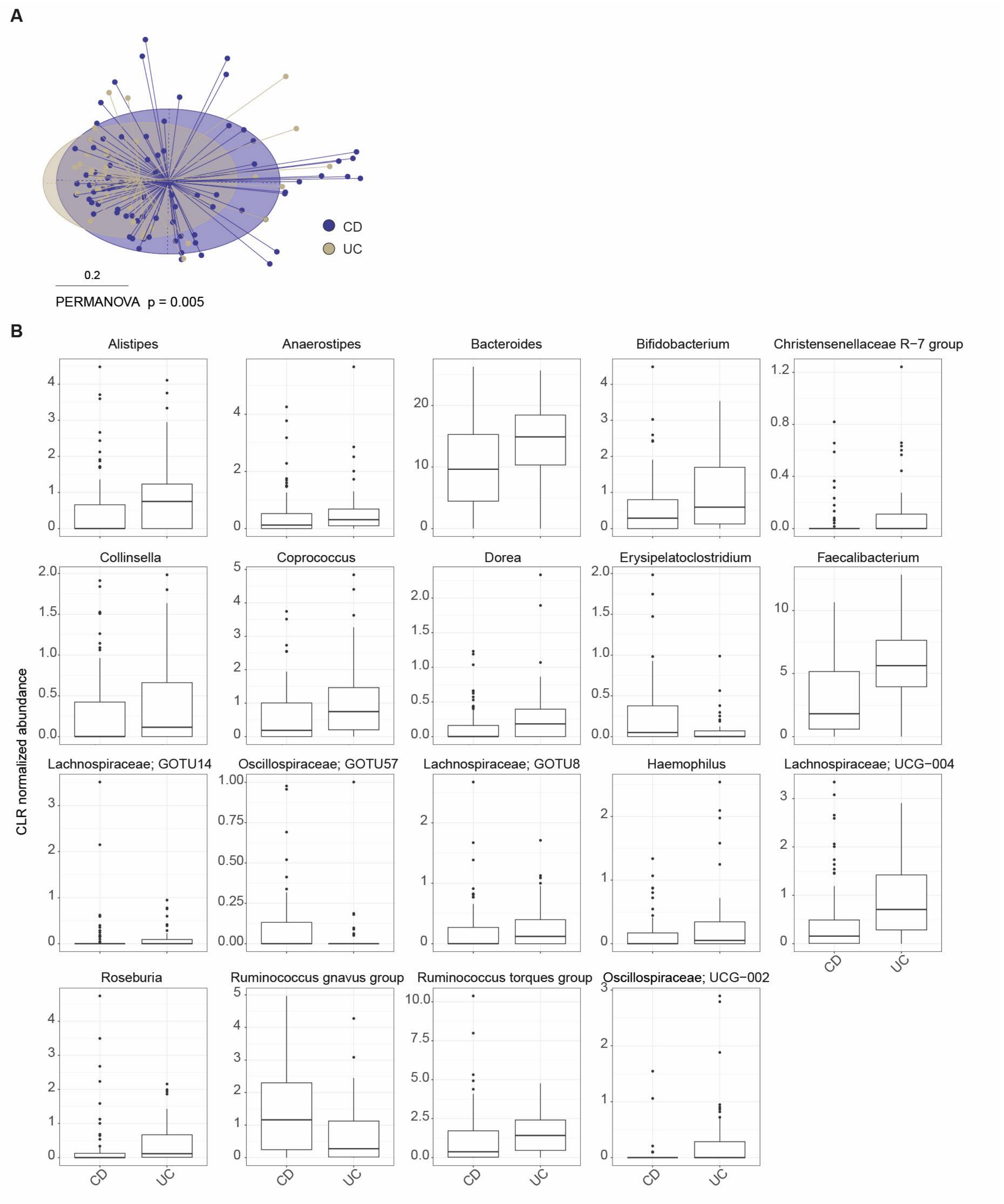

Figure S10 (A) *Beta-diversity MDS plot (generalized UniFrac distances) of IBD phenotype groups (CD, n=82; UC, n=43).* ***(B)*** *Boxplots showing centered log-ratio (CLR) transformed abundance of significantly different genera between CD and UC (Wilcoxon test, significance cutoff = 0.05). Related to Figure 4.*

Figure S11

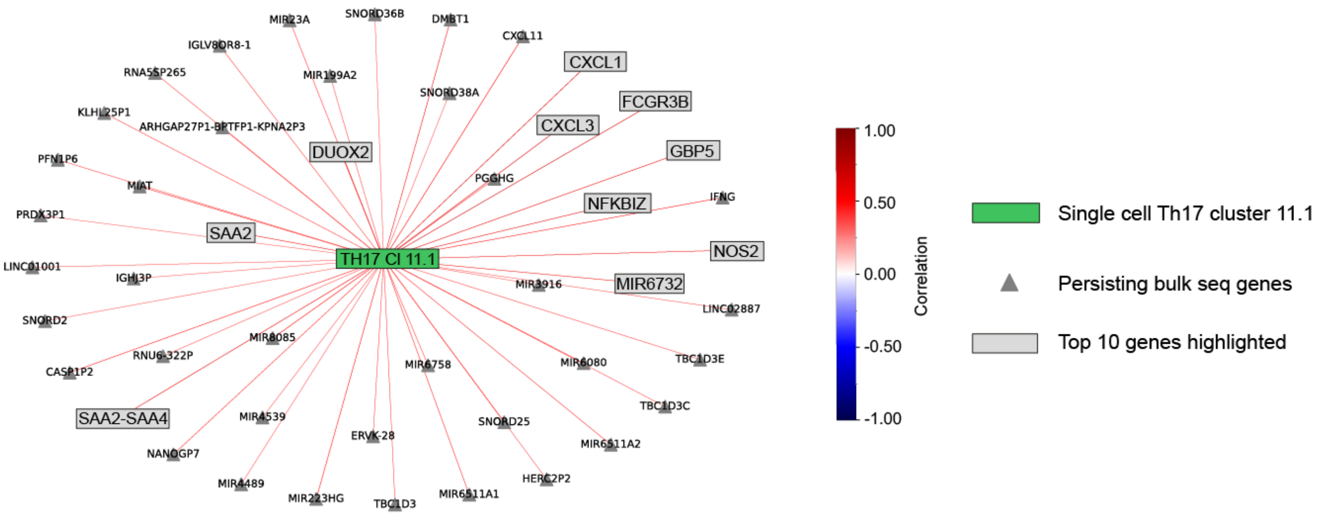

Figure S11. *Correlation network between Th17 cells (cluster 11.1 from Figure 2) and 282 persistently up-regulated genes (with official gene symbol) from bulk RNA sequencing (Figure 1G): nodes, identified significant features (top 10 genes according to correlation and p.adj-values are highlighted in gray boxes); edges, significant (Spearman) correlations between features of the two modalities (red, positive; blue, negative). Related to Figure 5.*

Figure S12

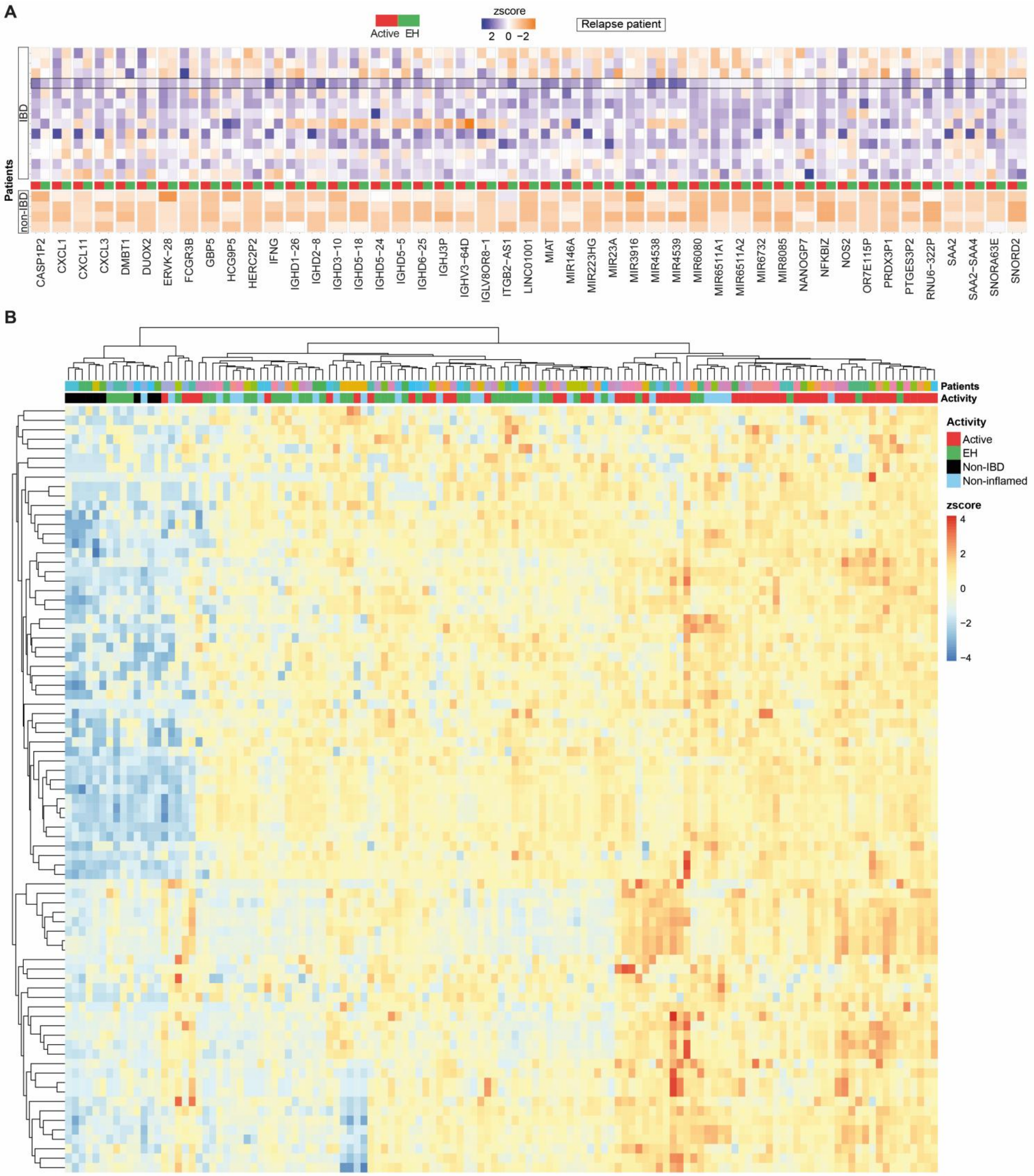

Figure S12. ***(A)*** *Individual expression of IBrD signature genes (>1 edge, from Figure 5B) in IBD patients and in non-IBD controls. For different anatomical locations, but with corresponding disease activity (e.g., active), the average value was computed. Subsequently, mean gene expression values were z-score scaled for visualization.* ***(B)*** *The heatmap of our bulk RNA sequencing samples (samples n = 127; IBD patients, N = 30; non-IBD controls, N = 5) illustrates the expression levels of the 81 IBrD signature genes across individual biopsies of patients, including hierarchical clustering. The biopsy origin according to patient and disease activity is highlighted. Related to Figure 5.*

Figure S13

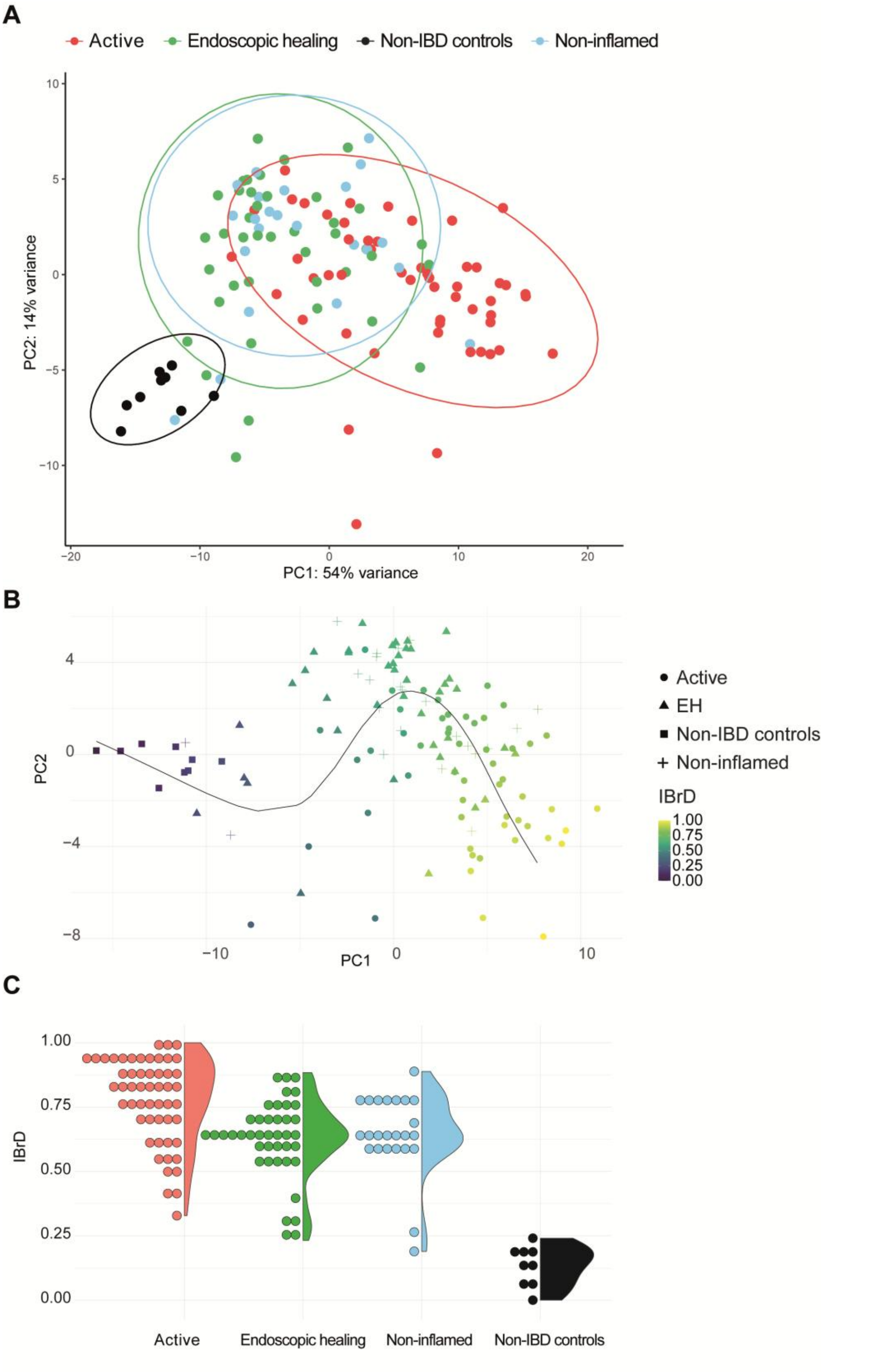

Figure S13. ***(A)*** *Principal component analysis (PCA, corrected for batch and location) showing distribution of bulk RNA sequencing samples (n=127) based on the IBrD signature.* ***(B)*** *PCA as in Figure 5D, including all bulk RNA samples (n=127), with color code indicating the Inflammatory Bowel residual Disease (IBrD, detailed description of how the IBrD was calculated for all samples are provided in the methods) scaled between 0 to 1, where 1 indicates highest activity.* ***(C)*** *IBrD values (interval between 0 and 1) for each sample according to endoscopic activity. Related to Figure 5.*

Figure S14

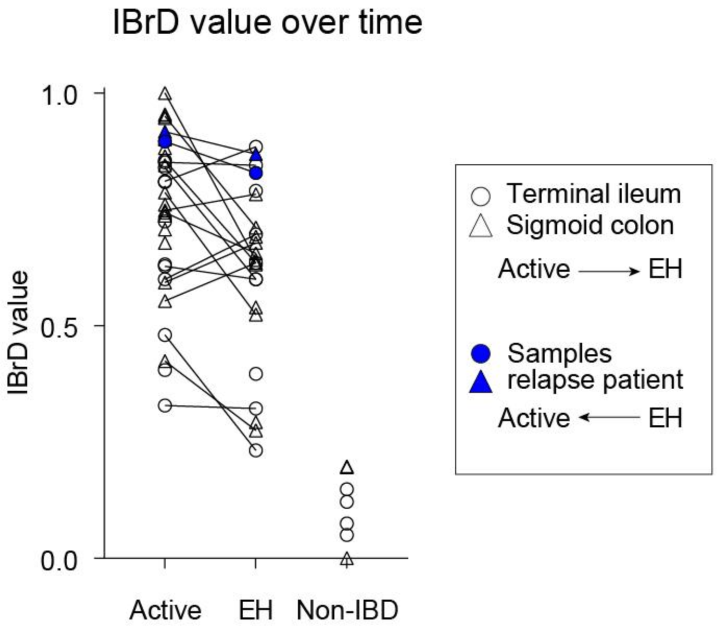

Figure S14. *Patient- and biopsy-specific differences of the IBrD over time (index patient, who experienced a relapse (T_F) within 10 weeks after discontinuing anti-TNF medication following EH (T_{BL}) is highlighted in blue). Active, n=33; EH, n=24; non-IBD controls, n=7. Related to Figure 5.*
